# Global burden of childhood Burkitt lymphoma (1990–2021): epidemiological trends, regional disparities, and projections for 2035 from the Global Burden of Disease Study

**DOI:** 10.3389/fmed.2025.1619750

**Published:** 2025-09-24

**Authors:** Guoqian Ma, Changyu Hou, Yuan Li, Fan Jia

**Affiliations:** ^1^Department of Cardiology/Key Laboratory of Cardiovascular Disease of Yunnan Province/Clinical Medicine Center for Cardiovascular Disease of Yunnan Province, Yan’an Affiliated Hospital of Kunming Medical University, Kunming, China; ^2^Department of Pediatrics, Yunnan Diannan Central Hospital (The First People’s Hospital of Honghe Prefecture), Kunming, China; ^3^Ultrasound Department, Kunming Maternal and Child Health Hospital, Kunming, China

**Keywords:** Burkitt lymphoma (BL), children, Global Burden of Disease (GBD) 2021, incidence, DALYs, mortality

## Abstract

**Background:**

Burkitt lymphoma (BL) is a highly aggressive pediatric B-cell lymphoma with marked geographic heterogeneity. Up-to-date global estimates are needed to inform prevention and care.

**Methods:**

We analyzed Global Burden of Disease (GBD) 2021 data to estimate incidence, mortality, and disability-adjusted life years (DALYs) for childhood BL (0–14 years) from 1990 to 2021 across 204 countries/territories, stratified by sex, age, region, and Sociodemographic Index (SDI). Trends were summarized using joinpoint annual percent change and estimated annual percentage change (EAPC). Bayesian age-period-cohort models projected the burden to 2035.

**Findings:**

In 2021, there were 4,083 incident BL cases globally [95% uncertainty interval (UI) 2,688–5,171]. The global incidence rate was 0.20 per 100,000 (95% UI 0.13–0.26) versus 0.16 per 100,000 (95% UI 0.10–0.23) in 1990. Burden varied substantially by development level: the low-SDI region had the highest incidence rate (0.44 per 100,000, 95% UI 0.26–0.59), mortality rate (0.43 per 100,000, 95% UI 0.25–0.58), and DALY rate (36.19 per 100,000, 95% UI 21.00–48.64). High-SDI regions showed lower mortality rates and evidence of declines, consistent with broader access to timely diagnosis, multi-agent chemotherapy, and supportive care. By 2035, the incidence rate is expected to reach 0.15 (95% CI 0.11–0.19) per 100,000, the mortality rate to 0.10 (95% CI 0.07–0.13) per 100,000, and the DALY rate to 8.21 (95% CI 5.98–10.43) per 100,000.

**Interpretation:**

Global BL rates appear broadly stable, with pronounced inequities concentrated in low-SDI settings. Apparent improvements are heterogeneous, not universal, and are most evident in high-SDI regions where treatment and supportive care are widely available. Projections to 2035 suggest modest declines in global rates, contingent on sustained malaria control and expanded access to diagnosis and curative therapy in high-burden regions. Targeted investments linking infection control with pediatric oncology capacity are required to reduce the global burden of childhood BL.

## Introduction

Burkitt lymphoma (BL) is a highly aggressive B-cell non-Hodgkin lymphoma and is often cited as the fastest-growing human tumor (1, 2). First described by Denis Burkitt in 1958 in Ugandan children (3), BL was also one of the first cancers linked to both a viral infection and a chromosomal oncogene activation (Epstein–Barr virus infection and the characteristic MYC translocation) (4, 5). Three primary epidemiological variants of BL are recognized: endemic BL in equatorial Africa (and Papua New Guinea), sporadic BL and immunodeficiency-associated BL occurring in the context of HIV/AIDS or other immunosuppression (6–8). Endemic BL corresponds to the regions of highest BL incidence globally, historically, it was observed that BL is the most common childhood cancer in areas with holoendemic malaria (8). In these high-risk regions, BL can account for up to 30–50% of all pediatric malignancies (9). Chronic exposure to Plasmodium falciparum malaria and early-life Epstein–Barr virus (EBV) infection are key cofactors in endemic BL, and indeed, >90% of endemic BL cases harbor EBV in the tumor cells (5, 7, 10). By contrast, BL arising with HIV infection also occurs globally (especially in non-endemic areas), often presenting in older patients (11). Despite its aggressive nature, BL is highly chemosensitive and potentially curable—multi-agent chemotherapy (often combined with rituximab immunotherapy) can achieve long-term survival in the majority of pediatric BL patients in high-income countries (12). Cure rates around 90% have been reported in contemporary trials in high-resource settings (13).

However, outcomes remain much poorer in low- and middle-income countries, where BL is often diagnosed late and treated with less intensive regimens, affecting the overall good outcomes in patients with this lymphoma (1). The success of intensive therapy also depends on supportive care infrastructure that is often lacking in resource-limited settings, further contributing to the outcome gap. This stark contrast highlights BL as an important global health challenge: it is a highly curable pediatric cancer when adequate therapy is available, yet it continues to cause high mortality in many parts of the world.

To date, no comprehensive global study has reported trends in childhood BL incidence and mortality. The latest Global Burden of Disease (GBD) 2021 study provides updated data on BL’s worldwide burden (14). This study aims to fill that gap by examining the epidemiological trends of childhood BL from 1990 to 2021, with a particular focus on regional disparities, gender differences, and the evolving burden of the disease. Additionally, we aim to provide projections for 2035 to inform global health policies and strategies aimed at reducing the burden of BL, particularly in high-risk areas.

## Methods

### Overview and methodological details

The GBD database represents one of the most comprehensive and systematic epidemiological resources globally. Managed by the Institute for Health Metrics and Evaluation (IHME) at the University of Washington, the GBD project aims to quantify health losses attributable to various diseases, injuries, and risk factors. The GBD framework facilitates comparative assessments of incidence, mortality, and disability-adjusted life years (DALYs) across countries, regions, and globally (15). In GBD analyses, disease burden is quantified using three key metrics: incidence, mortality, and DALYs. DALYs represent the sum of Years of Life Lost (YLL) due to premature mortality and Years Lived with Disability (YLD). These metrics are calculated using the following formulas:


$$\begin{array}{l} \mathrm{YLL}=\text{Number of deaths}\times \text{standard life}\\ \;\;\;\;\;\;\;\;\;\;\;\;\;\;\;\;\text{expectancy}\;\mathrm{at}\;\text{the}\;\mathrm{age}\;\text{of death} \end{array}$$

YLD=Prevalence of the condition×disability weight

Disability weights, determined through expert consensus, range from 0 (perfect health) to 1 (death). This methodological approach enables robust scientific evaluations of disease burdens (16). The present study analyzed GBD data of the incidence, mortality, and DALYs of BL among children aged 0–14 years from 1990 to 2021, sourced from the GBD results tool^1^ on 3 March 2025. The analysis covered 204 countries and territories and included stratification by gender, age groups (under 1 year, 1–2, 2–4, 5–9, and 10–14 years), and geographic location. Due to data limitations within the GBD database, ethnicity- and race-related analyses were not conducted. Ethical approval was waived by the Ethics Committee of Kunming Yan’an Hospital, as the cross-sectional nature of this study involved secondary analysis of aggregated and anonymized data. The study strictly adhered to the Strengthening the Reporting of Observational Studies in Epidemiology (STROBE) guidelines (17).

### Sociodemographic Index

The Sociodemographic Index (SDI) is a composite metric assessing socioeconomic development across countries or regions. It integrates factors including economic structure, educational attainment, standard of living, and social welfare, with values ranging from 0 (lowest socioeconomic status) to 1 (highest socioeconomic status) (18). According to the GBD framework, countries and regions are classified into five SDI categories: low, low-middle, middle, high-middle, and high. This stratification facilitates the assessment of the impact of socioeconomic and geographic disparities on childhood BL burden.

### Bayesian age-period-cohort model projection

The Bayesian age-period-cohort (BAPC) model extends the traditional generalized linear model (GLM) framework within a Bayesian context, enabling the dynamic integration of age, period, and cohort effects. The model assumes temporal evolution of these effects, applying second-order random walks for smoothing and generating precise posterior predictions. A significant advantage of the BAPC model is its use of integrated nested Laplace approximations (INLA) to estimate marginal posterior distributions efficiently, thus circumventing convergence and mixing issues commonly associated with Markov Chain Monte Carlo (MCMC) techniques. Due to its flexibility and robustness, particularly in handling longitudinal data with complex cohort effects, the BAPC model is extensively validated and employed in epidemiological studies (19). In this study, we applied the BAPC model to project the global burden of childhood BL through 2035. This timeframe was chosen to offer a near-term perspective useful for public health planning while extending far enough to capture meaningful trends beyond short-term fluctuations.

### Statistical analysis

Incidence, mortality, and DALY rates per 100,000 individuals were calculated along with their respective 95% uncertainty intervals (UIs) based on the GBD data. Temporal trends were evaluated using joinpoint regression to estimate annual percentage changes (APCs) and corresponding 95% confidence intervals (CIs) (20), providing granular insights into year-on-year fluctuations. Additionally, long-term trends from 1990 to 2021 were assessed using log-transformed linear regression models to calculate estimated annual percentage changes (EAPCs) and their 95% CIs (21). Positive EAPC values, with lower confidence bounds above zero, indicated increasing trends, while negative values, with upper confidence bounds below zero, denoted declining trends. All statistical analyses were conducted using R software (version 4.4.2), with statistical significance defined as *p* < 0.05.

## Results

### Global trends

#### Incidence

**Figure 1:**
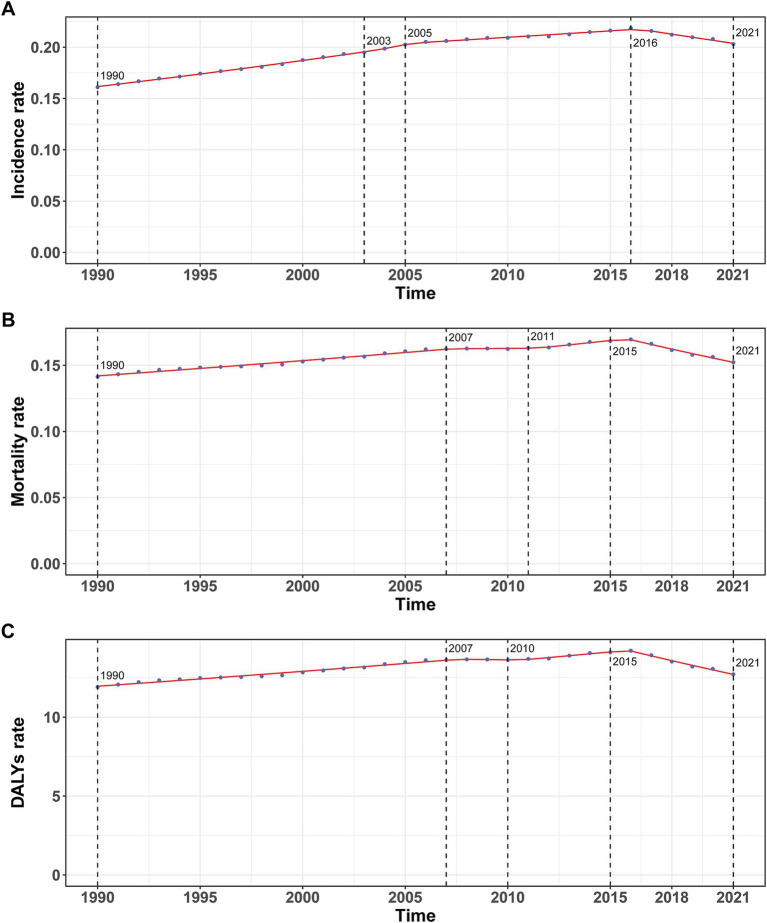
Annual percent change (APC) and trends in global childhood Burkitt’s lymphoma incidence, mortality, and disability-adjusted life years (DALYs) from 1990 to 2021. **(A)** Incidence rate. **(B)** Mortality rate. **(C)** DALYs rate.

**Figure 2:**
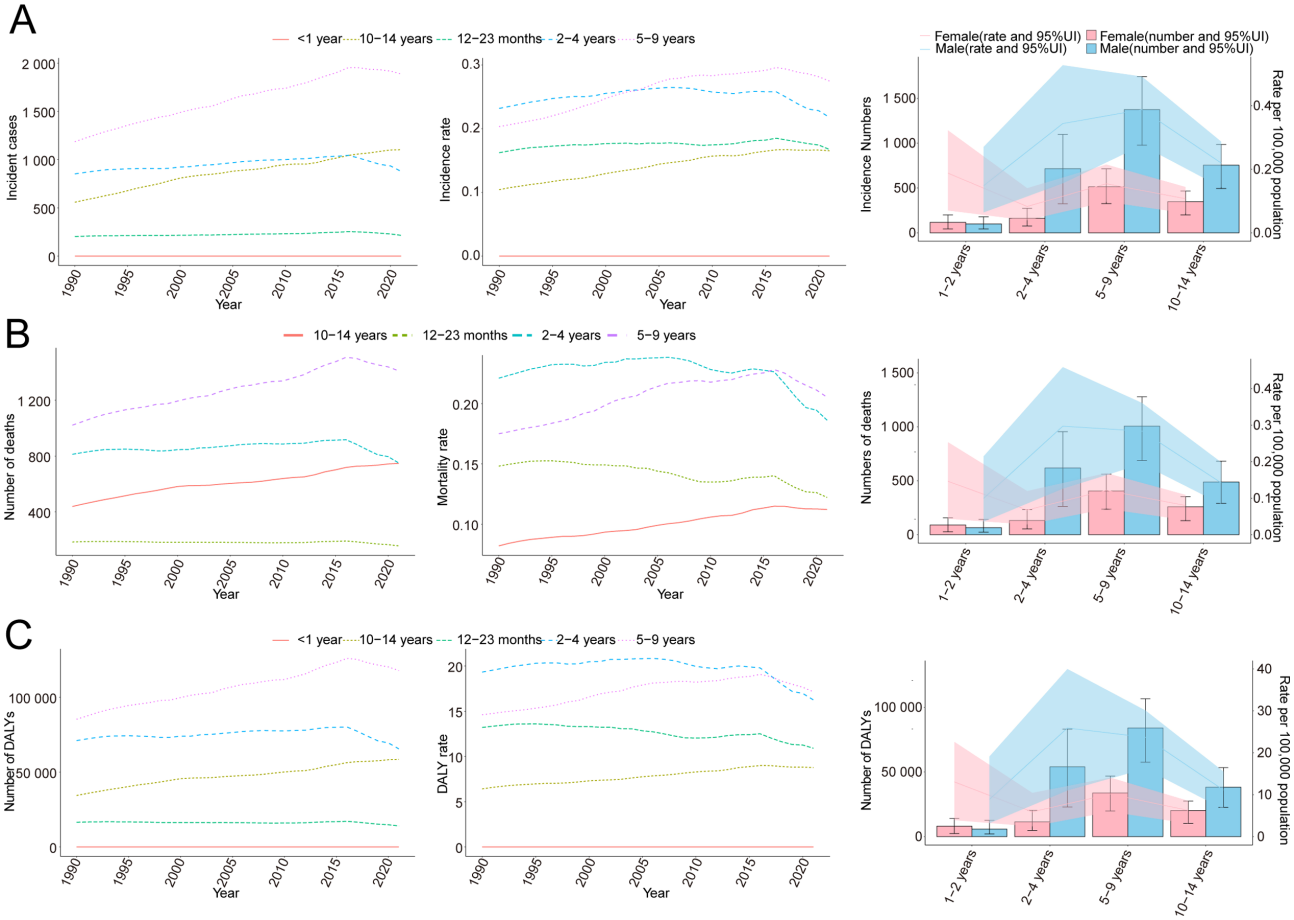
Trends in incidence, mortality, and disability-adjusted life years (DALYs) of childhood Burkitt’s lymphoma by age and sex, 1990–2021. **(A)** Incidence cases and rates. **(B)** Mortality cases and rates. **(C)** DALYs cases and rates.

**Figure 3:**
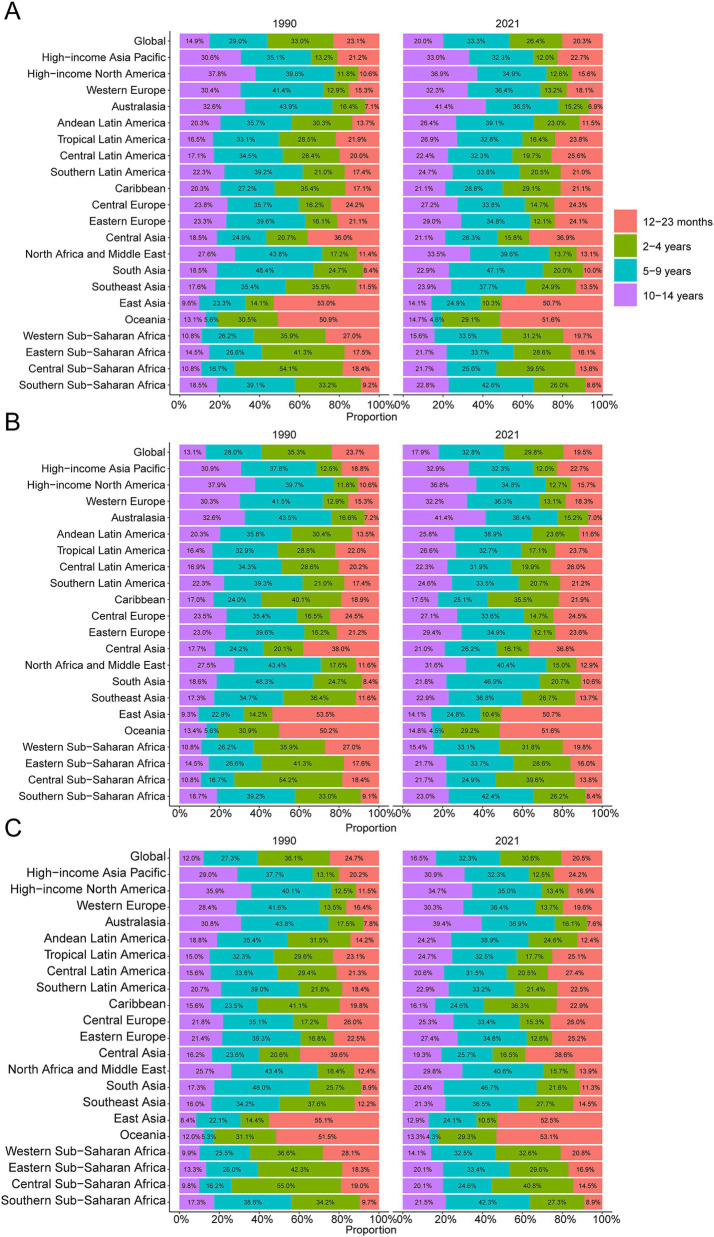
Age-specific percentages of childhood Burkitt’s lymphoma incidence, mortality, and disability-adjusted life years (DALYs) in 1990 and 2021. **(A)** Incidence. **(B)** Deaths. **(C)** DALYs.

In 2021, the global incidence rate of childhood BL was 0.20 per 100,000 (95% UI 0.13–0.26) versus 0.16 per 100,000 (95% UI 0.10–0.23) in 1990 (Table 1). Rates peaked around 2016 (0.22 per 100,000, 95% UI 0.14–0.27) and were lower in 2021 (Figure 1A), indicating broad stability with modest fluctuations rather than a monotonic rise. Incidence remained concentrated in school-age children, with the largest proportion in 5–9 years in 2021. A consistent male predominance persisted across ages. For example, among 2–4-year-olds in 2021, boys had an incidence rate of 0.34 per 100,000 (95% UI 0.16–0.53) versus 0.08 per 100,000 (95% UI 0.04–0.14) in girls (Figure 2A). In 1990, the highest incidence rate was observed among 2–4-year-olds, accounting for 33.0% of total cases. By 2021, however, the peak incidence had shifted to the 5–9-year-old group, representing 33.3% of all cases (Figure 3A).

**Table 1:** Incidence of Burkitt lymphoma in children between 1990 and 2021 at the global and regional levels.

Location	1990		2021		1990–2021	
	Incident cases	Incidence rate	Incident cases	Incidence rate	Cases change	EAPC^a^
Global	2799.73 (1677.88, 3942.62)	0.16 (0.10, 0.23)	4083.40 (2687.86, 5170.75)	0.20 (0.13, 0.26)	45.85 (9.37, 101.83)	0.92 (0.78, 1.07)
SDI						
High SDI	245.04 (170.35, 359.66)	0.13 (0.09, 0.19)	306.27 (151.47, 423.00)	0.18 (0.09, 0.25)	24.99 (−39.22, 66.48)	1.11 (0.58, 1.64)
High-middle SDI	261.54 (162.59, 368.65)	0.10 (0.06, 0.13)	322.62 (198.85, 428.37)	0.14 (0.09, 0.19)	23.36 (−31.90, 100.37)	1.42 (1.10, 1.74)
Middle SDI	350.26 (218.25, 477.13)	0.06 (0.04, 0.08)	555.79 (329.71, 770.91)	0.10 (0.06, 0.14)	58.68 (−10.86, 129.00)	1.66 (1.49, 1.83)
Low-middle SDI	567.86 (295.21, 826.35)	0.12 (0.06, 0.18)	874.38 (588.64, 1184.65)	0.15 (0.10, 0.20)	53.98 (3.62, 145.46)	0.76 (0.68, 0.85)
Low SDI	1373.09 (645.73, 2153.06)	0.60 (0.28, 0.94)	2021.87 (1195.73, 2717.88)	0.44 (0.26, 0.59)	47.25 (5.46, 121.38)	−0.86 (−0.94, −0.79)
Regions						
Andean Latin America	17.92 (11.20, 29.07)	0.12 (0.08, 0.20)	31.87 (17.70, 50.15)	0.18 (0.10, 0.28)	77.89 (−25.84, 240.76)	1.28 (1.11, 1.46)
Australasia	7.76 (4.89, 11.90)	0.17 (0.11, 0.26)	11.34 (5.45, 18.13)	0.20 (0.10, 0.32)	46.21 (−34.56, 146.43)	0.37 (−0.39, 1.14)
Caribbean	23.26 (13.81, 41.89)	0.20 (0.12, 0.37)	19.22 (9.30, 33.23)	0.17 (0.08, 0.29)	−17.34 (−56.49, 39.67)	0.40 (0.08, 0.73)
Central Asia	7.89 (4.59, 13.83)	0.03 (0.02, 0.06)	6.86 (3.91, 10.94)	0.02 (0.01, 0.04)	−13.07 (−58.30, 65.03)	−0.79 (−1.46, −0.11)
Central Europe	16.62 (10.54, 29.73)	0.06 (0.04, 0.10)	20.35 (8.92, 29.39)	0.11 (0.05, 0.17)	22.49 (−60.00, 113.17)	2.55 (1.88, 3.22)
Central Latin America	56.51 (40.53, 87.38)	0.09 (0.06, 0.14)	100.81 (55.70, 139.76)	0.16 (0.09, 0.22)	78.40 (−12.93, 166.86)	2.18 (1.80, 2.56)
Central Sub-Saharan Africa	132.28 (47.46, 230.34)	0.52 (0.19, 0.91)	140.60 (69.74, 209.03)	0.24 (0.12, 0.36)	6.29 (−29.48, 138.64)	−2.26 (−2.44, −2.08)
East Asia	166.36 (73.40, 262.17)	0.05 (0.02, 0.08)	177.79 (95.14, 303.58)	0.07 (0.04, 0.11)	6.86 (−50.14, 167.72)	0.07 (−0.26, 0.41)
Eastern Europe	59.79 (33.53, 91.59)	0.12 (0.07, 0.18)	40.52 (16.31, 57.79)	0.11 (0.05, 0.16)	−32.24 (−72.55, −0.20)	0.67 (0.04, 1.29)
Eastern Sub-Saharan Africa	876.59 (402.70, 1353.91)	0.97 (0.44, 1.49)	1136.86 (659.54, 1552.67)	0.64 (0.37, 0.87)	29.69 (−10.13, 110.61)	−1.24 (−1.32, −1.17)
High-income Asia Pacific	30.21 (14.86, 48.53)	0.09 (0.04, 0.14)	36.29 (16.03, 52.55)	0.16 (0.07, 0.23)	2 0.12 (−49.51, 183.51)	2.08 (1.47, 2.69)
High-income North America	122.57 (80.71, 172.63)	0.20 (0.13, 0.28)	121.89 (74.81, 171.07)	0.19 (0.11, 0.26)	−0.55 (−39.25, 31.75)	−0.01 (−0.41, 0.40)
North Africa and Middle East	125.32 (68.72, 208.22)	0.09 (0.05, 0.15)	206.48 (134.68, 305.12)	0.11 (0.07, 0.17)	64.76 (−13.32, 196.52)	0.82 (0.58, 1.06)
Oceania	0.58 (0.23, 1.23)	0.02 (0.01, 0.05)	2.04 (0.64, 4.38)	0.04 (0.01, 0.09)	252.04 (65.91, 646.50)	2.14 (1.77, 2.52)
South Asia	274.79 (98.08, 469.62)	0.06 (0.02, 0.11)	332.69 (181.38, 515.00)	0.07 (0.04, 0.10)	21.07 (−55.67, 201.64)	−0.04 (−0.28, 0.20)
Southeast Asia	40.64 (13.03, 73.48)	0.02 (0.01, 0.04)	60.30 (28.46, 104.91)	0.03 (0.02, 0.06)	48.40 (−28.40, 179.85)	0.66 (0.48, 0.84)
Southern Latin America	22.63 (14.74, 34.62)	0.15 (0.10, 0.23)	35.61 (22.51, 49.37)	0.25 (0.16, 0.34)	57.34 (−10.88, 143.63)	1.81 (1.53, 2.09)
Southern Sub-Saharan Africa	12.36 (7.16, 18.50)	0.06 (0.03, 0.09)	32.09 (15.81, 47.42)	0.13 (0.07, 0.20)	159.56 (57.70, 321.51)	3.25 (2.76, 3.75)
Tropical Latin America	64.10 (45.30, 94.26)	0.12 (0.08, 0.18)	84.09 (42.38, 119.04)	0.17 (0.08, 0.24)	31.20 (−38.51, 85.63)	1.42 (0.82, 2.02)
Western Europe	98.66 (66.89, 159.56)	0.14 (0.09, 0.22)	161.10 (66.76, 252.54)	0.24 (0.10, 0.37)	63.29 (−39.90, 185.95)	1.87 (1.20, 2.54)
Western Sub-Saharan Africa	642.91 (300.83, 939.29)	0.73 (0.34, 1.07)	1324.58 (746.10, 1805.51)	0.62 (0.35, 0.84)	106.03 (55.11, 193.31)	−0.37 (−0.47, −0.26)

EAPC, estimated annual percentage change; SDI, Sociodemographic Index; UI, uncertainty interval.

a EAPC is expressed as 95% confidence interval.

#### Mortality

The trend in BL-related mortality mirrored that of incidence, showing an initial rise followed by a decline. The peak mortality rate occurred in 2016, at 0.17 per 100,000 (95% UI, 0.11–0.21) (Figure 1B). From 1990 to 2021, the global number of BL-related deaths increased by 24.7%, from 2,457.9 (95% UI, 1,298.8–3,588.5) to 3,065.5 (95% UI, 1,928.3–3,971.3), with an EAPC of 0.44 (95% CI, 0.32–0.56) (Table 2). Age-group variations in mortality were observed, with the largest increase in children aged 10–14 (36.8%) and the largest decrease in 1–2-year-olds (17.5%) (Figure 2B). In 1990, the highest mortality rate was seen in 2–4-year-olds, representing 35.3% of all BL-related deaths. By 2021, this shifted to the 5–9-year-old group, which accounted for 32.8% of deaths (Figure 3B). A gender disparity in mortality was evident, with higher mortality rates in boys compared to girls, particularly in the 2–4-year age group (Figure 2B).

**Table 2:** Mortality of Burkitt lymphoma in children between 1990 and 2021 at the global and regional level.

Location	1990		2021		1990–2021	
	Death cases	Mortality	Death cases	Mortality	Cases change	EAPC^a^
Global	2457.85 (1298.83, 3588.50)	0.14 (0.07, 0.21)	3065.54 (1928.32, 3971.27)	0.15 (0.10, 0.20)	24.72 (−6.85, 77.71)	0.44 (0.32, 0.56)
SDI						
High SDI	52.87 (39.04, 73.67)	0.03 (0.02, 0.04)	34.23 (17.96, 45.05)	0.02 (0.01, 0.03)	−35.27 (−67.82, −17.87)	−1.11 (−1.42, −0.79)
High-middle SDI	155.07 (92.51, 219.91)	0.06 (0.03, 0.08)	62.76 (41.27, 79.71)	0.03 (0.02, 0.03)	−59.53 (−75.97, −35.11)	−2.59 (−2.81, −2.37)
Middle SDI	310.77 (193.56, 406.69)	0.05 (0.03, 0.07)	237.35 (141.40, 304.28)	0.04 (0.02, 0.05)	−23.62 (−54.81, 11.41)	−0.75 (−0.94, −0.55)
Low-middle SDI	563.36 (288.97, 820.67)	0.12 (0.06, 0.17)	735.48 (489.16, 961.17)	0.13 (0.08, 0.17)	30.55 (−11.82, 95.54)	0.26 (0.16, 0.35)
Low SDI	1374.34 (644.93, 2170.95)	0.60 (0.28, 0.95)	1994.05 (1160.86, 2668.28)	0.43 (0.25, 0.58)	45.09 (4.87, 119.75)	−0.89 (−0.97, −0.81)
Regions						
Andean Latin America	17.64 (11.23, 28.75)	0.12 (0.08, 0.19)	13.12 (7.45, 19.43)	0.07 (0.04, 0.11)	−25.67 (−69.46, 38.10)	−1.83 (−2.02, −1.65)
Australasia	1.30 (0.89, 1.98)	0.03 (0.02, 0.04)	0.99 (0.49, 1.46)	0.02 (0.01, 0.03)	−24.31 (−65.00, 20.95)	−1.84 (−2.42, −1.25)
Caribbean	15.07 (8.41, 31.65)	0.13 (0.07, 0.28)	12.33 (5.22, 25.91)	0.11 (0.05, 0.23)	−18.17 (−57.35, 59.71)	0.09 (−0.17, 0.36)
Central Asia	5.12 (3.21, 8.59)	0.02 (0.01, 0.03)	2.95 (1.77, 4.66)	0.01 (0.01, 0.02)	−42.47 (−71.41, 2.03)	−2.34 (−2.70, −1.98)
Central Europe	7.43 (4.71, 14.02)	0.03 (0.02, 0.05)	3.53 (1.67, 5.07)	0.02 (0.01, 0.03)	−52.55 (−85.09, −22.16)	−0.68 (−1.17, −0.19)
Central Latin America	49.63 (35.69, 76.25)	0.08 (0.06, 0.12)	40.44 (23.14, 54.46)	0.06 (0.04, 0.09)	−18.50 (−59.98, 17.54)	−0.41 (−0.62, −0.19)
Central Sub-Saharan Africa	132.57 (47.36, 232.94)	0.52 (0.19, 0.92)	139.09 (69.03, 204.21)	0.24 (0.12, 0.35)	4.92 (−29.93, 134.86)	−2.28 (−2.47, −2.09)
East Asia	134.68 (57.89, 202.02)	0.04 (0.02, 0.06)	31.83 (17.36, 53.63)	0.01 (0.01, 0.02)	−76.37 (−89.09, −39.32)	−5.33 (−5.87, −4.79)
Eastern Europe	25.44 (15.25, 37.77)	0.05 (0.03, 0.07)	8.83 (3.76, 12.39)	0.02 (0.01, 0.03)	−65.29 (−86.02, −49.99)	−1.88 (−2.16, −1.61)
Eastern Sub-Saharan Africa	877.38 (399.91, 1354.12)	0.97 (0.44, 1.50)	1115.92 (642.59, 1535.69)	0.63 (0.36, 0.86)	27.19 (−10.45, 106.73)	−1.28 (−1.36, −1.20)
High-income Asia Pacific	6.62 (3.86, 10.06)	0.02 (0.01, 0.03)	3.35 (1.56, 4.50)	0.01 (0.01, 0.02)	−49.43 (−79.29, −0.09)	−0.78 (−1.12, −0.44)
High-income North America	21.05 (14.79, 28.71)	0.03 (0.02, 0.05)	14.46 (9.11, 19.67)	0.02 (0.01, 0.03)	−31.31 (−56.90, −9.65)	−1.18 (−1.46, −0.91)
North Africa and Middle East	108.66 (61.88, 177.49)	0.08 (0.04, 0.13)	75.12 (47.84, 129.82)	0.04 (0.03, 0.07)	−30.87 (−65.21, 28.91)	−2.14 (−2.37, −1.92)
Oceania	0.56 (0.22, 1.19)	0.02 (0.01, 0.04)	1.96 (0.57, 4.22)	0.04 (0.01, 0.08)	249.32 (54.86, 661.53)	2.10 (1.74, 2.47)
South Asia	273.67 (97.95, 469.86)	0.06 (0.02, 0.11)	276.00 (150.18, 429.62)	0.05 (0.03, 0.08)	0.85 (−63.76, 131.91)	−0.67 (−0.82, −0.51)
Southeast Asia	36.99 (12.24, 66.14)	0.02 (0.01, 0.04)	32.36 (17.19, 48.49)	0.02 (0.01, 0.03)	−12.54 (−52.46, 71.54)	−1.08 (−1.31, −0.84)
Southern Latin America	13.67 (9.41, 19.56)	0.09 (0.06, 0.13)	10.14 (6.47, 13.91)	0.07 (0.04, 0.10)	−25.82 (−56.79, 13.95)	−0.61 (−0.76, −0.46)
Southern Sub-Saharan Africa	11.97 (6.92, 18.11)	0.06 (0.03, 0.09)	27.91 (13.64, 42.06)	0.12 (0.06, 0.17)	133.12 (36.67, 286.88)	2.92 (2.48, 3.36)
Tropical Latin America	57.62 (40.85, 82.62)	0.11 (0.08, 0.15)	37.51 (20.44, 51.57)	0.07 (0.04, 0.10)	−34.90 (−68.93, −8.25)	−0.87 (−1.42, −0.31)
Western Europe	19.27 (13.54, 30.39)	0.03 (0.02, 0.04)	14.54 (6.27, 20.84)	0.02 (0.01, 0.03)	−24.55 (−71.72, 22.38)	−0.70 (−1.14, −0.26)
Western Sub-Saharan Africa	641.50 (295.56, 936.13)	0.73 (0.34, 1.07)	1203.19 (664.27, 1615.84)	0.56 (0.31, 0.75)	87.56 (44.84, 167.52)	−0.65 (−0.79, −0.51)

EAPC, estimated annual percentage change; SDI, Sociodemographic Index; UI, uncertainty interval.

a EAPC is expressed as 95% confidence interval.

#### DALYs

Trends in DALYs followed those of incidence and mortality, with an initial increase followed by a decline. The peak DALY rate was observed in 2016 at 14.21 per 100,000 (95% UI, 8.90–17.87) (Figure 1C). From 1990 to 2021, the global DALYs associated with childhood BL increased by 23.5%, from 207,119.99 (95% UI, 109,264.23–303,696.33) to 255,842.92 (95% UI, 160,376.28–331,628.88), with an EAPC of 0.42 (95% CI, 0.29–0.54) (Supplementary Table S1). The largest increase in DALYs was seen in children aged 10–14 years (36.9%), while the largest decrease occurred in 1–2-year-olds (−17.4%) (Figure 2C). In 1990, the highest DALY rate was seen in 2–4-year-olds, accounting for 36.1% of total DALYs. By 2021, the highest DALY rate shifted to the 5–9-year-old group, which accounted for 32.3% of total DALYs (Figure 3C).

### Regional trends by SDI

**Figure 4:**
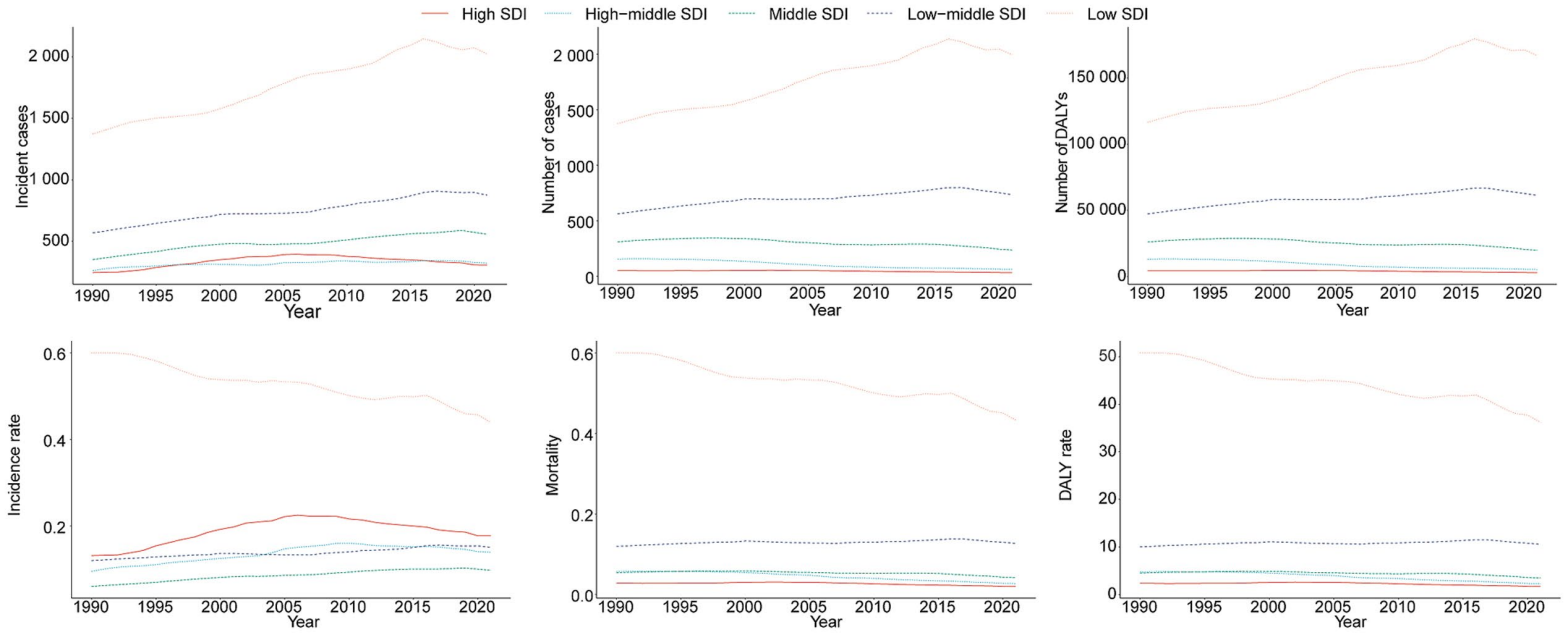
Epidemiologic trends in childhood Burkitt lymphoma incidence, mortality, and disability-adjusted life years (DALYs) rates across five Sociodemographic Index (SDI) areas from 1990 to 2021.

In 2021, the low SDI region reported the highest number of incident cases (2,021.9, 95% UI, 1,195.7–2,717.9) and the highest incidence rate (0.44 per 100,000, 95% UI, 0.26–0.59), with an EAPC of −0.86 (95% CI, −0.94 to −0.79). This region also showed the highest BL-related mortality and DALY rates, at 0.43 per 100,000 (95% UI, 0.25–0.58) and 36.19 (95% UI, 21.00–48.64), respectively, with EAPCs of −0.89 (95% CI, −0.97 to −0.81) and −0.93 (95% CI, −1.01 to −0.85) (Tables 1, 2; Supplementary Table S1 and Figure 4).

### National trends

#### Incidence

**Figure 5:**
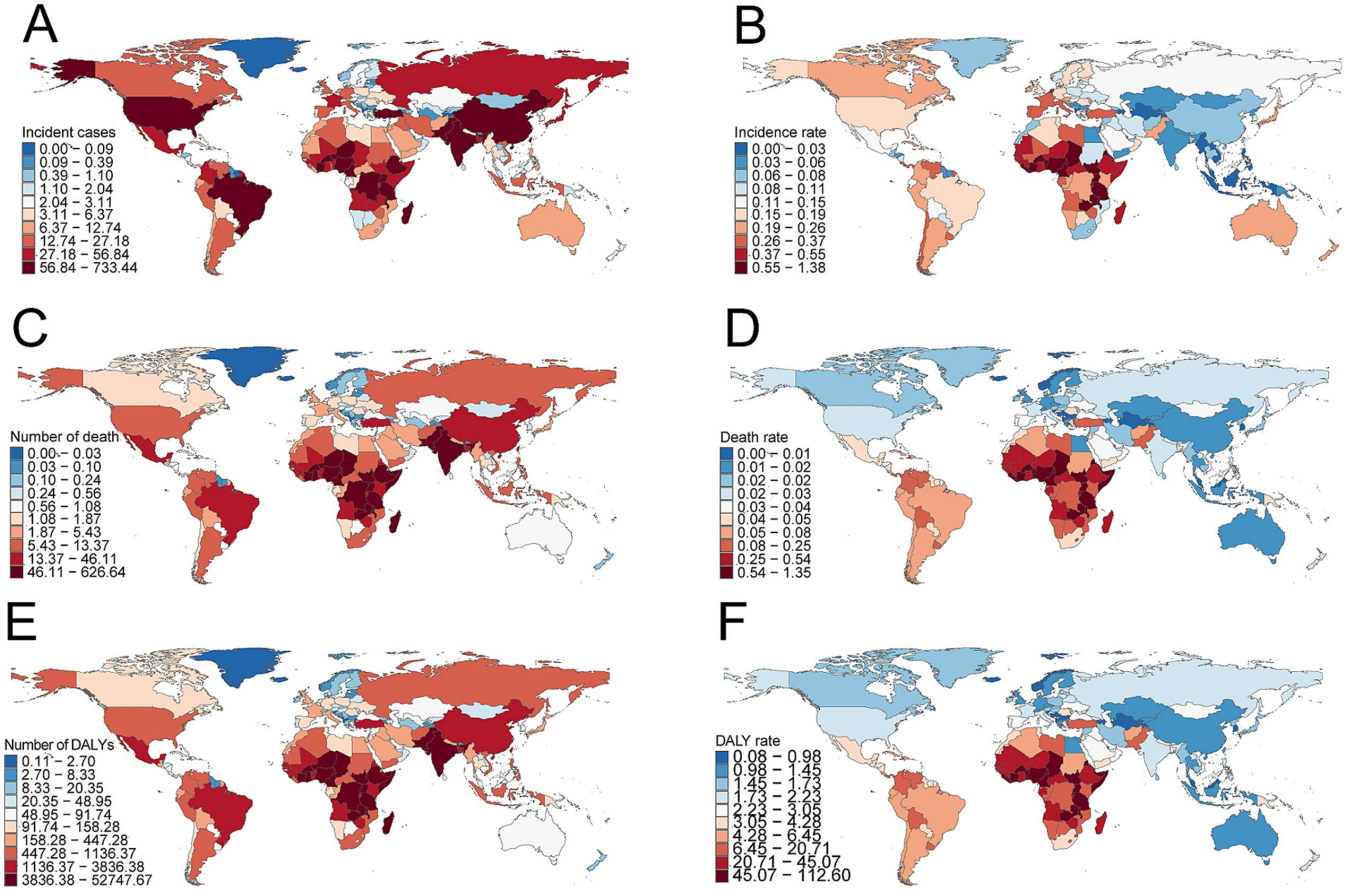
Incidence, deaths, and disability-adjusted life years (DALYs) of childhood Burkitt’s lymphoma across 204 countries and territories. **(A)** Number of incident cases. **(B)** Incidence rate. **(C)** Number of deaths. **(D)** Death rate. **(E)** Number of DALYs. **(F)** DALY rate.

In 2021, Nigeria had the highest total number of BL cases globally (726.2, 95% UI, 367.2–1,029.4), while Uganda had the highest incidence rate (1.36 per 100,000, 95% UI, 0.77–2.15) (Supplementary Table S2 and Figures 5A,B). From 1990 to 2021, Guam showed the highest increase in incidence (EAPC = 5.83, 95% CI, 4.76–6.91), while Ghana saw the largest decrease (EAPC = −4.04, 95% CI, −4.77 to −3.32) (Supplementary Table S2).

#### Mortality

Nigeria reported the highest number of BL-related deaths globally in 2021 (620.4, 95% UI, 312.2–882.2), while Uganda had the highest mortality rate (1.34 per 100,000, 95% UI, 0.77–2.05) (Supplementary Table S3 and Figures 5C,D). From 1990 to 2021, Guam experienced the largest increase in mortality (EAPC = 5.00, 95% CI, 4.13–5.88), while China showed the largest decrease (EAPC = −5.47, 95% CI, −6.03 to −4.92) (Supplementary Table S3).

#### DALYs

Nigeria had the highest total DALYs related to childhood BL in 2021 (52,225.4, 95% UI, 26,224.0–74,292.6), while Uganda had the highest DALY rate (111.5 per 100,000, 95% UI, 63.5–171.9) (Supplementary Table S3 and Figures 5D–F). From 1990 to 2021, Guam showed the largest increase in DALY rate (EAPC = 5.01, 95% CI, 4.17–5.86), while China had the largest decrease (EAPC = −5.41, 95% CI, −5.95 to −4.87) (Supplementary Table S4).

### Projections for 2035

**Figure 6:**
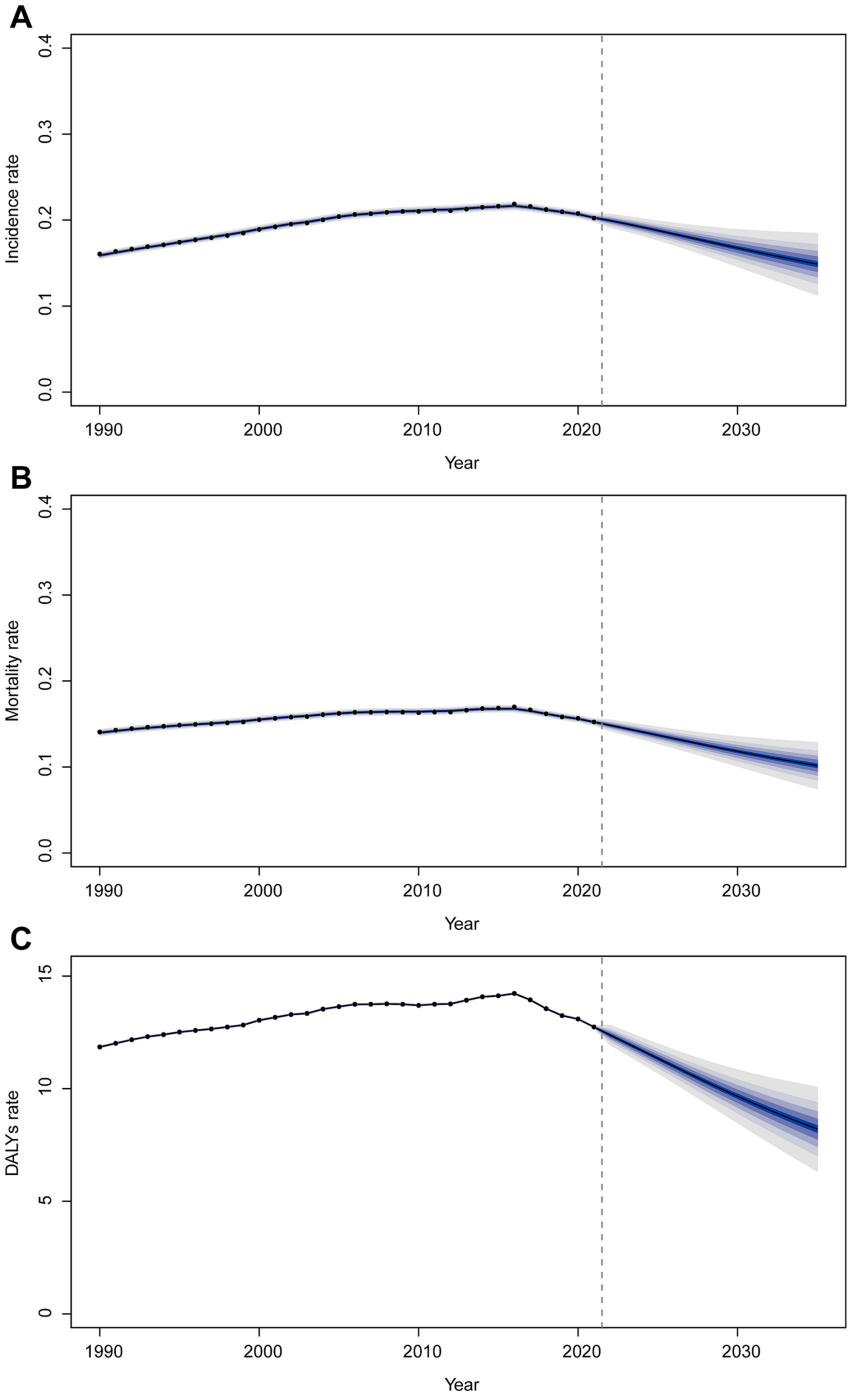
Trends of incidence rate **(A)**, mortality **(B)**, and DALY rate **(C)** from 2021 to 2035 predicted by BAPC models.

Using BAPC modeling, the projected trends for childhood BL incidence, mortality, and DALY rates from 2021 to 2035 suggest a continued decline. By 2035, the incidence rate is expected to reach 0.15 (95% CI 0.11–0.19) per 100,000 (Figure 6A), mortality to 0.10 (95% CI 0.07–0.13) per 100,000 (Figure 6B), and DALY rate to 8.21 (95% CI 5.98–10.43) per 100,000 (Figure 6C).

## Discussion

Our analysis provides a comprehensive overview of the global burden of pediatric BL, untangling profound epidemiological patterns and projecting future trends. Overall, we observed that over recent decades, the incidence rate and mortality of pediatric BL have exhibited distinct patterns across different regions and socioeconomic settings. While high-income regions demonstrated increased incidence rates (likely in response to both genuine rises in sporadic BL and improved diagnostics), some low-SDI regions showed stable or even declining rates. Despite these regional variations, BL remains predominantly a disease of childhood and adolescence, with persistent male predominance.

We found that BL incidence rates and mortality were consistently higher in males (aged 2–14 years) than in females. This gender disparity, well-documented in the literature, may stem from genetic or immunological factors, though a definitive explanation remains elusive (22, 23). Importantly, our results confirm that the marked predilection of pediatric BL for male patients is a global phenomenon, underscoring the need for further investigation into sex-related determinants of BL susceptibility.

Another notable epidemiological characteristic of BL is its distinctive age distribution. In endemic regions, BL almost exclusively affects young children (24, 25), a pattern consistently observed in our findings: a sharp incidence peak occurs during childhood (particularly between 5 and 9 years of age). These age-specific trends are intrinsically linked to the pathophysiology of BL. The early childhood peak is most pronounced in equatorial Africa, temporally coinciding with exposure to two key cofactors: EBV infection and early-life malaria exposure (26). Endemic BL develops in regions with holoendemic malaria, where primary EBV infection occurs during infancy. Notably, in certain Ugandan case series, the median age at BL diagnosis is approximately 7 years, with few cases reported after adolescence (27). In these settings, young children experience chronic immune stimulation from plasmodium falciparum malaria while frequently acquiring EBV infection within the first years of life (28). This synergistic combination drives BL tumorigenesis. EBV plays a pivotal role by infecting B cells and promoting their proliferation, whereas concomitant malaria infection both elevates EBV viremia and impairs EBV-specific immune surveillance. These mechanisms collectively create permissive conditions for the uncontrolled proliferation of B cells harboring MYC translocations (29, 30). Consequently, the age-specific incidence rate of BL (peaking in childhood) closely aligns with the disease’s underlying biology: early-life exposures (EBV and malaria) drive the pediatric endemic form.

Geographically, our study underscores the enormous disparity in BL burden between world regions. Historically, the highest incidence of BL has been in equatorial Africa—the classic “lymphoma belt” described by Burkitt, where BL accounts for up to 30–50% of all childhood cancers (31). We found that countries like Uganda and Nigeria still have among the greatest BL incidence and mortality rates. In 2021, Uganda had the world’s highest estimated BL incidence rate (around 1.36 per 100,000 in children). These figures reflect the enduring impact of endemic cofactors in these regions. Early EBV infection is nearly universal in such settings; for example, by age 3–4 years, the vast majority of children in rural East Africa are EBV-seropositive, whereas in high-income countries, EBV infection is often delayed until adolescence (32). In resource-limited communities, overcrowded living conditions, poor sanitation, and increased exposure to infectious agents (e.g., EBV and malaria) collectively contribute to this early infection cycle (33). Furthermore, inadequate healthcare infrastructure results in minimal preventive measures and early detection efforts. Consequently, children in impoverished malaria-endemic regions face a “perfect storm” of BL risk factors—a situation further compounded by socioeconomic barriers. It should be noted, however, that while malnutrition frequently coexists with poverty, no definitive evidence has established it as a direct causative factor for BL. Although malnutrition may impair general immune function, current data lack direct evidence linking it to an increased occurrence rate of BL. Instead, the high BL incidence in endemic regions can be more plausibly explained by the infection-driven model (EBV + malaria) and delayed diagnosis (34). Socioeconomic deprivation indirectly contributes by enhancing exposure to these infections and impeding access to healthcare, rather than through malnutrition per se.

It is encouraging to note that our discussion must also emphasize that improvements in infectious disease control may alter the incidence rate of BL (35, 36). A notable example is the impact of malaria control on endemic BL. Several studies have demonstrated that reducing the burden of malaria leads to a subsequent decline in BL cases. A recent systematic review and meta-analysis found that the large-scale rollout of insecticide-treated bed nets in sub-Saharan Africa during the 2000s was associated with a significant ~44% decline in childhood BL incidence in the years following the introduction of bed nets (37). Specifically, the pooled BL incidence rate dropped from approximately 1.36 per 100,000 before bed net programs to 0.76 per 100,000 after their implementation. Moreover, each 1% increase in regional bed net usage was linked to an estimated 2% reduction in BL incidence risk. These data strongly support the notion that aggressive malaria control measures (such as mosquito bed nets, indoor residual spraying, and anti-malarial treatments) confer an added benefit of preventing EBV—malaria co-infection in children, lowering endemic BL incidence. Going forward, sustaining and expanding malaria control in Africa could further reduce the BL burden, a critical example of how public health interventions against infections can influence cancer epidemiology. In parallel, efforts to develop an EBV vaccine are underway; a successful EBV vaccine could also profoundly affect BL incidence in the long term, especially in endemic areas.

While incidence patterns reveal where BL cases are occurring, an equally important part of the story is outcomes, how survival and mortality differ across settings. Here, our findings and the broader literature paint a picture of stark contrasts between high-income countries (HICs) and low- and middle-income countries (LMICs). BL is often cited as a “curable” pediatric cancer, and this is true in HICs today. With prompt diagnosis and intensive combination chemotherapy, long-term survival for children with BL in high-resource settings now routinely exceeds 85–90%. Recent clinical trials have achieved cure rates approaching or above 90% for pediatric BL. For instance, the international Inter-B-NHL Ritux 2010 trial (COG ANHL1131) demonstrated a 3-year event-free survival of 93.9% in children and adolescents with high-risk BL who received rituximab plus intensive chemotherapy, compared to 82.3% EFS with chemotherapy alone (38). As a result of such advances, the expected survival for a child with BL in a developed country is outstanding: even advanced-stage BL now has a reported >95% survival rate with first-line therapy (39). This represents a remarkable improvement from historical outcomes, a testament to optimized chemotherapy regimens (e.g., the LMB protocol), the incorporation of immunotherapy (rituximab), and superior supportive care. However, the situation is very different in many low-resource regions. In sub-Saharan Africa, where the majority of BL cases occur, outcomes remain poor. Most children present with bulky, advanced disease, and the healthcare facilities often cannot deliver the kind of intensive therapy used in HICs (due to resource constraints and higher treatment-related toxicity without adequate supportive care). Consequently, reported survival rates for endemic BL in Africa have lagged far behind (40, 41). Long-term survival is often in the range of only ~30–50% in many centers in equatorial Africa, a figure that tragically has changed little since the 1970s (42). Even with some treatment available, a large proportion of children succumb to the disease; BL continues to cause high childhood mortality in those countries. Efforts by regional oncology groups and international collaborators have shown that outcomes can be improved, for example, modified, lower-intensity regimens tailored to local contexts have pushed some survival rates above 60–70% in select African hospitals (43, 44). In summary, BL in high-income settings is now a highly curable malignancy, whereas in its endemic heartland, it still often carries a poor prognosis. This dichotomy highlights the urgent need for global health initiatives to improve cancer care infrastructure in low-income countries. Expanding successful treatments (potentially via twinning programs, clinical trials in Africa, and adoption of supportive care protocols) could dramatically reduce global BL mortality.

Finally, the BAPC model was employed to project the burden of childhood BL through 2035. Notably, our model predicted a declining trend in both the incidence and mortality rates of BL over the next decade. This projected improvement is driven largely by anticipated gains in certain regions, for example, continued reductions in BL in low-SDI countries (owing to better infection control and slowly improving healthcare access) and stable or modest declines in high-SDI countries (where incidence may plateau). These findings are cautiously optimistic. However, we recognize that they differ from the more conservative projections of the WHO’s Global Cancer Observatory (GCO) and other models. The GCO’s methodology typically projects cancer burden by holding current incidence rates constant and extrapolating based on population growth and demographic shifts (45). Our model, in contrast, attempts to account for epidemiologic changes—essentially incorporating the recent downtrends seen in some areas into the forecast. The discrepancy can be illustrated by analogy: for Hodgkin lymphoma, GCO projections assume rates unchanged and foresee ~30% more cases by 2040 due to demographics, whereas if underlying risk factors were changing, we would get a different outlook. In the case of BL, our BAPC-based prediction of improving rates implies that interventions (like malaria control, HIV control, and better treatment) will start to pay dividends by reducing incidence and mortality rates, not just case-fatality. We believe this is a plausible scenario given the evidence of declining BL in some historically high-burden areas (e.g., parts of East Africa with successful malaria reduction programs) (37). It is also consistent with the idea that global childhood cancer outcomes are slowly improving with increased awareness and access to treatment.

In conclusion, the global burden of BL reflects a convergence of infectious disease epidemiology, socio-economic disparities, and healthcare system capabilities. Males and young children bear the brunt of BL incidence, especially in places where EBV and malaria are entrenched. Advances in therapy have made BL highly curable in the developed world, yet the same disease still exacts an unacceptably high toll in developing countries. Going forward, a greater focus on preventive strategies (such as malaria control and potentially EBV vaccination) and health system strengthening in endemic regions is warranted. Our projections offer hope that BL incidence and mortality could decline in the coming years, but realizing this potential will require addressing the very global inequities that our study has illuminated. By improving childhood cancer care in low-income settings and continuing to target the infectious roots of BL, we can work to ensure that the optimistic scenario envisioned by our model becomes a reality, one in which a child’s chance of surviving BL no longer depends on the lottery of birthplace.

### Limitations

This study has several limitations. First, it relies heavily on GBD data, whose accuracy depends on the availability and quality of national registries. Underdiagnosis of childhood BL and missing data on relevant risk factors may limit completeness. Second, as a GBD-based cross-sectional analysis, variability in case definitions and reporting standards across countries may lead to the underestimation of BL incidence, particularly in low-resource settings. Finally, the GBD database does not provide detailed risk stratification among BL subtypes, which limits the ability to conduct subgroup analyses.

## Conclusion

Overall, childhood BL remains a significant global public health challenge, with its burden continuing to increase in certain regions, necessitating sustained attention and effective interventions. Comprehensive and targeted prevention strategies are crucial, including public health advocacy, improved screening programs, and effective management of risk factors, particularly among high-risk populations. Future research efforts should focus on enhancing early detection methods through advances in genetic and molecular epidemiology, as well as investigating the role of environmental and occupational exposures in disease development. Additionally, the development and evaluation of an EBV vaccine hold promise as a preventive measure. Enhancing predictive capabilities through more detailed data collection and analysis will further strengthen our understanding of global trends, enabling more precise interventions. Longitudinal studies are essential for assessing the real impact of diagnostic and therapeutic advancements on disease burden and guiding evidence-based policies to mitigate the long-term effects of childhood BL.

## Data Availability

The datasets presented in this study can be found in online repositories. The names of the repository/repositories and accession number(s) can be found in the article/Supplementary material.

## Footnotes

^1^ https://vizhub.healthdata.org/gbd-results/
