# Supplementary material for: Global burden of childhood Burkitt lymphoma (1990–2021): epidemiological trends, regional disparities, and projections for 2035 from the Global Burden of Disease Study
Source: Front Med (Lausanne). 2025 Sep 24;12:1619750. doi: 10.3389/fmed.2025.1619750 (PMC12504260; doi:10.3389/fmed.2025.1619750)
Supplement: Supplementary file 1 [file Table_1.DOCX]

Table S1. DALYs of Burkitt lymphoma in children between 1990 and 2021 at the global and regional level.

|  | 1990 |  |  | 2021 |  |  | 1990-2021 |  |
| --- | --- | --- | --- | --- | --- | --- | --- | --- |
| location | DALYs Cases | DALY rate |  | DALYs Cases | DALY rate |  | Cases change | EAPC ^a^ |
| Global | 207119.99(109264.23,303696.33) | 11.91(6.28,17.46) |  | 255842.92(160376.28,331628.88) | 12.72(7.97,16.48) |  | 23.52(-7.81,76.68) | 0.42(0.29,0.54) |
| **SDI**  High SDI | 4432.04(3265.13,6178.35) | 2.39(1.76,3.33) |  | 2931.93(1523.75,3826.60) | 1.70(0.88,2.22) |  | -33.85(-67.15,-15.63) | -1.03(-1.34,-0.71) |
| High-middle SDI | 12926.83(7717.51,18362.44) | 4.72(2.82,6.71) |  | 5284.47(3481.72,6676.55) | 2.29(1.51,2.89) |  | -59.12(-75.79,-34.17) | -2.55(-2.76,-2.33) |
| Middle SDI | 26002.86(16152.22,34068.91) | 4.50(2.80,5.90) |  | 19797.88(11804.23,25429.53) | 3.49(2.08,4.49) |  | -23.86(-55.01,11.36) | -0.75(-0.94,-0.55) |
| Low-middle SDI | 47294.16(24177.46,69132.50) | 10.02(5.12,14.64) |  | 61131.89(40488.32,80335.90) | 10.54(6.98,13.85) |  | 29.26(-12.96,94.20) | 0.23(0.14,0.33) |
| Low SDI | 116342.67(54199.88,183966.18) | 50.82(23.68,80.36) |  | 166557.29(96664.73,223873.39) | 36.19(21.00,48.64) |  | 43.16(3.11,117.25) | -0.93(-1.01,-0.85) |
| **Regions** |  |  |  |  |  |  |  |  |
| Andean Latin America | 1469.07(935.36,2395.83) | 9.89(6.30,16.13) |  | 1092.12(621.09,1629.11) | 6.04(3.43,9.00) |  | -25.66(-69.60,39.59) | -1.83(-2.01,-1.64) |
| Australasia | 109.94(74.50,166.06) | 2.40(1.62,3.62) |  | 85.38(42.37,126.54) | 1.49(0.74,2.21) |  | -22.35(-64.40,24.93) | -1.73(-2.33,-1.14) |
| Caribbean | 1269.33(696.64,2705.44) | 11.12(6.10,23.71) |  | 1034.06(432.33,2202.43) | 8.99(3.76,19.14) |  | -18.53(-57.75,61.47) | 0.08(-0.19,0.35) |
| Central Asia | 430.55(271.36,721.41) | 1.72(1.09,2.89) |  | 247.10(148.01,392.19) | 0.89(0.53,1.42) |  | -42.61(-71.71,0.90) | -2.32(-2.70,-1.95) |
| Central Europe | 616.63(390.72,1163.78) | 2.09(1.33,3.95) |  | 297.78(141.32,427.47) | 1.68(0.80,2.41) |  | -51.71(-84.85,-20.99) | -0.61(-1.09,-0.12) |
| Central Latin America | 4148.42(2978.30,6387.31) | 6.44(4.63,9.92) |  | 3371.49(1922.46,4539.83) | 5.31(3.03,7.15) |  | -18.73(-60.24,17.05) | -0.41(-0.62,-0.19) |
| Central Sub-Saharan Africa | 11315.44(3980.44,19995.65) | 44.73(15.73,79.04) |  | 11613.28(5684.84,17077.51) | 19.79(9.69,29.10) |  | 2.63(-31.60,132.32) | -2.34(-2.54,-2.15) |
| East Asia | 11381.77(4833.02,17086.39) | 3.45(1.47,5.18) |  | 2724.16(1479.02,4597.77) | 1.02(0.55,1.72) |  | -76.07(-89.09,-38.15) | -5.27(-5.79,-4.75) |
| Eastern Europe | 2120.79(1270.27,3148.90) | 4.12(2.47,6.12) |  | 738.70(314.02,1035.24) | 2.08(0.89,2.92) |  | -65.17(-86.04,-49.68) | -1.83(-2.12,-1.54) |
| Eastern Sub-Saharan Africa | 74079.74(33511.01,114746.35) | 81.79(37.00,126.69) |  | 92764.43(53330.23,127944.01) | 51.99(29.89,71.70) |  | 25.22(-12.14,103.78) | -1.33(-1.41,-1.24) |
| High-income Asia Pacific | 552.17(321.36,841.35) | 1.57(0.91,2.39) |  | 289.53(133.50,388.50) | 1.29(0.60,1.73) |  | -47.56(-78.62,3.70) | -0.65(-1.00,-0.30) |
| High-income North America | 1769.48(1244.30,2408.14) | 2.87(2.02,3.90) |  | 1230.99(776.64,1667.83) | 1.88(1.18,2.54) |  | -30.43(-56.57,-8.49) | -1.14(-1.41,-0.86) |
| North Africa and Middle East | 8975.10(5075.90,14778.65) | 6.39(3.61,10.52) |  | 6225.82(3975.50,10779.24) | 3.40(2.17,5.88) |  | -30.63(-65.18,29.10) | -2.12(-2.35,-1.90) |
| Oceania | 47.63(18.63,102.55) | 1.78(0.70,3.83) |  | 166.05(48.20,360.91) | 3.27(0.95,7.10) |  | 248.61(55.95,660.38) | 2.10(1.74,2.47) |
| South Asia | 22785.65(8090.22,39252.33) | 5.26(1.87,9.06) |  | 22813.34(12382.61,35931.33) | 4.50(2.44,7.09) |  | 0.12(-64.41,131.29) | -0.68(-0.84,-0.52) |
| Southeast Asia | 3092.26(1007.53,5560.87) | 1.81(0.59,3.26) |  | 2690.48(1415.11,4030.98) | 1.56(0.82,2.33) |  | -12.99(-53.04,72.01) | -1.09(-1.32,-0.85) |
| Southern Latin America | 1135.94(785.88,1628.91) | 7.61(5.27,10.91) |  | 847.81(538.57,1159.03) | 5.85(3.72,8.00) |  | -25.37(-56.54,15.16) | -0.58(-0.73,-0.43) |
| Southern Sub-Saharan Africa | 998.85(572.48,1509.77) | 4.83(2.77,7.30) |  | 2311.47(1128.48,3471.44) | 9.60(4.69,14.42) |  | 131.41(36.74,281.31) | 2.93(2.47,3.38) |
| Tropical Latin America | 4807.89(3404.96,6912.44) | 8.97(6.35,12.89) |  | 3113.84(1693.09,4280.07) | 6.20(3.37,8.53) |  | -35.23(-69.17,-8.36) | -0.88(-1.43,-0.32) |
| Western Europe | 1617.40(1147.53,2528.96) | 2.28(1.62,3.56) |  | 1262.29(542.80,1823.38) | 1.85(0.80,2.68) |  | -21.96(-70.95,26.66) | -0.58(-1.02,-0.13) |
| Western Sub-Saharan Africa | 54395.92(25003.33,79833.09) | 61.90(28.45,90.84) |  | 100922.80(55474.84,135573.42) | 46.99(25.83,63.13) |  | 85.53(43.07,165.15) | -0.68(-0.82,-0.54) |
