# Supplementary material for: Global burden of childhood Burkitt lymphoma (1990–2021): epidemiological trends, regional disparities, and projections for 2035 from the Global Burden of Disease Study
Source: Front Med (Lausanne). 2025 Sep 24;12:1619750. doi: 10.3389/fmed.2025.1619750 (PMC12504260; doi:10.3389/fmed.2025.1619750)
Supplement: Supplementary file 2 [file Table_2.DOCX]

Table S2. Incidence of Burkitt lymphoma in children between 1990 and 2021 at the national level.

| location | 1990 | |  | 2021 | |  | 1990-2021 | |
| --- | --- | --- | --- | --- | --- | --- | --- | --- |
|  | Incident cases | Incidence rate |  | Incident cases | Incidence rate |  | Cases change | EAPC |
| Afghanistan | 4.38(1.16,13.04) | 0.10(0.03,0.30) |  | 8.82(2.71,23.56) | 0.06(0.02,0.17) |  | 101.36(-38.62,426.90) | -1.004728009 |
| Albania | 0.05(0.02,0.11) | 0.00(0.00,0.01) |  | 0.09(0.02,0.23) | 0.02(0.00,0.05) |  | 79.17(-67.44,521.54) | 5.444862819 |
| Algeria | 10.80(2.93,22.95) | 0.10(0.03,0.21) |  | 23.58(8.48,50.81) | 0.18(0.06,0.38) |  | 118.35(-11.89,492.63) | 2.004017897 |
| American Samoa | 0.00(0.00,0.00) | 0.01(0.00,0.02) |  | 0.00(0.00,0.01) | 0.03(0.01,0.07) |  | 147.82(-24.98,726.79) | 4.022017993 |
| Andorra | 0.04(0.01,0.07) | 0.38(0.14,0.75) |  | 0.05(0.02,0.10) | 0.50(0.21,0.95) |  | 42.07(-46.50,341.11) | 0.97302918 |
| Angola | 32.22(9.26,61.35) | 0.68(0.20,1.30) |  | 41.36(19.16,66.23) | 0.27(0.13,0.43) |  | 28.37(-25.99,292.53) | -2.922326085 |
| Antigua and Barbuda | 0.02(0.01,0.03) | 0.09(0.05,0.14) |  | 0.02(0.01,0.04) | 0.15(0.09,0.22) |  | 52.25(-1.03,207.07) | 1.44780114 |
| Argentina | 15.15(9.08,24.07) | 0.15(0.09,0.24) |  | 23.39(14.52,32.87) | 0.23(0.14,0.32) |  | 54.38(-6.37,141.06) | 1.56497736 |
| Armenia | 0.26(0.12,0.54) | 0.02(0.01,0.05) |  | 0.21(0.11,0.37) | 0.04(0.02,0.06) |  | -18.86(-68.91,81.83) | 2.179110623 |
| Australia | 6.22(3.81,9.63) | 0.16(0.10,0.25) |  | 9.18(4.09,15.47) | 0.19(0.09,0.33) |  | 47.78(-38.62,165.43) | 0.487317098 |
| Austria | 1.94(1.22,3.06) | 0.14(0.09,0.23) |  | 2.89(1.41,4.58) | 0.22(0.11,0.35) |  | 49.19(-31.03,158.38) | 1.378349061 |
| Azerbaijan | 0.42(0.15,0.93) | 0.02(0.01,0.04) |  | 0.63(0.27,1.31) | 0.03(0.01,0.06) |  | 49.65(-41.97,306.58) | 0.971668957 |
| Bahamas | 0.09(0.06,0.14) | 0.11(0.07,0.17) |  | 0.12(0.07,0.19) | 0.15(0.08,0.23) |  | 32.27(-20.61,100.33) | 0.618487789 |
| Bahrain | 0.05(0.02,0.11) | 0.03(0.01,0.07) |  | 0.14(0.05,0.30) | 0.05(0.02,0.10) |  | 156.75(-30.97,547.71) | 0.703212971 |
| Bangladesh | 24.80(6.48,67.04) | 0.05(0.01,0.14) |  | 30.13(9.95,74.22) | 0.07(0.02,0.16) |  | 21.47(-73.37,461.75) | 0.137470161 |
| Barbados | 0.23(0.14,0.35) | 0.38(0.23,0.57) |  | 0.19(0.12,0.28) | 0.40(0.24,0.60) |  | -19.42(-54.99,28.82) | 0.841951981 |
| Belarus | 2.05(1.24,3.34) | 0.09(0.05,0.14) |  | 2.11(0.67,4.32) | 0.13(0.04,0.27) |  | 2.78(-66.96,121.81) | 2.636718324 |
| Belgium | 2.77(1.59,4.53) | 0.15(0.09,0.25) |  | 6.03(2.47,9.66) | 0.32(0.13,0.51) |  | 118.17(-25.40,346.88) | 2.013419476 |
| Belize | 0.05(0.03,0.10) | 0.06(0.03,0.12) |  | 0.08(0.05,0.12) | 0.07(0.04,0.10) |  | 67.40(-30.73,181.66) | 1.085390005 |
| Benin | 15.53(6.17,26.12) | 0.64(0.25,1.08) |  | 37.19(18.46,57.20) | 0.61(0.30,0.94) |  | 139.46(35.97,351.77) | 0.051565884 |
| Bermuda | 0.03(0.01,0.05) | 0.23(0.11,0.38) |  | 0.04(0.01,0.06) | 0.42(0.15,0.72) |  | 29.01(-60.00,274.84) | 2.222802816 |
| Bhutan | 0.13(0.03,0.34) | 0.05(0.01,0.13) |  | 0.12(0.03,0.30) | 0.06(0.02,0.16) |  | -11.02(-84.64,551.86) | -0.376462887 |
| Bolivia (Plurinational State of) | 4.42(2.07,8.44) | 0.16(0.08,0.31) |  | 4.36(1.80,8.30) | 0.12(0.05,0.24) |  | -1.39(-70.57,153.21) | -1.828198129 |
| Bosnia and Herzegovina | 0.31(0.11,0.76) | 0.03(0.01,0.07) |  | 0.26(0.09,0.59) | 0.05(0.02,0.12) |  | -17.02(-79.42,195.85) | 2.230609784 |
| Botswana | 0.42(0.21,0.79) | 0.07(0.04,0.13) |  | 1.11(0.47,2.08) | 0.16(0.07,0.30) |  | 163.96(0.11,495.58) | 2.905694698 |
| Brazil | 63.00(44.42,92.92) | 0.12(0.09,0.18) |  | 82.02(41.18,116.79) | 0.17(0.09,0.24) |  | 30.19(-40.11,84.25) | 1.433221505 |
| Brunei Darussalam | 0.08(0.04,0.16) | 0.09(0.05,0.17) |  | 0.10(0.05,0.16) | 0.10(0.05,0.17) |  | 12.67(-50.62,150.21) | 0.322609435 |
| Bulgaria | 0.45(0.20,0.93) | 0.03(0.01,0.05) |  | 0.39(0.17,0.64) | 0.04(0.02,0.07) |  | -14.93(-73.80,135.24) | 1.185387212 |
| Burkina Faso | 32.08(13.66,53.55) | 0.68(0.29,1.13) |  | 64.77(34.08,99.11) | 0.62(0.33,0.96) |  | 101.91(22.66,265.08) | 0.095484569 |
| Burundi | 34.57(13.40,60.75) | 1.32(0.51,2.32) |  | 31.76(16.04,54.88) | 0.54(0.27,0.94) |  | -8.13(-45.33,81.09) | -2.594629701 |
| Cabo Verde | 0.20(0.09,0.34) | 0.12(0.06,0.21) |  | 0.95(0.35,1.61) | 0.66(0.25,1.12) |  | 384.26(60.56,867.88) | 4.391317829 |
| Cambodia | 1.41(0.26,4.47) | 0.03(0.01,0.10) |  | 1.11(0.43,2.42) | 0.02(0.01,0.05) |  | -21.50(-78.93,240.66) | -1.735431486 |
| Cameroon | 31.31(14.32,50.13) | 0.64(0.29,1.03) |  | 93.35(51.74,143.04) | 0.69(0.38,1.06) |  | 198.12(84.42,430.32) | 0.588780656 |
| Canada | 11.43(6.66,17.69) | 0.20(0.12,0.31) |  | 13.80(6.10,23.69) | 0.22(0.10,0.38) |  | 20.82(-46.03,100.85) | 0.007435119 |
| Central African Republic | 6.63(2.26,12.06) | 0.54(0.19,0.99) |  | 9.39(4.10,15.71) | 0.41(0.18,0.69) |  | 41.71(-14.18,158.04) | -0.756689083 |
| Chad | 15.41(6.66,25.92) | 0.53(0.23,0.89) |  | 55.69(26.92,88.43) | 0.62(0.30,0.98) |  | 261.33(117.47,521.41) | 0.923616333 |
| Chile | 5.54(2.68,9.12) | 0.14(0.07,0.23) |  | 9.88(5.42,14.32) | 0.27(0.15,0.39) |  | 78.19(-22.83,315.77) | 2.740400654 |
| China | 160.99(68.69,257.26) | 0.05(0.02,0.08) |  | 172.53(90.69,298.14) | 0.07(0.03,0.11) |  | 7.17(-51.07,180.87) | 0.013902394 |
| Colombia | 14.06(8.52,21.48) | 0.12(0.07,0.18) |  | 27.78(12.59,48.75) | 0.26(0.12,0.46) |  | 97.49(-11.88,249.80) | 3.266363661 |
| Comoros | 1.94(0.79,3.20) | 0.91(0.37,1.50) |  | 1.59(0.90,2.46) | 0.66(0.37,1.03) |  | -17.91(-59.56,74.98) | -1.30408248 |
| Congo | 4.15(1.70,7.01) | 0.39(0.16,0.67) |  | 4.63(2.47,7.04) | 0.24(0.13,0.36) |  | 11.42(-31.84,119.04) | -1.660759746 |
| Cook Islands | 0.00(0.00,0.00) | 0.01(0.01,0.03) |  | 0.00(0.00,0.00) | 0.04(0.01,0.10) |  | 62.45(-71.29,383.12) | 2.413131496 |
| Costa Rica | 2.08(1.09,3.35) | 0.19(0.10,0.30) |  | 2.80(1.01,5.55) | 0.28(0.10,0.55) |  | 34.77(-49.05,308.20) | 1.182720927 |
| Croatia | 36.17(17.31,54.97) | 0.63(0.30,0.96) |  | 68.88(36.71,110.24) | 0.60(0.32,0.95) |  | 12.90(-69.87,254.13) | 0.147862434 |
| Cuba | 1.16(0.54,2.05) | 0.12(0.05,0.21) |  | 1.31(0.43,2.39) | 0.22(0.07,0.40) |  | -53.48(-78.14,3.82) | 2.798863637 |
| Cyprus | 9.94(5.73,16.35) | 0.40(0.23,0.65) |  | 4.63(2.34,8.13) | 0.26(0.13,0.46) |  | 61.63(-26.36,277.10) | 0.922092832 |
| Czechia | 0.34(0.16,0.62) | 0.17(0.08,0.32) |  | 0.55(0.29,0.93) | 0.25(0.13,0.43) |  | 16.81(-72.48,214.00) | 1.776863009 |
| C么te d'Ivoire | 1.89(1.18,3.26) | 0.09(0.05,0.15) |  | 2.21(0.59,4.70) | 0.13(0.03,0.27) |  | 90.40(13.76,235.59) | 1.069778803 |
| Democratic People's Republic of Korea | 1.80(0.78,3.99) | 0.03(0.01,0.07) |  | 1.84(0.65,3.93) | 0.04(0.01,0.08) |  | 1.74(-62.88,156.38) | 0.598095622 |
| Democratic Republic of the Congo | 87.00(31.08,156.73) | 0.49(0.18,0.89) |  | 81.10(39.58,129.15) | 0.21(0.10,0.34) |  | -6.78(-43.90,109.61) | -2.292600135 |
| Denmark | 1.80(1.07,2.77) | 0.20(0.12,0.31) |  | 1.73(0.79,2.92) | 0.18(0.08,0.31) |  | -3.47(-59.21,68.50) | -0.085282278 |
| Djibouti | 1.23(0.61,1.90) | 0.70(0.35,1.09) |  | 2.57(1.38,4.24) | 0.62(0.33,1.03) |  | 109.49(19.16,265.56) | -0.427300463 |
| Dominica | 0.02(0.01,0.04) | 0.07(0.04,0.15) |  | 0.02(0.01,0.03) | 0.11(0.05,0.22) |  | -13.12(-59.63,110.84) | 1.83569505 |
| Dominican Republic | 1.21(0.63,2.59) | 0.04(0.02,0.10) |  | 2.70(1.11,5.41) | 0.09(0.04,0.18) |  | 123.60(-28.38,488.94) | 2.363281709 |
| Ecuador | 2.70(1.81,4.13) | 0.07(0.05,0.11) |  | 7.15(2.89,12.14) | 0.14(0.06,0.24) |  | 164.76(-1.92,343.40) | 2.524486548 |
| Egypt | 7.92(1.77,28.81) | 0.04(0.01,0.13) |  | 11.31(1.33,45.96) | 0.03(0.00,0.12) |  | 42.88(-76.76,276.87) | -0.441497004 |
| El Salvador | 1.04(0.61,1.75) | 0.05(0.03,0.08) |  | 1.53(0.81,2.58) | 0.08(0.04,0.14) |  | 47.34(-42.05,175.05) | 1.859276386 |
| Equatorial Guinea | 0.99(0.37,1.73) | 0.50(0.19,0.88) |  | 2.07(0.83,4.34) | 0.35(0.14,0.74) |  | 108.70(-11.60,455.20) | -1.950622359 |
| Eritrea | 14.10(6.04,22.87) | 0.89(0.38,1.44) |  | 17.67(9.40,28.43) | 0.70(0.37,1.13) |  | 25.34(-26.94,138.25) | -0.83784071 |
| Estonia | 0.41(0.24,0.78) | 0.12(0.07,0.22) |  | 0.33(0.09,0.65) | 0.15(0.04,0.30) |  | -19.26(-84.40,127.24) | 1.584739232 |
| Eswatini | 0.36(0.16,0.75) | 0.09(0.04,0.19) |  | 0.56(0.24,1.06) | 0.14(0.06,0.26) |  | 54.24(-33.53,261.58) | 1.749681707 |
| Ethiopia | 248.29(75.92,491.50) | 1.02(0.31,2.02) |  | 220.65(116.10,353.65) | 0.50(0.26,0.80) |  | -11.13(-53.16,93.19) | -2.603400959 |
| Fiji | 0.07(0.03,0.14) | 0.03(0.01,0.05) |  | 0.18(0.07,0.37) | 0.07(0.03,0.14) |  | 145.83(-9.42,647.22) | 3.274045336 |
| Finland | 1.43(0.78,2.49) | 0.15(0.08,0.26) |  | 1.39(0.49,2.41) | 0.16(0.06,0.28) |  | -3.28(-64.77,89.31) | 0.311065494 |
| France | 14.72(9.46,22.48) | 0.13(0.08,0.19) |  | 30.40(9.60,64.85) | 0.26(0.08,0.56) |  | 106.56(-31.05,347.08) | 2.260921234 |
| Gabon | 1.29(0.58,2.08) | 0.32(0.14,0.51) |  | 2.05(1.05,3.59) | 0.32(0.16,0.56) |  | 59.38(-10.65,201.93) | 0.360860111 |
| Gambia | 2.61(1.23,4.37) | 0.57(0.27,0.95) |  | 5.01(2.64,8.35) | 0.50(0.27,0.84) |  | 91.86(4.53,254.22) | -0.468159955 |
| Georgia | 2.78(0.78,5.21) | 0.20(0.06,0.38) |  | 0.53(0.23,0.90) | 0.07(0.03,0.12) |  | -80.84(-93.54,-14.58) | -3.640449429 |
| Germany | 12.42(7.52,20.46) | 0.10(0.06,0.16) |  | 18.37(6.91,32.20) | 0.15(0.06,0.27) |  | 47.82(-48.95,179.63) | 1.481054433 |
| Ghana | 73.72(23.19,133.95) | 1.10(0.35,1.99) |  | 55.85(32.39,97.66) | 0.43(0.25,0.76) |  | -24.24(-63.31,188.81) | -4.042845877 |
| Greece | 1.95(0.71,3.61) | 0.10(0.04,0.18) |  | 1.97(1.10,2.86) | 0.14(0.08,0.20) |  | 0.69(-52.09,188.45) | 1.216982685 |
| Greenland | 0.02(0.00,0.05) | 0.16(0.02,0.36) |  | 0.01(0.00,0.02) | 0.06(0.02,0.15) |  | -66.18(-93.09,140.20) | -1.606143407 |
| Grenada | 0.06(0.03,0.09) | 0.17(0.10,0.28) |  | 0.05(0.03,0.08) | 0.24(0.14,0.38) |  | -7.33(-45.97,47.58) | 1.352994855 |
| Guam | 0.01(0.01,0.03) | 0.04(0.01,0.08) |  | 0.04(0.02,0.06) | 0.10(0.06,0.15) |  | 146.87(10.62,546.68) | 5.826524321 |
| Guatemala | 3.74(2.14,7.74) | 0.09(0.05,0.19) |  | 3.33(1.94,5.12) | 0.07(0.04,0.10) |  | -10.88(-67.07,73.85) | -0.317824661 |
| Guinea | 19.10(8.28,31.59) | 0.69(0.30,1.15) |  | 27.38(15.23,48.33) | 0.45(0.25,0.80) |  | 43.34(-16.12,170.57) | -0.820132022 |
| Guinea-Bissau | 3.82(1.56,6.51) | 0.79(0.32,1.35) |  | 4.45(2.24,7.19) | 0.50(0.25,0.80) |  | 16.52(-29.34,141.57) | -1.195083418 |
| Guyana | 0.24(0.13,0.39) | 0.08(0.05,0.13) |  | 0.09(0.05,0.19) | 0.04(0.02,0.09) |  | -60.15(-82.63,14.92) | 0.060226775 |
| Haiti | 6.57(1.16,21.94) | 0.24(0.04,0.81) |  | 8.21(2.14,21.06) | 0.19(0.05,0.48) |  | 24.83(-52.25,274.44) | -0.478599879 |
| Honduras | 1.09(0.51,2.24) | 0.05(0.02,0.10) |  | 1.09(0.51,1.97) | 0.03(0.02,0.06) |  | -0.74(-59.46,153.02) | -1.735588209 |
| Hungary | 1.36(0.75,2.29) | 0.06(0.04,0.11) |  | 1.68(0.47,3.17) | 0.12(0.03,0.23) |  | 23.01(-66.88,229.53) | 2.237867344 |
| Iceland | 0.10(0.05,0.16) | 0.15(0.09,0.25) |  | 0.08(0.04,0.14) | 0.12(0.06,0.21) |  | -17.05(-61.88,55.67) | 0.023719021 |
| India | 178.87(55.64,345.90) | 0.05(0.02,0.11) |  | 126.71(76.53,201.62) | 0.03(0.02,0.06) |  | -29.16(-74.55,142.33) | -1.739466721 |
| Indonesia | 9.11(2.29,19.51) | 0.01(0.00,0.03) |  | 13.04(6.13,23.10) | 0.02(0.01,0.03) |  | 43.17(-36.23,282.10) | -0.350933877 |
| Iran (Islamic Republic of) | 9.61(4.79,20.04) | 0.04(0.02,0.08) |  | 15.37(5.98,29.71) | 0.08(0.03,0.15) |  | 59.94(-66.44,396.68) | 1.985359545 |
| Iraq | 11.81(1.99,30.64) | 0.14(0.02,0.37) |  | 13.82(4.53,29.67) | 0.10(0.03,0.22) |  | 17.01(-59.14,326.77) | -0.890331658 |
| Ireland | 1.03(0.61,1.70) | 0.11(0.06,0.17) |  | 2.31(0.94,4.12) | 0.23(0.09,0.41) |  | 123.50(-12.89,375.20) | 2.255226696 |
| Israel | 4.22(2.48,6.95) | 0.28(0.16,0.45) |  | 11.45(6.36,16.87) | 0.44(0.24,0.64) |  | 171.12(33.51,352.79) | 1.78036591 |
| Italy | 15.07(9.84,24.75) | 0.16(0.11,0.27) |  | 23.31(7.84,46.83) | 0.31(0.10,0.62) |  | 54.64(-55.69,276.00) | 2.364428267 |
| Jamaica | 0.94(0.49,1.76) | 0.11(0.06,0.21) |  | 0.62(0.33,1.13) | 0.11(0.06,0.19) |  | -33.51(-68.37,21.61) | 0.175576752 |
| Japan | 24.74(9.74,43.30) | 0.11(0.04,0.19) |  | 29.55(12.30,44.41) | 0.19(0.08,0.29) |  | 19.41(-53.67,254.26) | 1.824814428 |
| Jordan | 1.70(0.73,3.41) | 0.10(0.04,0.21) |  | 6.68(3.35,12.29) | 0.18(0.09,0.34) |  | 291.89(54.60,876.03) | 1.654616467 |
| Kazakhstan | 2.44(1.12,4.93) | 0.05(0.02,0.09) |  | 2.24(1.00,4.12) | 0.04(0.02,0.08) |  | -8.34(-67.82,107.86) | -0.371765321 |
| Kenya | 31.64(16.87,46.81) | 0.28(0.15,0.42) |  | 47.66(30.01,64.93) | 0.26(0.16,0.35) |  | 50.62(2.35,139.78) | 0.576209619 |
| Kiribati | 0.00(0.00,0.00) | 0.00(0.00,0.01) |  | 0.00(0.00,0.00) | 0.00(0.00,0.01) |  | 26.55(-64.79,226.07) | -0.932596727 |
| Kuwait | 1.05(0.58,1.77) | 0.19(0.10,0.32) |  | 1.08(0.53,1.85) | 0.13(0.06,0.22) |  | 2.88(-53.04,88.81) | -0.36023314 |
| Kyrgyzstan | 0.50(0.26,1.05) | 0.03(0.02,0.06) |  | 0.71(0.31,1.37) | 0.03(0.01,0.06) |  | 41.84(-53.01,244.27) | 0.050448034 |
| Lao People's Democratic Republic | 0.44(0.06,1.29) | 0.02(0.00,0.07) |  | 0.55(0.21,1.17) | 0.02(0.01,0.05) |  | 26.20(-65.29,663.49) | -2.32843556 |
| Latvia | 0.50(0.26,0.94) | 0.09(0.05,0.17) |  | 0.27(0.07,0.55) | 0.09(0.02,0.18) |  | -44.86(-89.72,65.53) | 0.587712986 |
| Lebanon | 1.07(0.46,2.17) | 0.10(0.04,0.21) |  | 2.28(0.85,4.75) | 0.18(0.07,0.37) |  | 112.58(-36.08,556.89) | 1.955929134 |
| Lesotho | 0.44(0.21,0.85) | 0.06(0.03,0.12) |  | 0.78(0.33,1.51) | 0.12(0.05,0.24) |  | 78.51(-27.07,282.11) | 2.833673718 |
| Liberia | 9.99(4.39,16.82) | 0.88(0.39,1.49) |  | 12.44(6.50,18.84) | 0.57(0.30,0.86) |  | 24.42(-30.65,139.01) | -1.588007926 |
| Libya | 2.12(0.73,4.83) | 0.12(0.04,0.27) |  | 4.57(1.57,10.21) | 0.31(0.11,0.68) |  | 115.48(-16.62,519.28) | 3.547731888 |
| Lithuania | 0.75(0.40,1.27) | 0.09(0.05,0.15) |  | 0.46(0.13,1.08) | 0.11(0.03,0.26) |  | -38.54(-86.51,117.90) | 0.214167829 |
| Luxembourg | 0.11(0.04,0.19) | 0.17(0.07,0.29) |  | 0.33(0.18,0.51) | 0.33(0.18,0.51) |  | 201.62(56.07,589.79) | 1.622209306 |
| Madagascar | 44.06(22.00,65.53) | 0.81(0.40,1.20) |  | 59.22(36.00,86.65) | 0.50(0.31,0.74) |  | 34.43(-17.55,128.69) | -1.042485241 |
| Malawi | 96.74(44.03,158.42) | 2.13(0.97,3.48) |  | 106.09(49.42,202.86) | 1.31(0.61,2.50) |  | 9.67(-38.09,113.21) | -1.235086831 |
| Malaysia | 1.35(0.55,3.04) | 0.02(0.01,0.05) |  | 2.04(0.64,4.65) | 0.03(0.01,0.06) |  | 51.01(-57.21,311.15) | 0.425393844 |
| Maldives | 0.06(0.02,0.15) | 0.05(0.02,0.15) |  | 0.07(0.03,0.13) | 0.07(0.03,0.13) |  | 23.21(-69.95,390.15) | 1.3840847 |
| Mali | 20.99(9.53,34.04) | 0.51(0.23,0.82) |  | 37.79(21.55,61.42) | 0.33(0.19,0.53) |  | 80.10(0.17,232.49) | -1.086957772 |
| Malta | 0.11(0.05,0.18) | 0.12(0.06,0.21) |  | 0.24(0.12,0.45) | 0.38(0.19,0.71) |  | 124.01(22.67,537.99) | 2.811033943 |
| Marshall Islands | 0.00(0.00,0.00) | 0.01(0.00,0.02) |  | 0.00(0.00,0.01) | 0.03(0.01,0.06) |  | 105.68(-25.02,457.53) | 2.574931654 |
| Mauritania | 4.17(2.14,6.86) | 0.45(0.23,0.74) |  | 9.16(5.26,14.33) | 0.49(0.28,0.77) |  | 119.77(25.17,330.79) | -0.029236542 |
| Mauritius | 0.05(0.03,0.07) | 0.02(0.01,0.02) |  | 0.05(0.03,0.08) | 0.02(0.01,0.04) |  | -2.16(-44.68,47.95) | 1.033976995 |
| Mexico | 24.56(15.68,42.44) | 0.07(0.05,0.13) |  | 36.95(18.40,54.85) | 0.12(0.06,0.17) |  | 50.45(-39.77,135.89) | 1.742073989 |
| Micronesia (Federated States of) | 0.00(0.00,0.01) | 0.01(0.00,0.02) |  | 0.01(0.00,0.02) | 0.02(0.01,0.05) |  | 49.71(-57.59,450.39) | 2.933458266 |
| Monaco | 0.00(0.00,0.01) | 0.05(0.01,0.16) |  | 0.00(0.00,0.01) | 0.10(0.03,0.23) |  | 160.67(5.45,721.78) | 1.624651894 |
| Mongolia | 0.28(0.07,0.86) | 0.03(0.01,0.10) |  | 0.63(0.25,1.29) | 0.06(0.02,0.12) |  | 126.01(-45.77,847.36) | 1.836548924 |
| Montenegro | 0.36(0.22,0.60) | 0.22(0.13,0.37) |  | 0.15(0.07,0.29) | 0.14(0.06,0.26) |  | -56.44(-81.83,-10.16) | -0.914439685 |
| Morocco | 5.95(2.07,13.06) | 0.06(0.02,0.13) |  | 7.28(3.01,13.49) | 0.07(0.03,0.14) |  | 22.44(-58.52,232.06) | 1.075209533 |
| Mozambique | 10.97(4.26,25.41) | 0.18(0.07,0.41) |  | 11.81(4.88,29.88) | 0.08(0.03,0.21) |  | 7.61(-43.75,121.25) | -2.049228592 |
| Myanmar | 5.28(0.82,16.89) | 0.04(0.01,0.11) |  | 3.69(1.42,7.75) | 0.02(0.01,0.05) |  | -30.00(-81.08,222.75) | -2.08109103 |
| Namibia | 0.69(0.34,1.21) | 0.11(0.06,0.20) |  | 1.90(0.89,3.46) | 0.23(0.11,0.42) |  | 176.42(24.61,517.07) | 2.731085309 |
| Nauru | 0.00(0.00,0.00) | 0.02(0.01,0.04) |  | 0.00(0.00,0.00) | 0.05(0.01,0.11) |  | 102.18(-35.42,440.02) | 2.314855486 |
| Nepal | 3.40(1.00,8.32) | 0.04(0.01,0.10) |  | 3.88(1.20,9.67) | 0.04(0.01,0.10) |  | 14.10(-76.27,377.29) | -0.547558192 |
| Netherlands | 8.54(5.13,13.05) | 0.31(0.19,0.48) |  | 11.05(4.96,16.98) | 0.41(0.18,0.63) |  | 29.30(-46.17,109.63) | 1.220951041 |
| New Zealand | 1.54(1.03,2.32) | 0.19(0.13,0.29) |  | 2.16(1.30,3.28) | 0.22(0.13,0.33) |  | 39.88(-22.97,108.28) | 0.060203935 |
| Nicaragua | 1.58(0.86,2.67) | 0.09(0.05,0.15) |  | 2.11(1.28,3.38) | 0.11(0.06,0.17) |  | 34.15(-34.14,171.23) | 1.017640525 |
| Niger | 35.27(13.19,64.12) | 0.87(0.32,1.58) |  | 60.47(27.15,99.10) | 0.47(0.21,0.78) |  | 71.47(-2.71,235.30) | -1.770117592 |
| Nigeria | 292.58(143.17,435.33) | 0.75(0.37,1.11) |  | 726.18(367.20,1029.37) | 0.71(0.36,1.01) |  | 148.20(71.46,274.09) | 0.143601346 |
| Niue | 0.00(0.00,0.00) | 0.02(0.01,0.04) |  | 0.00(0.00,0.00) | 0.09(0.02,0.24) |  | 109.27(-31.97,523.42) | 4.254875169 |
| North Macedonia | 0.07(0.03,0.18) | 0.01(0.01,0.03) |  | 0.09(0.04,0.17) | 0.03(0.01,0.05) |  | 26.35(-73.37,308.21) | 3.082979263 |
| Northern Mariana Islands | 0.00(0.00,0.00) | 0.01(0.00,0.03) |  | 0.00(0.00,0.01) | 0.03(0.01,0.05) |  | 126.33(-25.21,918.87) | 3.91652757 |
| Norway | 0.72(0.46,1.30) | 0.09(0.06,0.16) |  | 0.91(0.40,1.48) | 0.10(0.04,0.16) |  | 26.74(-55.86,93.82) | 0.442012224 |
| Oman | 0.58(0.21,1.31) | 0.07(0.02,0.16) |  | 1.97(0.66,3.74) | 0.16(0.05,0.31) |  | 240.04(18.70,830.12) | 2.764502477 |
| Pakistan | 67.58(26.55,122.74) | 0.14(0.05,0.25) |  | 171.86(69.42,336.82) | 0.20(0.08,0.39) |  | 154.29(23.78,397.00) | 1.318165981 |
| Palau | 0.00(0.00,0.00) | 0.02(0.01,0.04) |  | 0.00(0.00,0.00) | 0.04(0.02,0.06) |  | 15.90(-48.64,142.87) | 1.578637537 |
| Palestine | 0.52(0.19,1.13) | 0.05(0.02,0.12) |  | 1.43(0.51,2.92) | 0.08(0.03,0.16) |  | 176.44(-28.76,700.72) | 1.366113636 |
| Panama | 1.37(0.84,2.18) | 0.16(0.10,0.26) |  | 3.76(2.41,5.35) | 0.33(0.21,0.46) |  | 174.51(58.92,317.55) | 1.911562392 |
| Papua New Guinea | 0.39(0.10,1.01) | 0.02(0.01,0.06) |  | 1.58(0.38,3.66) | 0.04(0.01,0.09) |  | 305.67(68.36,974.28) | 1.923584175 |
| Paraguay | 1.10(0.56,1.98) | 0.07(0.03,0.12) |  | 2.08(0.92,3.78) | 0.10(0.05,0.19) |  | 89.10(-18.29,316.89) | 1.544229824 |
| Peru | 10.80(5.79,19.10) | 0.13(0.07,0.23) |  | 20.37(9.85,34.92) | 0.21(0.10,0.37) |  | 88.63(-28.89,343.85) | 1.928964655 |
| Philippines | 10.13(3.87,16.88) | 0.04(0.02,0.07) |  | 9.92(6.29,16.01) | 0.03(0.02,0.05) |  | -2.08(-48.01,100.83) | -0.865922997 |
| Poland | 3.78(1.11,7.54) | 0.04(0.01,0.08) |  | 6.37(2.27,9.49) | 0.11(0.04,0.16) |  | 68.50(-56.12,586.07) | 3.305530115 |
| Portugal | 2.82(1.59,5.73) | 0.13(0.08,0.27) |  | 3.67(1.42,6.16) | 0.27(0.10,0.45) |  | 30.05(-67.43,149.48) | 1.990279433 |
| Puerto Rico | 2.44(1.26,3.92) | 0.25(0.13,0.39) |  | 1.22(0.52,1.87) | 0.27(0.12,0.42) |  | -50.12(-79.84,-2.59) | 0.984692162 |
| Qatar | 0.03(0.01,0.09) | 0.03(0.01,0.07) |  | 0.30(0.09,0.69) | 0.06(0.02,0.14) |  | 777.23(199.53,2058.78) | 2.627812135 |
| Republic of Korea | 4.83(1.45,10.54) | 0.04(0.01,0.09) |  | 5.26(2.13,10.16) | 0.09(0.04,0.17) |  | 8.98(-70.13,311.05) | 2.460386479 |
| Republic of Moldova | 3.56(1.65,6.95) | 0.29(0.13,0.56) |  | 1.24(0.61,1.94) | 0.24(0.12,0.37) |  | -65.17(-86.14,-18.02) | 0.201369255 |
| Romania | 4.97(1.93,11.35) | 0.09(0.03,0.20) |  | 5.62(2.94,8.74) | 0.19(0.10,0.29) |  | 13.04(-63.11,214.82) | 2.766400581 |
| Russian Federation | 43.31(23.95,66.89) | 0.12(0.07,0.19) |  | 29.75(12.46,43.65) | 0.11(0.05,0.17) |  | -31.32(-72.17,8.11) | 0.56605804 |
| Rwanda | 47.96(20.35,77.63) | 1.41(0.60,2.29) |  | 31.13(17.45,49.49) | 0.63(0.35,1.00) |  | -35.11(-63.51,24.90) | -3.235669957 |
| Saint Kitts and Nevis | 0.01(0.00,0.01) | 0.04(0.02,0.07) |  | 0.01(0.00,0.02) | 0.09(0.05,0.16) |  | 45.24(-31.47,245.17) | 3.503191698 |
| Saint Lucia | 0.04(0.03,0.07) | 0.08(0.05,0.14) |  | 0.05(0.03,0.07) | 0.16(0.09,0.24) |  | 7.62(-40.96,84.43) | 1.944612182 |
| Saint Vincent and the Grenadines | 0.04(0.01,0.07) | 0.09(0.02,0.18) |  | 0.03(0.02,0.05) | 0.13(0.08,0.21) |  | -10.90(-55.04,291.81) | 1.19894277 |
| Samoa | 0.01(0.00,0.02) | 0.01(0.00,0.03) |  | 0.02(0.01,0.04) | 0.02(0.01,0.05) |  | 92.93(-43.49,553.65) | 1.407690741 |
| San Marino | 0.01(0.01,0.03) | 0.35(0.14,0.76) |  | 0.02(0.01,0.04) | 0.43(0.14,0.91) |  | 31.67(-52.83,253.39) | 0.617271245 |
| Sao Tome and Principe | 0.41(0.19,0.67) | 0.72(0.34,1.18) |  | 0.30(0.17,0.53) | 0.39(0.21,0.69) |  | -25.59(-63.53,60.72) | -1.565762566 |
| Saudi Arabia | 4.32(1.84,8.50) | 0.07(0.03,0.13) |  | 8.93(2.39,17.14) | 0.12(0.03,0.23) |  | 106.75(-49.33,453.66) | 2.086403383 |
| Senegal | 24.62(10.90,38.43) | 0.67(0.30,1.05) |  | 27.18(15.95,43.82) | 0.43(0.25,0.69) |  | 10.39(-35.49,116.37) | -1.419224723 |
| Serbia | 1.12(0.45,2.55) | 0.05(0.02,0.12) |  | 0.75(0.23,1.64) | 0.06(0.02,0.12) |  | -33.35(-82.53,96.99) | 0.420025851 |
| Seychelles | 0.00(0.00,0.00) | 0.00(0.00,0.00) |  | 0.00(0.00,0.00) | 0.00(0.00,0.00) |  | 159.86(-32.71,1109.34) | 2.745093513 |
| Sierra Leone | 15.13(6.38,25.33) | 0.83(0.35,1.40) |  | 22.08(12.02,33.51) | 0.62(0.34,0.94) |  | 45.95(-16.58,176.28) | -0.958286541 |
| Singapore | 0.55(0.30,1.04) | 0.09(0.05,0.16) |  | 1.38(0.42,2.44) | 0.17(0.05,0.30) |  | 150.34(-38.96,550.15) | 2.555667147 |
| Slovakia | 0.53(0.24,1.06) | 0.04(0.02,0.08) |  | 0.94(0.39,2.15) | 0.11(0.05,0.25) |  | 77.21(-43.09,434.48) | 3.301350369 |
| Slovenia | 0.29(0.18,0.51) | 0.07(0.04,0.12) |  | 0.21(0.06,0.36) | 0.07(0.02,0.12) |  | -28.96(-82.58,40.27) | 0.784967035 |
| Solomon Islands | 0.02(0.01,0.04) | 0.01(0.00,0.02) |  | 0.06(0.02,0.13) | 0.02(0.01,0.05) |  | 220.81(12.23,845.71) | 1.996462936 |
| Somalia | 30.05(11.41,56.74) | 0.77(0.29,1.46) |  | 56.84(25.10,98.54) | 0.55(0.24,0.95) |  | 89.11(11.31,245.46) | -0.94924698 |
| South Africa | 5.20(2.56,8.46) | 0.04(0.02,0.06) |  | 10.38(6.23,16.09) | 0.07(0.04,0.11) |  | 99.41(9.64,301.64) | 1.705217044 |
| South Sudan | 24.78(11.24,41.31) | 0.94(0.43,1.57) |  | 48.98(23.62,78.24) | 1.14(0.55,1.82) |  | 97.67(29.09,217.85) | 0.713520295 |
| Spain | 15.20(8.68,24.48) | 0.19(0.11,0.31) |  | 21.67(7.47,41.00) | 0.33(0.12,0.63) |  | 42.59(-56.80,172.24) | 1.641435019 |
| Sri Lanka | 2.24(1.00,4.64) | 0.04(0.02,0.08) |  | 3.54(1.35,6.87) | 0.07(0.03,0.13) |  | 58.45(-52.91,300.40) | 2.294803117 |
| Sudan | 6.70(1.79,22.73) | 0.08(0.02,0.26) |  | 14.55(4.17,35.78) | 0.09(0.03,0.22) |  | 117.17(-40.26,548.36) | 0.580283346 |
| Suriname | 0.05(0.02,0.09) | 0.04(0.02,0.07) |  | 0.09(0.04,0.17) | 0.06(0.03,0.12) |  | 87.72(-25.53,373.44) | 2.616940902 |
| Sweden | 1.19(0.34,2.32) | 0.08(0.02,0.15) |  | 2.78(1.43,4.48) | 0.15(0.08,0.25) |  | 132.63(-1.52,560.11) | 2.584017001 |
| Switzerland | 1.92(1.08,3.35) | 0.17(0.09,0.29) |  | 2.15(0.85,4.19) | 0.16(0.06,0.31) |  | 12.18(-63.70,142.69) | -0.331275704 |
| Syrian Arab Republic | 1.13(0.52,2.20) | 0.02(0.01,0.04) |  | 2.34(0.78,4.38) | 0.06(0.02,0.12) |  | 107.47(-40.91,426.26) | 3.483113354 |
| Taiwan (Province of China) | 3.57(2.25,6.08) | 0.06(0.04,0.11) |  | 3.42(1.16,5.84) | 0.12(0.04,0.20) |  | -4.30(-70.74,67.89) | 2.708669803 |
| Tajikistan | 0.04(0.01,0.10) | 0.00(0.00,0.00) |  | 0.05(0.02,0.11) | 0.00(0.00,0.00) |  | 22.33(-64.97,316.16) | -1.732150719 |
| Thailand | 5.08(1.03,12.13) | 0.03(0.01,0.07) |  | 6.26(2.53,12.45) | 0.06(0.03,0.13) |  | 23.35(-52.73,326.07) | 1.168008986 |
| Timor-Leste | 0.08(0.01,0.25) | 0.02(0.00,0.08) |  | 0.08(0.03,0.19) | 0.02(0.01,0.04) |  | -1.20(-69.96,364.09) | -2.079931599 |
| Togo | 9.78(4.65,15.42) | 0.55(0.26,0.87) |  | 15.46(8.34,23.97) | 0.47(0.25,0.72) |  | 58.02(-8.40,207.60) | -0.328354873 |
| Tokelau | 0.00(0.00,0.00) | 0.01(0.01,0.03) |  | 0.00(0.00,0.00) | 0.05(0.02,0.12) |  | 143.55(-32.47,648.39) | 3.087174199 |
| Tonga | 0.02(0.01,0.04) | 0.04(0.02,0.09) |  | 0.04(0.01,0.09) | 0.09(0.03,0.22) |  | 101.57(-30.14,424.72) | 1.924165329 |
| Trinidad and Tobago | 0.48(0.29,0.84) | 0.12(0.07,0.21) |  | 0.40(0.22,0.62) | 0.15(0.08,0.23) |  | -16.63(-58.20,49.37) | 1.552704121 |
| Tunisia | 4.24(1.39,10.02) | 0.14(0.04,0.32) |  | 5.21(1.98,10.52) | 0.19(0.07,0.38) |  | 23.05(-63.83,258.34) | 1.068527038 |
| Turkey | 47.17(21.63,86.15) | 0.23(0.11,0.42) |  | 68.95(39.59,108.30) | 0.37(0.21,0.58) |  | 46.17(-36.03,244.68) | 1.660960196 |
| Turkmenistan | 0.20(0.08,0.56) | 0.01(0.01,0.04) |  | 0.26(0.13,0.50) | 0.02(0.01,0.03) |  | 26.54(-47.71,208.21) | 0.595658366 |
| Tuvalu | 0.00(0.00,0.00) | 0.03(0.01,0.07) |  | 0.00(0.00,0.00) | 0.03(0.01,0.06) |  | 2.34(-70.52,209.26) | 0.062577021 |
| Uganda | 118.42(67.45,172.77) | 1.41(0.80,2.05) |  | 270.58(152.03,426.93) | 1.36(0.77,2.15) |  | 128.50(48.86,270.42) | -0.112925622 |
| Ukraine | 9.20(4.18,19.53) | 0.08(0.04,0.17) |  | 6.35(2.16,13.61) | 0.10(0.03,0.21) |  | -31.00(-72.30,35.59) | 0.694234194 |
| United Arab Emirates | 0.44(0.18,0.94) | 0.08(0.03,0.16) |  | 0.95(0.41,2.05) | 0.07(0.03,0.15) |  | 115.14(-15.01,373.82) | -0.358726032 |
| United Kingdom | 10.11(2.50,19.03) | 0.09(0.02,0.17) |  | 17.59(7.96,28.30) | 0.15(0.07,0.24) |  | 74.02(-39.91,434.87) | 1.997384927 |
| United Republic of Tanzania | 130.99(56.54,196.54) | 1.08(0.47,1.63) |  | 176.81(99.39,266.29) | 0.72(0.41,1.09) |  | 34.98(-19.43,136.67) | -0.890926507 |
| United States of America | 111.12(74.07,154.43) | 0.20(0.13,0.28) |  | 108.08(66.78,154.10) | 0.18(0.11,0.26) |  | -2.73(-39.08,30.18) | -0.008070117 |
| United States Virgin Islands | 0.02(0.01,0.04) | 0.06(0.02,0.12) |  | 0.01(0.00,0.01) | 0.05(0.02,0.11) |  | -62.32(-86.24,4.16) | 0.47787379 |
| Uruguay | 1.94(1.17,2.96) | 0.24(0.14,0.36) |  | 2.35(1.41,3.51) | 0.36(0.21,0.53) |  | 20.87(-31.57,111.48) | 1.116172198 |
| Uzbekistan | 0.97(0.43,2.08) | 0.01(0.01,0.02) |  | 1.62(0.79,3.53) | 0.02(0.01,0.03) |  | 66.26(-17.87,232.00) | 1.503263599 |
| Vanuatu | 0.01(0.00,0.01) | 0.01(0.00,0.02) |  | 0.02(0.01,0.04) | 0.02(0.01,0.04) |  | 214.93(10.62,787.69) | 2.101781144 |
| Venezuela (Bolivarian Republic of) | 6.99(3.06,12.44) | 0.10(0.04,0.18) |  | 21.46(13.63,31.36) | 0.32(0.21,0.47) |  | 207.08(81.12,489.68) | 3.209660151 |
| Viet Nam | 5.36(1.29,12.48) | 0.02(0.00,0.05) |  | 19.86(3.78,52.22) | 0.08(0.02,0.21) |  | 270.80(4.58,825.40) | 4.910190172 |
| Yemen | 3.65(0.93,10.83) | 0.05(0.01,0.15) |  | 6.70(1.78,18.03) | 0.05(0.01,0.13) |  | 83.62(-46.05,386.86) | 0.041400676 |
| Zambia | 40.24(15.76,62.93) | 1.07(0.42,1.68) |  | 52.52(27.14,87.90) | 0.63(0.33,1.06) |  | 30.52(-26.87,142.89) | -1.716703605 |
| Zimbabwe | 5.25(2.26,9.38) | 0.11(0.05,0.19) |  | 17.36(6.14,29.62) | 0.28(0.10,0.47) |  | 230.65(58.57,526.71) | 4.532768096 |
