# Supplementary material for: Global burden of childhood Burkitt lymphoma (1990–2021): epidemiological trends, regional disparities, and projections for 2035 from the Global Burden of Disease Study
Source: Front Med (Lausanne). 2025 Sep 24;12:1619750. doi: 10.3389/fmed.2025.1619750 (PMC12504260; doi:10.3389/fmed.2025.1619750)
Supplement: Supplementary file 3 [file Table_3.DOCX]

Table S3. Mortality of Burkitt lymphoma in children between 1990 and 2021 at the national level.

| location | 1990 | |  | 2021 | |  | 1990-2021 | |
| --- | --- | --- | --- | --- | --- | --- | --- | --- |
|  | Death cases | Mortality |  | Death cases | Mortality |  | Cases change | EAPC |
| Afghanistan | 4.40(1.19,12.75) | 0.10(0.03,0.30) |  | 8.82(2.72,23.32) | 0.06(0.02,0.16) |  | 100.61(-39.97,430.05) | -1.02(-1.37,-0.67) |
| Albania | 0.03(0.01,0.07) | 0.00(0.00,0.01) |  | 0.02(0.00,0.05) | 0.00(0.00,0.01) |  | -40.38(-89.78,109.84) | 1.33(0.19,2.48) |
| Algeria | 9.27(2.53,19.63) | 0.09(0.02,0.18) |  | 7.67(2.92,16.32) | 0.06(0.02,0.12) |  | -17.25(-66.38,128.06) | -1.21(-1.32,-1.09) |
| American Samoa | 0.00(0.00,0.00) | 0.01(0.00,0.02) |  | 0.00(0.00,0.01) | 0.02(0.01,0.04) |  | 83.91(-43.14,508.01) | 3.19(2.94,3.44) |
| Andorra | 0.01(0.00,0.01) | 0.06(0.02,0.11) |  | 0.00(0.00,0.01) | 0.04(0.02,0.07) |  | -22.77(-71.00,138.85) | -0.93(-1.39,-0.47) |
| Angola | 32.34(9.38,61.24) | 0.69(0.20,1.30) |  | 41.14(18.51,64.32) | 0.27(0.12,0.42) |  | 27.24(-26.00,285.94) | -2.93(-3.23,-2.63) |
| Antigua and Barbuda | 0.01(0.01,0.02) | 0.05(0.03,0.08) |  | 0.01(0.01,0.01) | 0.05(0.03,0.07) |  | -13.17(-43.46,72.31) | -0.32(-1.05,0.42) |
| Argentina | 9.62(6.08,14.14) | 0.09(0.06,0.14) |  | 7.67(4.80,11.04) | 0.08(0.05,0.11) |  | -20.30(-51.53,23.11) | -0.52(-0.68,-0.35) |
| Armenia | 0.14(0.07,0.31) | 0.01(0.01,0.03) |  | 0.06(0.03,0.10) | 0.01(0.01,0.02) |  | -58.72(-84.33,-6.46) | -0.31(-0.73,0.11) |
| Australia | 0.99(0.64,1.51) | 0.03(0.02,0.04) |  | 0.75(0.34,1.19) | 0.02(0.01,0.02) |  | -23.58(-68.84,34.22) | -1.65(-2.07,-1.23) |
| Austria | 0.37(0.24,0.55) | 0.03(0.02,0.04) |  | 0.26(0.13,0.38) | 0.02(0.01,0.03) |  | -29.67(-67.61,19.06) | -1.00(-1.40,-0.59) |
| Azerbaijan | 0.35(0.14,0.78) | 0.01(0.01,0.03) |  | 0.27(0.12,0.55) | 0.01(0.00,0.02) |  | -22.64(-70.32,108.24) | -1.51(-1.96,-1.06) |
| Bahamas | 0.07(0.04,0.10) | 0.08(0.05,0.12) |  | 0.05(0.03,0.08) | 0.07(0.04,0.10) |  | -18.87(-52.95,23.97) | -1.04(-1.34,-0.73) |
| Bahrain | 0.03(0.01,0.07) | 0.02(0.01,0.04) |  | 0.03(0.01,0.06) | 0.01(0.00,0.02) |  | -16.46(-77.45,106.29) | -3.14(-3.76,-2.52) |
| Bangladesh | 24.41(6.56,65.63) | 0.05(0.01,0.13) |  | 19.02(6.38,42.76) | 0.04(0.01,0.09) |  | -22.07(-83.08,255.24) | -1.33(-1.68,-0.98) |
| Barbados | 0.13(0.09,0.19) | 0.21(0.15,0.30) |  | 0.06(0.04,0.09) | 0.13(0.08,0.19) |  | -54.38(-73.81,-26.98) | -0.82(-1.32,-0.33) |
| Belarus | 0.81(0.51,1.24) | 0.03(0.02,0.05) |  | 0.38(0.13,0.78) | 0.02(0.01,0.05) |  | -52.69(-85.37,2.56) | -0.26(-1.75,1.25) |
| Belgium | 0.50(0.29,0.79) | 0.03(0.02,0.04) |  | 0.56(0.23,0.89) | 0.03(0.01,0.05) |  | 11.99(-62.60,127.51) | -0.16(-0.72,0.40) |
| Belize | 0.04(0.02,0.08) | 0.05(0.03,0.10) |  | 0.04(0.03,0.06) | 0.04(0.02,0.05) |  | 5.29(-57.00,72.65) | -0.58(-0.85,-0.32) |
| Benin | 15.58(6.21,26.48) | 0.64(0.26,1.09) |  | 36.80(19.10,56.89) | 0.61(0.31,0.94) |  | 136.22(32.86,350.17) | 0.03(-0.16,0.22) |
| Bermuda | 0.01(0.01,0.02) | 0.09(0.05,0.14) |  | 0.00(0.00,0.01) | 0.06(0.02,0.10) |  | -55.27(-85.42,29.97) | -1.19(-1.53,-0.86) |
| Bhutan | 0.13(0.03,0.34) | 0.05(0.01,0.13) |  | 0.08(0.03,0.20) | 0.04(0.01,0.11) |  | -39.07(-89.24,346.16) | -1.43(-1.91,-0.95) |
| Bolivia (Plurinational State of) | 4.39(2.11,8.18) | 0.16(0.08,0.30) |  | 3.42(1.52,6.00) | 0.10(0.04,0.17) |  | -22.03(-75.48,80.93) | -2.50(-2.93,-2.07) |
| Bosnia and Herzegovina | 0.15(0.05,0.35) | 0.01(0.00,0.03) |  | 0.05(0.02,0.12) | 0.01(0.00,0.02) |  | -65.30(-91.56,19.11) | -1.26(-1.75,-0.76) |
| Botswana | 0.42(0.21,0.79) | 0.07(0.04,0.13) |  | 0.98(0.43,1.74) | 0.14(0.06,0.25) |  | 131.56(-4.21,401.47) | 2.55(2.30,2.81) |
| Brazil | 56.70(39.91,81.49) | 0.11(0.08,0.16) |  | 36.55(19.75,50.46) | 0.08(0.04,0.10) |  | -35.54(-69.59,-8.95) | -0.86(-1.42,-0.30) |
| Brunei Darussalam | 0.06(0.03,0.10) | 0.06(0.03,0.12) |  | 0.03(0.02,0.06) | 0.04(0.02,0.06) |  | -41.77(-74.95,26.25) | -1.69(-2.58,-0.80) |
| Bulgaria | 0.19(0.09,0.39) | 0.01(0.01,0.02) |  | 0.10(0.04,0.17) | 0.01(0.00,0.02) |  | -48.74(-84.25,40.59) | -0.54(-1.01,-0.07) |
| Burkina Faso | 32.12(13.31,53.14) | 0.68(0.28,1.13) |  | 64.65(33.93,100.26) | 0.62(0.33,0.97) |  | 101.28(22.55,261.36) | 0.09(-0.17,0.35) |
| Burundi | 34.63(13.46,61.46) | 1.32(0.51,2.34) |  | 31.76(15.84,56.12) | 0.54(0.27,0.96) |  | -8.31(-44.48,78.85) | -2.60(-2.80,-2.40) |
| Cabo Verde | 0.19(0.09,0.31) | 0.12(0.06,0.19) |  | 0.46(0.18,0.73) | 0.32(0.13,0.51) |  | 149.80(-16.86,382.67) | 1.73(0.76,2.71) |
| Cambodia | 1.40(0.27,4.32) | 0.03(0.01,0.09) |  | 0.94(0.38,1.85) | 0.02(0.01,0.04) |  | -33.37(-81.10,185.88) | -2.15(-2.39,-1.92) |
| Cameroon | 31.44(14.29,50.77) | 0.64(0.29,1.04) |  | 90.55(50.49,133.98) | 0.67(0.37,0.99) |  | 188.00(75.76,422.92) | 0.52(0.27,0.77) |
| Canada | 1.55(0.96,2.38) | 0.03(0.02,0.04) |  | 1.08(0.51,1.83) | 0.02(0.01,0.03) |  | -30.24(-69.50,18.31) | -1.83(-2.15,-1.51) |
| Central African Republic | 6.63(2.34,11.91) | 0.54(0.19,0.97) |  | 9.38(4.19,15.55) | 0.41(0.18,0.68) |  | 41.41(-13.30,165.43) | -0.76(-0.88,-0.63) |
| Chad | 15.50(6.42,26.24) | 0.53(0.22,0.90) |  | 55.67(28.84,87.87) | 0.62(0.32,0.97) |  | 259.21(117.78,539.78) | 0.91(0.73,1.08) |
| Chile | 3.10(1.57,5.17) | 0.08(0.04,0.13) |  | 1.87(1.05,2.70) | 0.05(0.03,0.07) |  | -39.52(-73.57,33.66) | -0.94(-1.24,-0.63) |
| China | 131.98(55.24,196.90) | 0.04(0.02,0.06) |  | 30.50(16.33,51.70) | 0.01(0.01,0.02) |  | -76.89(-89.48,-39.55) | -5.47(-6.03,-4.92) |
| Colombia | 12.06(7.64,18.54) | 0.10(0.07,0.16) |  | 8.37(3.99,14.70) | 0.08(0.04,0.14) |  | -30.59(-69.39,18.77) | -0.21(-0.58,0.15) |
| Comoros | 1.94(0.79,3.23) | 0.91(0.37,1.52) |  | 1.57(0.90,2.41) | 0.66(0.37,1.00) |  | -19.12(-60.16,66.28) | -1.33(-1.72,-0.95) |
| Congo | 4.15(1.64,6.93) | 0.39(0.16,0.66) |  | 4.49(2.46,6.70) | 0.23(0.13,0.35) |  | 8.25(-33.68,111.65) | -1.71(-1.95,-1.47) |
| Cook Islands | 0.00(0.00,0.00) | 0.01(0.00,0.02) |  | 0.00(0.00,0.00) | 0.01(0.00,0.02) |  | -31.73(-88.21,101.32) | -0.40(-0.76,-0.03) |
| Costa Rica | 0.95(0.52,1.52) | 0.08(0.05,0.14) |  | 0.69(0.26,1.30) | 0.07(0.03,0.13) |  | -27.41(-72.50,116.92) | -0.92(-1.17,-0.68) |
| Croatia | 36.22(17.38,55.71) | 0.64(0.30,0.98) |  | 66.14(35.03,102.83) | 0.57(0.30,0.89) |  | -48.43(-86.11,59.83) | 0.07(-0.15,0.29) |
| Cuba | 0.30(0.14,0.52) | 0.03(0.01,0.05) |  | 0.16(0.05,0.27) | 0.03(0.01,0.05) |  | -75.24(-88.01,-46.36) | 0.01(-0.57,0.60) |
| Cyprus | 4.31(2.63,6.82) | 0.17(0.11,0.27) |  | 1.07(0.59,1.81) | 0.06(0.03,0.10) |  | -45.03(-74.80,29.70) | -0.98(-1.79,-0.16) |
| Czechia | 0.09(0.05,0.17) | 0.05(0.02,0.09) |  | 0.05(0.03,0.08) | 0.02(0.01,0.04) |  | -55.21(-89.42,17.20) | -2.22(-2.98,-1.46) |
| C么te d'Ivoire | 0.60(0.39,0.97) | 0.03(0.02,0.04) |  | 0.27(0.07,0.57) | 0.02(0.00,0.03) |  | 82.62(9.66,203.21) | -1.79(-2.16,-1.42) |
| Democratic People's Republic of Korea | 1.56(0.65,3.49) | 0.03(0.01,0.06) |  | 0.86(0.31,1.81) | 0.02(0.01,0.04) |  | -45.07(-79.81,38.46) | -1.57(-1.75,-1.40) |
| Democratic Republic of the Congo | 87.17(30.96,156.88) | 0.49(0.17,0.89) |  | 80.95(40.35,127.65) | 0.21(0.11,0.34) |  | -7.14(-44.08,111.96) | -2.29(-2.50,-2.09) |
| Denmark | 0.37(0.24,0.56) | 0.04(0.03,0.06) |  | 0.17(0.08,0.27) | 0.02(0.01,0.03) |  | -54.39(-81.30,-25.70) | -2.76(-3.11,-2.42) |
| Djibouti | 1.23(0.60,1.95) | 0.71(0.35,1.12) |  | 2.49(1.37,4.04) | 0.60(0.33,0.98) |  | 102.74(16.10,260.23) | -0.49(-0.87,-0.12) |
| Dominica | 0.02(0.01,0.03) | 0.06(0.03,0.12) |  | 0.01(0.00,0.02) | 0.07(0.03,0.13) |  | -37.93(-70.96,47.73) | 0.82(0.55,1.10) |
| Dominican Republic | 1.19(0.63,2.50) | 0.04(0.02,0.09) |  | 1.65(0.71,3.38) | 0.06(0.02,0.12) |  | 38.91(-53.89,286.28) | 0.86(0.45,1.28) |
| Ecuador | 2.66(1.78,3.86) | 0.07(0.05,0.10) |  | 3.14(1.34,4.98) | 0.06(0.03,0.10) |  | 18.00(-55.60,94.39) | -0.18(-0.52,0.16) |
| Egypt | 7.82(1.77,27.47) | 0.04(0.01,0.12) |  | 5.17(0.63,20.65) | 0.01(0.00,0.06) |  | -33.84(-90.02,72.07) | -2.92(-4.25,-1.58) |
| El Salvador | 1.03(0.61,1.73) | 0.05(0.03,0.08) |  | 0.60(0.33,0.98) | 0.03(0.02,0.05) |  | -41.04(-74.84,5.90) | -1.36(-1.63,-1.09) |
| Equatorial Guinea | 0.99(0.38,1.75) | 0.50(0.19,0.89) |  | 1.46(0.61,2.97) | 0.25(0.10,0.51) |  | 47.66(-32.22,279.76) | -3.01(-3.34,-2.67) |
| Eritrea | 14.11(6.05,22.73) | 0.89(0.38,1.43) |  | 17.69(9.29,29.18) | 0.70(0.37,1.16) |  | 25.38(-28.03,143.49) | -0.83(-0.98,-0.69) |
| Estonia | 0.15(0.09,0.29) | 0.04(0.03,0.08) |  | 0.05(0.01,0.10) | 0.02(0.01,0.05) |  | -67.42(-93.91,-7.27) | -1.82(-2.11,-1.53) |
| Eswatini | 0.36(0.16,0.74) | 0.09(0.04,0.19) |  | 0.54(0.23,1.03) | 0.13(0.06,0.25) |  | 51.21(-34.50,256.30) | 1.72(1.41,2.03) |
| Ethiopia | 248.68(76.74,488.73) | 1.02(0.31,2.01) |  | 214.09(113.49,335.35) | 0.48(0.26,0.76) |  | -13.91(-53.01,88.46) | -2.67(-2.86,-2.47) |
| Fiji | 0.07(0.03,0.14) | 0.03(0.01,0.05) |  | 0.15(0.06,0.32) | 0.05(0.02,0.12) |  | 107.22(-23.42,529.54) | 2.84(2.48,3.20) |
| Finland | 0.31(0.18,0.50) | 0.03(0.02,0.05) |  | 0.13(0.05,0.22) | 0.01(0.01,0.03) |  | -59.43(-85.13,-24.07) | -2.63(-2.99,-2.27) |
| France | 3.07(1.97,4.66) | 0.03(0.02,0.04) |  | 2.67(0.85,5.50) | 0.02(0.01,0.05) |  | -13.05(-70.18,86.05) | -0.76(-1.10,-0.41) |
| Gabon | 1.29(0.58,2.05) | 0.32(0.14,0.50) |  | 1.66(0.88,2.66) | 0.26(0.14,0.42) |  | 28.65(-22.51,122.51) | -0.19(-0.37,-0.01) |
| Gambia | 2.60(1.24,4.35) | 0.56(0.27,0.94) |  | 4.81(2.58,7.92) | 0.48(0.26,0.80) |  | 84.57(1.51,247.38) | -0.57(-0.87,-0.27) |
| Georgia | 1.35(0.38,2.44) | 0.10(0.03,0.18) |  | 0.20(0.09,0.31) | 0.03(0.01,0.04) |  | -85.44(-95.25,-33.17) | -4.19(-5.28,-3.08) |
| Germany | 2.65(1.59,4.32) | 0.02(0.01,0.03) |  | 1.71(0.64,2.99) | 0.01(0.01,0.03) |  | -35.71(-78.06,19.32) | -0.94(-1.56,-0.32) |
| Ghana | 73.89(23.27,132.08) | 1.10(0.35,1.97) |  | 51.34(31.12,89.79) | 0.40(0.24,0.70) |  | -30.51(-65.21,146.93) | -4.24(-4.93,-3.53) |
| Greece | 0.30(0.11,0.55) | 0.01(0.01,0.03) |  | 0.21(0.12,0.30) | 0.01(0.01,0.02) |  | -30.95(-67.84,98.49) | 0.08(-0.35,0.52) |
| Greenland | 0.01(0.00,0.03) | 0.10(0.01,0.22) |  | 0.00(0.00,0.00) | 0.02(0.01,0.04) |  | -83.81(-96.64,17.56) | -4.17(-4.81,-3.52) |
| Grenada | 0.05(0.03,0.09) | 0.16(0.09,0.26) |  | 0.03(0.02,0.04) | 0.12(0.07,0.19) |  | -49.84(-71.31,-22.40) | -0.35(-0.53,-0.18) |
| Guam | 0.01(0.00,0.01) | 0.02(0.01,0.03) |  | 0.01(0.01,0.02) | 0.04(0.02,0.05) |  | 92.53(-13.67,404.72) | 5.00(4.13,5.88) |
| Guatemala | 3.74(2.13,7.62) | 0.09(0.05,0.19) |  | 2.30(1.42,3.40) | 0.05(0.03,0.07) |  | -38.37(-75.37,12.58) | -1.58(-1.80,-1.36) |
| Guinea | 19.14(8.09,31.78) | 0.70(0.29,1.15) |  | 27.37(15.23,46.96) | 0.45(0.25,0.78) |  | 42.98(-15.55,175.72) | -0.83(-1.07,-0.58) |
| Guinea-Bissau | 3.83(1.52,6.55) | 0.79(0.32,1.36) |  | 4.44(2.26,7.26) | 0.49(0.25,0.81) |  | 16.03(-29.33,133.77) | -1.21(-1.56,-0.85) |
| Guyana | 0.24(0.13,0.40) | 0.08(0.05,0.14) |  | 0.08(0.05,0.16) | 0.04(0.02,0.08) |  | -64.57(-83.67,-1.87) | -0.18(-1.02,0.67) |
| Haiti | 6.36(1.16,21.10) | 0.23(0.04,0.78) |  | 8.14(2.20,21.21) | 0.19(0.05,0.49) |  | 27.88(-48.45,280.90) | -0.44(-0.61,-0.27) |
| Honduras | 1.10(0.51,2.28) | 0.05(0.02,0.10) |  | 0.91(0.44,1.64) | 0.03(0.01,0.05) |  | -16.84(-65.61,100.95) | -2.28(-2.72,-1.85) |
| Hungary | 0.54(0.31,0.88) | 0.03(0.01,0.04) |  | 0.27(0.07,0.50) | 0.02(0.01,0.04) |  | -49.87(-86.64,47.30) | -0.54(-1.07,0.00) |
| Iceland | 0.01(0.01,0.02) | 0.02(0.01,0.03) |  | 0.01(0.00,0.01) | 0.01(0.00,0.01) |  | -56.71(-79.67,-19.20) | -2.02(-2.68,-1.36) |
| India | 178.36(56.16,350.82) | 0.05(0.02,0.11) |  | 89.61(58.28,137.95) | 0.02(0.02,0.04) |  | -49.76(-81.56,67.55) | -2.91(-3.07,-2.74) |
| Indonesia | 9.03(2.28,19.82) | 0.01(0.00,0.03) |  | 9.70(4.78,16.25) | 0.01(0.01,0.02) |  | 7.41(-48.02,170.18) | -1.24(-1.75,-0.73) |
| Iran (Islamic Republic of) | 6.28(2.89,15.87) | 0.02(0.01,0.06) |  | 3.66(1.63,6.67) | 0.02(0.01,0.03) |  | -41.77(-88.48,89.09) | -1.16(-1.56,-0.75) |
| Iraq | 10.08(1.79,25.13) | 0.12(0.02,0.31) |  | 4.75(1.60,10.33) | 0.04(0.01,0.08) |  | -52.92(-83.23,76.84) | -4.20(-4.54,-3.85) |
| Ireland | 0.21(0.13,0.34) | 0.02(0.01,0.03) |  | 0.18(0.07,0.31) | 0.02(0.01,0.03) |  | -11.15(-65.05,86.15) | -1.14(-1.68,-0.60) |
| Israel | 1.05(0.61,1.71) | 0.07(0.04,0.11) |  | 1.24(0.72,1.78) | 0.05(0.03,0.07) |  | 18.09(-41.45,98.41) | -1.05(-1.64,-0.45) |
| Italy | 2.62(1.81,4.32) | 0.03(0.02,0.05) |  | 1.90(0.67,3.58) | 0.03(0.01,0.05) |  | -27.35(-79.45,71.81) | -0.08(-0.50,0.35) |
| Jamaica | 0.57(0.32,1.02) | 0.07(0.04,0.12) |  | 0.25(0.14,0.44) | 0.04(0.02,0.08) |  | -55.73(-79.16,-19.95) | -1.13(-1.44,-0.82) |
| Japan | 4.14(1.75,7.17) | 0.02(0.01,0.03) |  | 2.68(1.15,3.70) | 0.02(0.01,0.02) |  | -35.18(-74.63,90.62) | -0.17(-0.60,0.27) |
| Jordan | 1.16(0.54,2.33) | 0.07(0.03,0.14) |  | 1.69(0.87,3.09) | 0.05(0.02,0.08) |  | 45.90(-41.04,261.18) | -2.11(-2.58,-1.64) |
| Kazakhstan | 1.63(0.80,3.11) | 0.03(0.02,0.06) |  | 0.73(0.34,1.31) | 0.01(0.01,0.02) |  | -55.20(-85.09,2.99) | -3.29(-3.84,-2.74) |
| Kenya | 31.38(16.48,46.86) | 0.28(0.15,0.42) |  | 43.23(27.06,58.69) | 0.23(0.14,0.31) |  | 37.77(-5.15,109.60) | 0.43(-0.03,0.88) |
| Kiribati | 0.00(0.00,0.00) | 0.00(0.00,0.01) |  | 0.00(0.00,0.00) | 0.00(0.00,0.01) |  | 26.63(-64.47,239.14) | -0.93(-1.29,-0.57) |
| Kuwait | 0.33(0.19,0.57) | 0.06(0.03,0.10) |  | 0.16(0.08,0.26) | 0.02(0.01,0.03) |  | -51.42(-76.77,-11.41) | -3.25(-3.83,-2.65) |
| Kyrgyzstan | 0.40(0.22,0.79) | 0.02(0.01,0.05) |  | 0.30(0.14,0.56) | 0.01(0.01,0.02) |  | -25.52(-75.93,78.12) | -2.22(-2.87,-1.57) |
| Lao People's Democratic Republic | 0.44(0.06,1.49) | 0.02(0.00,0.08) |  | 0.54(0.20,1.13) | 0.02(0.01,0.05) |  | 22.00(-66.47,615.96) | -2.38(-2.91,-1.84) |
| Latvia | 0.19(0.10,0.36) | 0.03(0.02,0.06) |  | 0.06(0.01,0.11) | 0.02(0.01,0.04) |  | -70.58(-94.43,-14.58) | -1.40(-1.70,-1.09) |
| Lebanon | 0.64(0.29,1.30) | 0.06(0.03,0.12) |  | 0.44(0.17,0.88) | 0.03(0.01,0.07) |  | -30.90(-77.82,111.69) | -1.86(-2.12,-1.59) |
| Lesotho | 0.44(0.21,0.82) | 0.06(0.03,0.12) |  | 0.79(0.33,1.51) | 0.12(0.05,0.24) |  | 78.70(-28.46,283.01) | 2.83(2.56,3.11) |
| Liberia | 10.00(4.34,16.81) | 0.89(0.38,1.49) |  | 11.95(6.15,17.96) | 0.55(0.28,0.82) |  | 19.47(-32.03,132.29) | -1.70(-2.20,-1.19) |
| Libya | 1.42(0.50,3.30) | 0.08(0.03,0.18) |  | 1.49(0.51,3.21) | 0.10(0.03,0.22) |  | 5.34(-59.48,194.77) | 1.17(0.83,1.50) |
| Lithuania | 0.26(0.14,0.44) | 0.03(0.02,0.05) |  | 0.10(0.03,0.22) | 0.02(0.01,0.05) |  | -62.55(-91.45,31.27) | -1.19(-1.61,-0.77) |
| Luxembourg | 0.02(0.01,0.04) | 0.04(0.01,0.06) |  | 0.03(0.02,0.05) | 0.03(0.02,0.04) |  | 25.35(-36.67,181.57) | -1.35(-1.71,-0.99) |
| Madagascar | 44.06(21.64,65.40) | 0.81(0.40,1.20) |  | 59.07(36.04,86.57) | 0.50(0.31,0.74) |  | 34.07(-17.03,125.52) | -1.04(-1.22,-0.86) |
| Malawi | 96.84(43.81,159.92) | 2.13(0.96,3.51) |  | 105.99(48.95,208.67) | 1.30(0.60,2.57) |  | 9.45(-37.81,109.38) | -1.25(-1.52,-0.98) |
| Malaysia | 1.19(0.52,2.56) | 0.02(0.01,0.04) |  | 0.77(0.24,1.76) | 0.01(0.00,0.02) |  | -35.16(-82.03,70.36) | -2.45(-3.11,-1.80) |
| Maldives | 0.06(0.02,0.15) | 0.05(0.02,0.15) |  | 0.02(0.01,0.04) | 0.02(0.01,0.04) |  | -63.18(-90.95,44.82) | -3.17(-3.39,-2.95) |
| Mali | 20.95(9.59,34.19) | 0.51(0.23,0.83) |  | 37.77(21.31,60.36) | 0.33(0.18,0.52) |  | 80.30(-0.08,241.54) | -1.08(-1.32,-0.84) |
| Malta | 0.02(0.01,0.04) | 0.03(0.01,0.04) |  | 0.02(0.01,0.04) | 0.04(0.02,0.07) |  | 1.04(-45.83,188.59) | 0.20(-0.43,0.83) |
| Marshall Islands | 0.00(0.00,0.00) | 0.01(0.00,0.02) |  | 0.00(0.00,0.01) | 0.03(0.01,0.06) |  | 102.92(-26.90,442.21) | 2.57(2.10,3.04) |
| Mauritania | 4.18(2.08,6.77) | 0.45(0.23,0.73) |  | 6.84(4.02,10.24) | 0.37(0.22,0.55) |  | 63.64(0.79,193.59) | -0.84(-1.17,-0.51) |
| Mauritius | 0.03(0.02,0.05) | 0.01(0.01,0.01) |  | 0.02(0.01,0.03) | 0.01(0.00,0.01) |  | -44.77(-69.09,-15.51) | -0.54(-0.79,-0.28) |
| Mexico | 22.31(14.70,37.19) | 0.07(0.04,0.11) |  | 16.02(8.20,23.00) | 0.05(0.03,0.07) |  | -28.22(-70.24,11.50) | -0.58(-0.94,-0.22) |
| Micronesia (Federated States of) | 0.00(0.00,0.01) | 0.01(0.00,0.02) |  | 0.01(0.00,0.02) | 0.02(0.01,0.05) |  | 37.63(-59.20,374.54) | 2.70(2.47,2.93) |
| Monaco | 0.00(0.00,0.00) | 0.01(0.00,0.02) |  | 0.00(0.00,0.00) | 0.01(0.00,0.02) |  | 63.35(-36.24,392.48) | 0.07(-0.51,0.66) |
| Mongolia | 0.27(0.07,0.79) | 0.03(0.01,0.09) |  | 0.37(0.15,0.72) | 0.03(0.01,0.07) |  | 34.11(-67.50,476.76) | -0.04(-0.33,0.26) |
| Montenegro | 0.08(0.05,0.13) | 0.05(0.03,0.08) |  | 0.02(0.01,0.05) | 0.02(0.01,0.04) |  | -71.08(-87.89,-38.64) | -2.74(-3.17,-2.30) |
| Morocco | 5.86(2.10,13.46) | 0.06(0.02,0.14) |  | 3.71(1.63,6.80) | 0.04(0.02,0.07) |  | -36.66(-78.77,71.92) | -1.20(-1.39,-1.01) |
| Mozambique | 11.02(4.21,25.95) | 0.18(0.07,0.42) |  | 11.84(5.04,29.64) | 0.08(0.04,0.21) |  | 7.44(-44.57,115.97) | -2.05(-2.31,-1.80) |
| Myanmar | 5.26(0.80,17.31) | 0.04(0.01,0.12) |  | 3.18(1.33,6.33) | 0.02(0.01,0.04) |  | -39.66(-82.57,156.00) | -2.48(-2.76,-2.20) |
| Namibia | 0.69(0.35,1.21) | 0.12(0.06,0.20) |  | 1.52(0.77,2.62) | 0.18(0.09,0.32) |  | 120.23(6.63,364.10) | 2.12(1.87,2.36) |
| Nauru | 0.00(0.00,0.00) | 0.02(0.01,0.05) |  | 0.00(0.00,0.00) | 0.04(0.01,0.10) |  | 82.70(-36.10,360.88) | 2.16(1.76,2.56) |
| Nepal | 3.38(1.02,7.95) | 0.04(0.01,0.09) |  | 3.15(0.96,7.61) | 0.03(0.01,0.08) |  | -6.81(-80.18,291.50) | -1.11(-1.32,-0.90) |
| Netherlands | 1.29(0.82,1.93) | 0.05(0.03,0.07) |  | 0.87(0.40,1.29) | 0.03(0.01,0.05) |  | -32.50(-71.45,7.06) | -1.20(-1.51,-0.88) |
| New Zealand | 0.32(0.22,0.46) | 0.04(0.03,0.06) |  | 0.23(0.15,0.33) | 0.02(0.02,0.03) |  | -26.60(-58.18,7.95) | -2.17(-3.22,-1.11) |
| Nicaragua | 1.48(0.84,2.62) | 0.08(0.05,0.14) |  | 0.96(0.60,1.51) | 0.05(0.03,0.08) |  | -35.05(-68.20,28.32) | -1.65(-1.93,-1.37) |
| Niger | 35.37(13.17,64.81) | 0.87(0.32,1.60) |  | 60.41(27.55,99.30) | 0.47(0.22,0.78) |  | 70.83(-2.53,246.15) | -1.79(-2.00,-1.59) |
| Nigeria | 290.42(144.67,433.81) | 0.74(0.37,1.11) |  | 620.43(312.21,882.17) | 0.61(0.31,0.87) |  | 113.63(56.24,213.29) | -0.33(-0.48,-0.18) |
| Niue | 0.00(0.00,0.00) | 0.02(0.01,0.04) |  | 0.00(0.00,0.00) | 0.05(0.01,0.11) |  | 23.70(-59.25,266.99) | 2.46(2.22,2.70) |
| North Macedonia | 0.04(0.02,0.09) | 0.01(0.00,0.02) |  | 0.02(0.01,0.04) | 0.01(0.00,0.01) |  | -46.22(-89.09,72.11) | -0.20(-0.82,0.43) |
| Northern Mariana Islands | 0.00(0.00,0.00) | 0.01(0.00,0.01) |  | 0.00(0.00,0.00) | 0.01(0.00,0.02) |  | 66.59(-42.90,638.81) | 3.40(2.51,4.31) |
| Norway | 0.13(0.09,0.22) | 0.02(0.01,0.03) |  | 0.08(0.03,0.12) | 0.01(0.00,0.01) |  | -41.85(-79.74,-13.56) | -2.15(-2.69,-1.61) |
| Oman | 0.39(0.14,0.83) | 0.05(0.02,0.10) |  | 0.43(0.14,0.82) | 0.04(0.01,0.07) |  | 11.50(-60.43,204.47) | -0.67(-0.94,-0.39) |
| Pakistan | 67.39(26.90,124.03) | 0.14(0.05,0.25) |  | 164.15(66.57,309.79) | 0.19(0.08,0.36) |  | 143.57(21.81,368.39) | 1.20(1.03,1.37) |
| Palau | 0.00(0.00,0.00) | 0.02(0.01,0.03) |  | 0.00(0.00,0.00) | 0.02(0.01,0.03) |  | -26.82(-67.00,57.42) | 0.32(0.15,0.50) |
| Palestine | 0.40(0.16,0.87) | 0.04(0.02,0.09) |  | 0.48(0.16,1.00) | 0.03(0.01,0.05) |  | 21.01(-68.99,241.36) | -1.25(-1.63,-0.87) |
| Panama | 0.91(0.59,1.39) | 0.11(0.07,0.17) |  | 1.18(0.76,1.63) | 0.10(0.07,0.14) |  | 29.48(-22.39,90.31) | -0.31(-0.47,-0.15) |
| Papua New Guinea | 0.38(0.10,0.99) | 0.02(0.01,0.06) |  | 1.58(0.38,3.64) | 0.04(0.01,0.09) |  | 311.40(71.73,986.28) | 1.99(1.58,2.40) |
| Paraguay | 0.92(0.50,1.58) | 0.06(0.03,0.09) |  | 0.96(0.43,1.70) | 0.05(0.02,0.08) |  | 4.54(-54.63,123.18) | -0.38(-0.79,0.04) |
| Peru | 10.59(5.65,18.51) | 0.13(0.07,0.22) |  | 6.55(3.27,11.30) | 0.07(0.03,0.12) |  | -38.16(-76.25,41.72) | -2.15(-2.33,-1.97) |
| Philippines | 9.11(3.67,15.14) | 0.04(0.01,0.06) |  | 7.24(4.56,10.47) | 0.02(0.01,0.03) |  | -20.47(-57.27,59.01) | -1.39(-1.59,-1.19) |
| Poland | 1.78(0.55,3.59) | 0.02(0.01,0.04) |  | 1.11(0.42,1.59) | 0.02(0.01,0.03) |  | -37.70(-83.94,142.57) | -0.04(-0.88,0.80) |
| Portugal | 0.85(0.49,1.72) | 0.04(0.02,0.08) |  | 0.38(0.15,0.64) | 0.03(0.01,0.05) |  | -55.08(-88.98,-16.23) | -1.54(-1.92,-1.16) |
| Puerto Rico | 1.02(0.55,1.60) | 0.10(0.05,0.16) |  | 0.23(0.10,0.34) | 0.05(0.02,0.08) |  | -77.57(-90.70,-57.47) | -1.87(-2.09,-1.65) |
| Qatar | 0.02(0.01,0.04) | 0.01(0.00,0.03) |  | 0.04(0.01,0.10) | 0.01(0.00,0.02) |  | 155.77(-6.27,567.46) | -1.64(-2.08,-1.19) |
| Republic of Korea | 2.25(0.71,4.78) | 0.02(0.01,0.04) |  | 0.49(0.20,0.97) | 0.01(0.00,0.02) |  | -78.10(-93.80,-23.60) | -2.98(-3.30,-2.66) |
| Republic of Moldova | 1.92(0.94,3.75) | 0.16(0.08,0.30) |  | 0.34(0.18,0.53) | 0.06(0.03,0.10) |  | -82.40(-93.00,-59.63) | -1.95(-2.51,-1.38) |
| Romania | 2.83(1.11,6.38) | 0.05(0.02,0.11) |  | 1.15(0.63,1.80) | 0.04(0.02,0.06) |  | -59.37(-87.31,12.28) | -0.79(-1.19,-0.39) |
| Russian Federation | 18.62(11.06,27.88) | 0.05(0.03,0.08) |  | 6.14(2.72,8.81) | 0.02(0.01,0.03) |  | -67.03(-86.54,-48.04) | -2.27(-2.61,-1.92) |
| Rwanda | 47.95(20.03,76.97) | 1.41(0.59,2.27) |  | 30.76(17.64,48.81) | 0.62(0.35,0.98) |  | -35.85(-63.06,25.69) | -3.27(-3.56,-2.97) |
| Saint Kitts and Nevis | 0.01(0.00,0.01) | 0.04(0.02,0.07) |  | 0.00(0.00,0.01) | 0.04(0.02,0.07) |  | -31.83(-64.86,65.70) | 0.77(0.42,1.12) |
| Saint Lucia | 0.04(0.02,0.06) | 0.07(0.05,0.12) |  | 0.02(0.01,0.03) | 0.07(0.04,0.10) |  | -46.02(-69.35,-5.76) | -0.22(-0.51,0.06) |
| Saint Vincent and the Grenadines | 0.03(0.01,0.06) | 0.08(0.02,0.15) |  | 0.02(0.01,0.03) | 0.07(0.05,0.11) |  | -43.02(-70.94,144.37) | -0.28(-0.56,-0.01) |
| Samoa | 0.01(0.00,0.02) | 0.01(0.00,0.03) |  | 0.01(0.00,0.03) | 0.01(0.01,0.03) |  | 31.27(-62.76,315.70) | 0.21(-0.01,0.43) |
| San Marino | 0.00(0.00,0.00) | 0.05(0.02,0.10) |  | 0.00(0.00,0.00) | 0.04(0.01,0.08) |  | -13.04(-70.11,136.32) | -0.65(-0.99,-0.32) |
| Sao Tome and Principe | 0.41(0.18,0.67) | 0.72(0.32,1.18) |  | 0.22(0.13,0.34) | 0.28(0.16,0.44) |  | -46.88(-71.19,14.45) | -2.49(-2.84,-2.14) |
| Saudi Arabia | 3.90(1.82,7.52) | 0.06(0.03,0.11) |  | 2.25(0.59,4.40) | 0.03(0.01,0.06) |  | -42.23(-85.03,49.39) | -2.35(-2.54,-2.16) |
| Senegal | 24.70(10.85,38.74) | 0.68(0.30,1.06) |  | 26.22(16.06,39.19) | 0.41(0.25,0.62) |  | 6.14(-37.12,92.28) | -1.51(-1.78,-1.24) |
| Serbia | 0.50(0.20,1.06) | 0.02(0.01,0.05) |  | 0.13(0.04,0.29) | 0.01(0.00,0.02) |  | -73.49(-93.04,-24.28) | -2.90(-3.17,-2.62) |
| Seychelles | 0.00(0.00,0.00) | 0.00(0.00,0.00) |  | 0.00(0.00,0.00) | 0.00(0.00,0.00) |  | 32.66(-67.55,608.41) | 0.59(0.15,1.03) |
| Sierra Leone | 15.15(6.29,25.70) | 0.84(0.35,1.42) |  | 21.99(11.67,33.90) | 0.62(0.33,0.95) |  | 45.16(-15.89,175.59) | -0.96(-1.15,-0.76) |
| Singapore | 0.17(0.09,0.33) | 0.03(0.01,0.05) |  | 0.14(0.04,0.24) | 0.02(0.01,0.03) |  | -19.22(-80.72,108.15) | -1.29(-2.15,-0.44) |
| Slovakia | 0.20(0.09,0.39) | 0.01(0.01,0.03) |  | 0.16(0.06,0.34) | 0.02(0.01,0.04) |  | -20.72(-74.65,133.77) | 0.59(-0.13,1.31) |
| Slovenia | 0.07(0.04,0.12) | 0.02(0.01,0.03) |  | 0.02(0.01,0.03) | 0.01(0.00,0.01) |  | -73.44(-93.85,-48.24) | -2.81(-3.55,-2.06) |
| Solomon Islands | 0.02(0.01,0.04) | 0.01(0.00,0.03) |  | 0.06(0.02,0.13) | 0.02(0.01,0.05) |  | 218.73(14.27,844.38) | 2.00(1.54,2.45) |
| Somalia | 30.14(11.48,57.02) | 0.77(0.29,1.46) |  | 56.94(24.50,99.15) | 0.55(0.24,0.96) |  | 88.92(9.07,252.31) | -0.95(-1.32,-0.58) |
| South Africa | 4.82(2.37,7.59) | 0.04(0.02,0.06) |  | 6.74(4.37,10.17) | 0.04(0.03,0.07) |  | 39.88(-19.62,182.51) | 0.48(0.12,0.85) |
| South Sudan | 24.80(11.25,41.24) | 0.94(0.43,1.57) |  | 48.99(23.86,78.61) | 1.14(0.56,1.83) |  | 97.56(28.83,222.52) | 0.71(0.02,1.41) |
| Spain | 2.90(1.73,4.67) | 0.04(0.02,0.06) |  | 1.76(0.64,3.27) | 0.03(0.01,0.05) |  | -39.28(-81.77,16.03) | -1.17(-1.50,-0.83) |
| Sri Lanka | 1.82(0.87,3.85) | 0.03(0.02,0.07) |  | 1.05(0.40,2.10) | 0.02(0.01,0.04) |  | -42.39(-81.75,43.33) | -1.30(-1.78,-0.81) |
| Sudan | 6.75(1.78,23.08) | 0.08(0.02,0.26) |  | 9.65(2.87,24.28) | 0.06(0.02,0.15) |  | 42.99(-55.89,315.18) | -0.75(-0.82,-0.69) |
| Suriname | 0.05(0.02,0.08) | 0.03(0.02,0.07) |  | 0.06(0.03,0.12) | 0.04(0.02,0.09) |  | 35.40(-41.74,246.09) | 1.39(0.85,1.93) |
| Sweden | 0.18(0.05,0.34) | 0.01(0.00,0.02) |  | 0.23(0.13,0.36) | 0.01(0.01,0.02) |  | 25.59(-48.10,260.01) | 0.57(-0.61,1.76) |
| Switzerland | 0.27(0.16,0.46) | 0.02(0.01,0.04) |  | 0.16(0.07,0.30) | 0.01(0.01,0.02) |  | -39.82(-80.82,30.70) | -2.32(-2.74,-1.90) |
| Syrian Arab Republic | 0.95(0.47,1.85) | 0.02(0.01,0.03) |  | 0.72(0.25,1.36) | 0.02(0.01,0.04) |  | -23.90(-77.45,85.00) | 0.00(-0.92,0.94) |
| Taiwan (Province of China) | 1.15(0.77,1.92) | 0.02(0.01,0.03) |  | 0.47(0.16,0.81) | 0.02(0.01,0.03) |  | -58.95(-87.63,-27.98) | -0.13(-0.61,0.36) |
| Tajikistan | 0.04(0.01,0.10) | 0.00(0.00,0.00) |  | 0.04(0.01,0.08) | 0.00(0.00,0.00) |  | -5.16(-72.02,236.75) | -2.51(-2.91,-2.12) |
| Thailand | 3.54(0.75,7.92) | 0.02(0.00,0.05) |  | 1.67(0.69,3.27) | 0.02(0.01,0.03) |  | -52.77(-81.09,56.92) | -2.09(-2.61,-1.56) |
| Timor-Leste | 0.08(0.01,0.24) | 0.02(0.00,0.07) |  | 0.08(0.03,0.18) | 0.01(0.01,0.03) |  | -3.44(-71.00,306.44) | -2.16(-2.44,-1.89) |
| Togo | 9.80(4.58,15.33) | 0.56(0.26,0.87) |  | 15.10(8.38,23.34) | 0.46(0.25,0.71) |  | 54.13(-9.21,191.84) | -0.39(-0.58,-0.19) |
| Tokelau | 0.00(0.00,0.00) | 0.01(0.01,0.03) |  | 0.00(0.00,0.00) | 0.03(0.01,0.07) |  | 38.23(-59.85,312.77) | 1.22(0.72,1.72) |
| Tonga | 0.02(0.01,0.03) | 0.04(0.02,0.08) |  | 0.02(0.01,0.05) | 0.06(0.02,0.13) |  | 39.01(-47.32,270.99) | 0.77(0.40,1.15) |
| Trinidad and Tobago | 0.41(0.25,0.65) | 0.10(0.06,0.16) |  | 0.18(0.11,0.27) | 0.07(0.04,0.10) |  | -55.49(-78.02,-23.13) | -0.86(-1.20,-0.52) |
| Tunisia | 2.63(0.93,6.19) | 0.08(0.03,0.20) |  | 1.34(0.51,2.62) | 0.05(0.02,0.09) |  | -49.17(-83.94,46.70) | -1.83(-1.96,-1.71) |
| Turkey | 42.27(19.38,77.33) | 0.21(0.09,0.38) |  | 16.53(9.83,25.45) | 0.09(0.05,0.14) |  | -60.89(-83.01,-9.40) | -2.94(-3.24,-2.64) |
| Turkmenistan | 0.18(0.07,0.48) | 0.01(0.00,0.03) |  | 0.13(0.07,0.24) | 0.01(0.00,0.02) |  | -24.64(-68.96,79.33) | -1.26(-1.71,-0.81) |
| Tuvalu | 0.00(0.00,0.00) | 0.03(0.01,0.07) |  | 0.00(0.00,0.00) | 0.02(0.01,0.05) |  | -13.34(-73.43,145.82) | -0.34(-0.61,-0.07) |
| Uganda | 118.50(66.09,173.39) | 1.41(0.78,2.06) |  | 265.78(152.02,407.02) | 1.34(0.77,2.05) |  | 124.29(47.02,252.47) | -0.15(-0.43,0.14) |
| Ukraine | 3.48(1.61,7.42) | 0.03(0.01,0.07) |  | 1.77(0.64,3.68) | 0.03(0.01,0.06) |  | -49.32(-79.93,0.90) | -0.63(-0.92,-0.33) |
| United Arab Emirates | 0.35(0.15,0.69) | 0.06(0.03,0.12) |  | 0.31(0.13,0.64) | 0.02(0.01,0.05) |  | -10.76(-63.77,89.70) | -3.03(-3.31,-2.74) |
| United Kingdom | 2.02(0.54,3.78) | 0.02(0.00,0.03) |  | 1.90(0.88,2.94) | 0.02(0.01,0.02) |  | -5.60(-67.86,180.62) | -0.20(-0.92,0.52) |
| United Republic of Tanzania | 131.24(55.54,197.09) | 1.09(0.46,1.63) |  | 172.88(98.64,261.82) | 0.71(0.40,1.07) |  | 31.72(-18.97,132.53) | -0.95(-1.16,-0.73) |
| United States of America | 19.48(13.66,26.49) | 0.03(0.02,0.05) |  | 13.37(8.47,18.37) | 0.02(0.01,0.03) |  | -31.36(-56.21,-9.09) | -1.13(-1.41,-0.85) |
| United States Virgin Islands | 0.01(0.00,0.02) | 0.03(0.01,0.07) |  | 0.00(0.00,0.01) | 0.02(0.01,0.04) |  | -77.83(-92.00,-42.76) | -1.15(-1.61,-0.69) |
| Uruguay | 0.94(0.63,1.37) | 0.12(0.08,0.17) |  | 0.59(0.36,0.87) | 0.09(0.05,0.13) |  | -37.12(-64.67,9.58) | -0.92(-1.34,-0.51) |
| Uzbekistan | 0.76(0.38,1.61) | 0.01(0.00,0.02) |  | 0.86(0.44,1.89) | 0.01(0.00,0.02) |  | 12.46(-45.44,129.23) | -0.11(-0.30,0.09) |
| Vanuatu | 0.01(0.00,0.01) | 0.01(0.00,0.02) |  | 0.02(0.01,0.04) | 0.02(0.01,0.04) |  | 213.62(14.47,761.75) | 2.10(1.57,2.64) |
| Venezuela (Bolivarian Republic of) | 6.04(2.77,10.11) | 0.09(0.04,0.14) |  | 9.40(6.22,13.25) | 0.14(0.09,0.20) |  | 55.60(-7.47,200.83) | 0.93(0.58,1.28) |
| Viet Nam | 4.97(1.30,11.83) | 0.02(0.00,0.04) |  | 7.11(1.43,17.65) | 0.03(0.01,0.07) |  | 43.02(-59.50,239.52) | 1.52(1.31,1.73) |
| Yemen | 3.66(0.95,10.72) | 0.05(0.01,0.15) |  | 5.68(1.59,14.99) | 0.04(0.01,0.11) |  | 55.33(-53.88,324.33) | -0.69(-0.82,-0.57) |
| Zambia | 40.23(15.83,62.87) | 1.07(0.42,1.67) |  | 51.87(26.75,85.59) | 0.63(0.32,1.03) |  | 28.94(-26.27,133.81) | -1.74(-1.91,-1.57) |
| Zimbabwe | 5.24(2.21,9.52) | 0.11(0.05,0.20) |  | 17.34(6.17,30.23) | 0.28(0.10,0.48) |  | 230.90(60.45,531.30) | 4.59(3.71,5.47) |
