# Supplementary material for: Global burden of childhood Burkitt lymphoma (1990–2021): epidemiological trends, regional disparities, and projections for 2035 from the Global Burden of Disease Study
Source: Front Med (Lausanne). 2025 Sep 24;12:1619750. doi: 10.3389/fmed.2025.1619750 (PMC12504260; doi:10.3389/fmed.2025.1619750)
Supplement: Supplementary file 4 [file Table_4.DOCX]

Table S4. DALYs of Burkitt lymphoma in children between 1990 and 2021 at the national level.

| location | 1990 | |  | 2021 | |  | 1990-2021 | |
| --- | --- | --- | --- | --- | --- | --- | --- | --- |
|  | DALYs cases | DALY rate |  | DALYs cases | DALY rate |  | Cases change | EAPC |
| Afghanistan | 366.99(98.02,1075.09) | 8.52(2.28,24.96) |  | 726.52(226.44,1917.09) | 5.12(1.59,13.50) |  | 97.97(-41.33,427.60) | -1.06(-1.41,-0.71) |
| Albania | 2.54(1.14,5.85) | 0.23(0.10,0.52) |  | 1.54(0.37,4.03) | 0.35(0.08,0.91) |  | -39.50(-89.61,112.92) | 1.38(0.25,2.51) |
| Algeria | 764.14(206.07,1628.42) | 7.13(1.92,15.18) |  | 639.61(243.15,1359.22) | 4.81(1.83,10.22) |  | -16.30(-66.01,130.00) | -1.15(-1.27,-1.03) |
| American Samoa | 0.11(0.04,0.25) | 0.57(0.19,1.33) |  | 0.20(0.06,0.45) | 1.39(0.44,3.19) |  | 81.37(-43.33,478.49) | 3.17(2.92,3.41) |
| Andorra | 0.47(0.18,0.93) | 4.91(1.93,9.75) |  | 0.37(0.16,0.65) | 3.62(1.58,6.43) |  | -21.14(-69.50,144.08) | -0.86(-1.35,-0.37) |
| Angola | 2768.52(776.02,5287.68) | 58.72(16.46,112.15) |  | 3450.25(1551.63,5426.70) | 22.63(10.18,35.60) |  | 24.62(-27.74,282.71) | -2.99(-3.30,-2.68) |
| Antigua and Barbuda | 0.81(0.48,1.26) | 4.47(2.63,6.93) |  | 0.71(0.44,1.03) | 4.21(2.58,6.10) |  | -12.51(-42.91,72.28) | -0.27(-0.99,0.45) |
| Argentina | 798.93(506.53,1174.90) | 7.88(5.00,11.59) |  | 639.79(400.31,919.06) | 6.28(3.93,9.03) |  | -19.92(-51.33,23.23) | -0.49(-0.66,-0.33) |
| Armenia | 12.03(5.78,26.19) | 1.15(0.55,2.51) |  | 4.96(2.67,8.20) | 0.84(0.45,1.38) |  | -58.79(-84.37,-6.60) | -0.28(-0.71,0.15) |
| Australia | 83.49(54.15,127.31) | 2.21(1.43,3.36) |  | 65.68(29.10,103.85) | 1.38(0.61,2.19) |  | -21.34(-68.01,37.43) | -1.54(-1.97,-1.11) |
| Austria | 30.87(20.48,46.53) | 2.29(1.52,3.45) |  | 22.38(11.25,33.01) | 1.73(0.87,2.55) |  | -27.50(-66.76,22.91) | -0.89(-1.30,-0.48) |
| Azerbaijan | 29.35(11.73,65.55) | 1.21(0.48,2.70) |  | 22.55(9.77,46.15) | 0.96(0.41,1.96) |  | -23.16(-70.83,109.38) | -1.50(-1.93,-1.07) |
| Bahamas | 5.48(3.69,8.17) | 6.80(4.58,10.13) |  | 4.39(2.56,6.63) | 5.41(3.16,8.16) |  | -19.89(-53.92,22.35) | -1.07(-1.37,-0.77) |
| Bahrain | 2.88(1.06,5.68) | 1.76(0.65,3.48) |  | 2.44(0.79,5.32) | 0.82(0.27,1.79) |  | -15.30(-77.00,108.86) | -3.09(-3.71,-2.46) |
| Bangladesh | 2037.42(546.58,5525.78) | 4.17(1.12,11.30) |  | 1566.56(526.60,3531.82) | 3.42(1.15,7.72) |  | -23.11(-83.17,254.61) | -1.37(-1.72,-1.02) |
| Barbados | 10.99(7.65,15.69) | 17.63(12.26,25.16) |  | 5.02(3.12,7.34) | 10.67(6.63,15.58) |  | -54.29(-73.75,-26.58) | -0.85(-1.33,-0.37) |
| Belarus | 67.47(42.71,103.72) | 2.81(1.78,4.31) |  | 32.31(10.57,66.00) | 2.05(0.67,4.18) |  | -52.11(-85.19,2.93) | -0.18(-1.67,1.33) |
| Belgium | 42.53(24.82,66.59) | 2.35(1.37,3.69) |  | 48.95(19.79,76.89) | 2.56(1.03,4.02) |  | 15.08(-61.90,132.19) | -0.07(-0.62,0.49) |
| Belize | 3.46(1.99,7.01) | 4.23(2.43,8.56) |  | 3.56(2.15,5.12) | 2.89(1.75,4.16) |  | 2.88(-58.48,68.76) | -0.64(-0.91,-0.38) |
| Benin | 1319.55(525.67,2258.33) | 54.49(21.71,93.25) |  | 3091.19(1597.66,4767.44) | 50.84(26.28,78.41) |  | 134.26(30.96,349.85) | 0.01(-0.19,0.20) |
| Bermuda | 0.92(0.48,1.47) | 7.72(4.00,12.33) |  | 0.42(0.15,0.70) | 4.92(1.77,8.26) |  | -54.85(-85.38,31.66) | -1.15(-1.49,-0.81) |
| Bhutan | 11.01(2.34,28.06) | 4.20(0.89,10.70) |  | 6.63(2.09,16.35) | 3.54(1.12,8.73) |  | -39.72(-89.38,344.92) | -1.47(-1.95,-0.99) |
| Bolivia (Plurinational State of) | 367.85(174.94,692.25) | 13.70(6.51,25.77) |  | 284.32(125.46,498.17) | 8.15(3.60,14.29) |  | -22.71(-75.98,80.28) | -2.52(-2.95,-2.09) |
| Bosnia and Herzegovina | 12.78(4.47,29.37) | 1.17(0.41,2.68) |  | 4.47(1.54,9.60) | 0.91(0.31,1.96) |  | -64.99(-91.46,20.21) | -1.22(-1.72,-0.72) |
| Botswana | 35.10(17.31,65.40) | 5.95(2.93,11.08) |  | 81.61(35.62,145.77) | 11.69(5.10,20.88) |  | 132.49(-3.50,398.18) | 2.59(2.33,2.86) |
| Brazil | 4730.45(3324.59,6813.99) | 9.11(6.40,13.12) |  | 3033.46(1635.78,4194.81) | 6.30(3.39,8.71) |  | -35.87(-69.82,-9.15) | -0.87(-1.43,-0.31) |
| Brunei Darussalam | 4.76(2.41,8.53) | 5.26(2.66,9.42) |  | 2.79(1.54,4.52) | 2.95(1.63,4.78) |  | -41.43(-74.58,26.28) | -1.67(-2.55,-0.78) |
| Bulgaria | 15.96(7.55,32.17) | 0.92(0.43,1.85) |  | 8.22(3.59,13.63) | 0.84(0.37,1.40) |  | -48.48(-84.19,41.05) | -0.50(-0.96,-0.04) |
| Burkina Faso | 2728.07(1122.73,4520.05) | 57.81(23.79,95.78) |  | 5432.07(2830.50,8454.24) | 52.37(27.29,81.51) |  | 99.12(21.36,259.97) | 0.05(-0.21,0.31) |
| Burundi | 2923.01(1130.43,5192.51) | 111.52(43.13,198.10) |  | 2629.00(1308.20,4658.17) | 44.91(22.35,79.57) |  | -10.06(-46.02,76.69) | -2.64(-2.85,-2.44) |
| Cabo Verde | 15.46(7.88,25.42) | 9.83(5.01,16.16) |  | 38.24(15.20,59.53) | 26.70(10.62,41.57) |  | 147.30(-17.39,379.27) | 1.71(0.74,2.68) |
| Cambodia | 118.29(22.12,367.85) | 2.54(0.47,7.89) |  | 77.55(31.79,152.17) | 1.52(0.62,2.97) |  | -34.45(-81.22,183.11) | -2.20(-2.44,-1.96) |
| Cameroon | 2653.04(1196.19,4283.57) | 54.34(24.50,87.74) |  | 7552.05(4208.56,11208.92) | 56.08(31.25,83.23) |  | 184.66(72.62,421.54) | 0.49(0.24,0.75) |
| Canada | 131.97(81.56,201.65) | 2.29(1.42,3.51) |  | 94.42(43.72,157.07) | 1.53(0.71,2.54) |  | -28.45(-69.02,20.49) | -1.74(-2.06,-1.41) |
| Central African Republic | 563.45(194.56,1015.63) | 46.08(15.91,83.07) |  | 788.17(352.13,1313.88) | 34.51(15.42,57.53) |  | 39.88(-14.37,165.22) | -0.78(-0.91,-0.65) |
| Chad | 1312.78(538.70,2237.97) | 44.86(18.41,76.48) |  | 4670.77(2423.72,7404.09) | 51.81(26.89,82.13) |  | 255.79(112.18,533.58) | 0.88(0.70,1.05) |
| Chile | 258.52(131.17,431.57) | 6.51(3.30,10.87) |  | 158.28(87.75,227.58) | 4.33(2.40,6.23) |  | -38.77(-73.18,35.94) | -0.89(-1.19,-0.59) |
| China | 11155.05(4608.97,16735.63) | 3.50(1.45,5.26) |  | 2612.17(1390.82,4429.74) | 1.01(0.54,1.71) |  | -76.58(-89.48,-38.35) | -5.41(-5.95,-4.87) |
| Colombia | 1005.97(634.72,1545.61) | 8.63(5.44,13.25) |  | 700.73(332.46,1239.48) | 6.60(3.13,11.68) |  | -30.34(-69.23,19.42) | -0.19(-0.55,0.18) |
| Comoros | 162.89(65.73,270.95) | 76.58(30.90,127.39) |  | 130.26(73.97,199.88) | 54.24(30.80,83.23) |  | -20.03(-60.92,63.96) | -1.37(-1.75,-0.98) |
| Congo | 351.31(138.11,591.69) | 33.36(13.12,56.19) |  | 372.83(203.88,556.92) | 19.32(10.57,28.87) |  | 6.13(-35.23,110.83) | -1.77(-2.02,-1.52) |
| Cook Islands | 0.05(0.02,0.11) | 0.78(0.32,1.63) |  | 0.04(0.01,0.08) | 0.93(0.15,2.08) |  | -31.09(-88.29,98.04) | -0.40(-0.77,-0.03) |
| Costa Rica | 79.69(43.02,127.42) | 7.09(3.83,11.33) |  | 58.11(21.57,108.43) | 5.71(2.12,10.66) |  | -27.08(-72.59,117.93) | -0.89(-1.13,-0.65) |
| Croatia | 3048.30(1454.32,4690.58) | 53.45(25.50,82.24) |  | 5507.64(2915.74,8586.26) | 47.60(25.20,74.20) |  | -47.13(-85.75,63.98) | 0.04(-0.17,0.26) |
| Cuba | 25.19(11.99,43.59) | 2.55(1.22,4.42) |  | 13.32(4.42,23.09) | 2.23(0.74,3.87) |  | -74.99(-87.92,-45.41) | 0.11(-0.47,0.70) |
| Cyprus | 358.52(219.01,568.31) | 14.32(8.75,22.70) |  | 89.68(49.34,152.67) | 5.05(2.78,8.59) |  | -43.32(-74.00,33.03) | -0.95(-1.77,-0.13) |
| Czechia | 7.83(3.81,14.46) | 3.95(1.93,7.31) |  | 4.44(2.48,7.35) | 2.03(1.13,3.36) |  | -53.82(-89.06,20.51) | -2.06(-2.81,-1.32) |
| C么te d'Ivoire | 50.00(32.66,80.20) | 2.27(1.48,3.64) |  | 23.09(6.25,48.70) | 1.35(0.36,2.84) |  | 80.68(7.85,202.26) | -1.68(-2.05,-1.31) |
| Democratic People's Republic of Korea | 130.85(54.95,292.39) | 2.20(0.92,4.91) |  | 71.72(25.91,152.14) | 1.50(0.54,3.19) |  | -45.19(-79.59,35.81) | -1.58(-1.76,-1.41) |
| Democratic Republic of the Congo | 7439.74(2578.50,13509.64) | 42.02(14.56,76.31) |  | 6743.11(3333.65,10710.24) | 17.75(8.77,28.19) |  | -9.36(-45.71,109.67) | -2.36(-2.57,-2.15) |
| Denmark | 31.24(19.95,46.80) | 3.54(2.26,5.30) |  | 14.70(7.05,23.62) | 1.54(0.74,2.48) |  | -52.95(-80.86,-23.24) | -2.66(-3.00,-2.32) |
| Djibouti | 102.81(50.07,163.99) | 59.05(28.76,94.19) |  | 206.27(112.48,335.68) | 49.92(27.22,81.24) |  | 100.63(14.65,256.64) | -0.52(-0.89,-0.14) |
| Dominica | 1.28(0.65,2.40) | 5.17(2.64,9.66) |  | 0.79(0.39,1.47) | 5.79(2.82,10.71) |  | -38.20(-70.97,45.01) | 0.81(0.53,1.09) |
| Dominican Republic | 100.12(52.18,212.79) | 3.71(1.94,7.89) |  | 136.91(58.79,284.64) | 4.66(2.00,9.69) |  | 36.75(-54.90,286.04) | 0.81(0.40,1.22) |
| Ecuador | 219.97(146.10,320.04) | 5.69(3.78,8.28) |  | 260.59(110.89,411.10) | 5.14(2.19,8.11) |  | 18.46(-55.30,95.47) | -0.17(-0.51,0.17) |
| Egypt | 646.88(147.33,2284.57) | 2.92(0.66,10.30) |  | 427.80(52.60,1715.10) | 1.16(0.14,4.65) |  | -33.87(-90.02,73.80) | -2.90(-4.22,-1.57) |
| El Salvador | 85.91(50.73,145.08) | 3.98(2.35,6.72) |  | 50.32(27.62,81.64) | 2.77(1.52,4.49) |  | -41.43(-75.14,6.69) | -1.39(-1.66,-1.12) |
| Equatorial Guinea | 84.21(31.33,149.30) | 42.76(15.91,75.82) |  | 121.16(50.44,241.35) | 20.71(8.62,41.26) |  | 43.88(-34.01,269.83) | -3.09(-3.43,-2.75) |
| Eritrea | 1188.83(506.33,1925.50) | 74.68(31.81,120.96) |  | 1476.91(774.84,2429.56) | 58.50(30.69,96.23) |  | 24.23(-29.71,141.52) | -0.86(-1.01,-0.72) |
| Estonia | 12.48(7.28,23.84) | 3.57(2.09,6.83) |  | 4.14(1.08,8.36) | 1.92(0.50,3.87) |  | -66.81(-93.86,-5.32) | -1.72(-2.02,-1.43) |
| Eswatini | 30.15(13.47,62.50) | 7.82(3.49,16.20) |  | 45.16(19.25,84.83) | 10.94(4.66,20.56) |  | 49.79(-35.40,252.70) | 1.70(1.37,2.02) |
| Ethiopia | 20952.91(6415.56,41335.78) | 86.00(26.33,169.66) |  | 17790.41(9430.82,27832.74) | 40.11(21.26,62.76) |  | -15.09(-53.58,86.50) | -2.71(-2.91,-2.51) |
| Fiji | 6.03(2.64,11.16) | 2.14(0.94,3.96) |  | 12.54(5.36,26.02) | 4.60(1.97,9.55) |  | 107.95(-22.32,525.00) | 2.87(2.51,3.23) |
| Finland | 25.85(14.82,41.71) | 2.68(1.54,4.32) |  | 10.81(3.97,18.52) | 1.28(0.47,2.19) |  | -58.19(-84.77,-21.57) | -2.52(-2.89,-2.14) |
| France | 257.02(164.97,389.25) | 2.19(1.41,3.32) |  | 232.68(72.97,485.54) | 2.00(0.63,4.18) |  | -9.47(-69.28,94.92) | -0.60(-0.94,-0.26) |
| Gabon | 108.23(48.58,172.31) | 26.56(11.92,42.28) |  | 137.76(73.37,222.98) | 21.55(11.48,34.89) |  | 27.28(-23.57,122.78) | -0.21(-0.39,-0.02) |
| Gambia | 220.11(103.68,368.79) | 47.72(22.48,79.95) |  | 400.72(215.29,663.63) | 40.34(21.67,66.80) |  | 82.06(-1.21,245.41) | -0.61(-0.91,-0.31) |
| Georgia | 113.19(31.85,204.31) | 8.27(2.33,14.93) |  | 16.44(7.16,26.42) | 2.23(0.97,3.59) |  | -85.47(-95.27,-34.80) | -4.18(-5.28,-3.06) |
| Germany | 222.62(134.04,363.55) | 1.72(1.04,2.81) |  | 148.31(56.22,263.03) | 1.24(0.47,2.20) |  | -33.38(-77.27,21.58) | -0.83(-1.46,-0.21) |
| Ghana | 6205.79(1951.33,11122.31) | 92.40(29.05,165.59) |  | 4256.57(2570.13,7439.23) | 33.04(19.95,57.74) |  | -31.41(-65.78,144.80) | -4.27(-4.97,-3.57) |
| Greece | 25.53(9.42,46.40) | 1.26(0.47,2.29) |  | 17.95(10.14,26.37) | 1.29(0.73,1.89) |  | -29.70(-67.10,101.79) | 0.15(-0.30,0.60) |
| Greenland | 1.13(0.16,2.62) | 7.96(1.10,18.44) |  | 0.19(0.06,0.42) | 1.59(0.53,3.57) |  | -83.51(-96.53,19.48) | -4.11(-4.75,-3.47) |
| Grenada | 4.31(2.61,7.10) | 12.91(7.82,21.24) |  | 2.15(1.29,3.37) | 9.84(5.93,15.44) |  | -50.20(-71.53,-22.75) | -0.37(-0.55,-0.19) |
| Guam | 0.59(0.26,1.24) | 1.40(0.62,2.97) |  | 1.16(0.67,1.76) | 3.17(1.84,4.82) |  | 98.09(-11.47,418.41) | 5.01(4.17,5.86) |
| Guatemala | 314.23(178.94,643.06) | 7.74(4.41,15.83) |  | 190.74(117.36,282.14) | 3.87(2.38,5.72) |  | -39.30(-75.90,11.38) | -1.64(-1.86,-1.42) |
| Guinea | 1628.71(680.88,2714.29) | 59.19(24.74,98.64) |  | 2291.55(1263.88,3972.58) | 37.90(20.91,65.71) |  | 40.70(-17.43,171.65) | -0.87(-1.12,-0.62) |
| Guinea-Bissau | 322.78(127.85,550.13) | 66.91(26.50,114.04) |  | 367.50(187.55,594.31) | 40.92(20.88,66.17) |  | 13.85(-30.74,132.12) | -1.26(-1.61,-0.90) |
| Guyana | 19.60(11.11,33.39) | 6.67(3.78,11.36) |  | 6.91(3.77,13.50) | 3.24(1.77,6.32) |  | -64.75(-83.73,-1.84) | -0.21(-1.05,0.63) |
| Haiti | 543.63(96.29,1817.56) | 20.04(3.55,66.99) |  | 684.52(182.55,1806.24) | 15.73(4.19,41.50) |  | 25.92(-48.86,278.39) | -0.48(-0.65,-0.31) |
| Honduras | 91.68(41.99,190.92) | 4.15(1.90,8.64) |  | 75.61(36.11,135.90) | 2.31(1.10,4.15) |  | -17.53(-66.04,103.11) | -2.31(-2.74,-1.88) |
| Hungary | 44.22(25.81,72.54) | 2.08(1.21,3.40) |  | 22.79(6.28,42.89) | 1.64(0.45,3.09) |  | -48.48(-86.20,48.36) | -0.46(-1.00,0.08) |
| Iceland | 1.14(0.65,1.82) | 1.79(1.03,2.87) |  | 0.51(0.26,0.86) | 0.75(0.39,1.28) |  | -55.37(-78.88,-17.26) | -1.91(-2.59,-1.23) |
| India | 14843.33(4631.90,29345.24) | 4.55(1.42,8.99) |  | 7359.75(4766.82,11346.60) | 2.01(1.30,3.10) |  | -50.42(-81.95,66.84) | -2.95(-3.11,-2.78) |
| Indonesia | 755.38(188.68,1648.74) | 1.12(0.28,2.43) |  | 805.48(393.23,1356.22) | 1.20(0.58,2.02) |  | 6.63(-48.90,170.44) | -1.26(-1.76,-0.75) |
| Iran (Islamic Republic of) | 520.74(240.59,1315.39) | 2.05(0.95,5.18) |  | 304.54(135.41,557.29) | 1.51(0.67,2.76) |  | -41.52(-88.52,90.08) | -1.11(-1.51,-0.71) |
| Iraq | 833.31(146.90,2079.32) | 10.12(1.78,25.25) |  | 394.12(132.07,854.48) | 2.93(0.98,6.35) |  | -52.70(-83.23,77.68) | -4.18(-4.52,-3.83) |
| Ireland | 17.41(10.59,28.02) | 1.77(1.08,2.85) |  | 16.13(6.38,27.49) | 1.62(0.64,2.76) |  | -7.33(-63.91,92.75) | -0.96(-1.49,-0.42) |
| Israel | 88.07(51.42,145.10) | 5.74(3.35,9.46) |  | 106.59(61.74,152.68) | 4.06(2.35,5.81) |  | 21.02(-39.98,101.96) | -0.95(-1.54,-0.35) |
| Italy | 219.99(152.24,363.79) | 2.38(1.65,3.94) |  | 165.89(58.37,312.48) | 2.18(0.77,4.11) |  | -24.59(-78.63,78.02) | 0.05(-0.39,0.49) |
| Jamaica | 47.59(26.30,85.37) | 5.70(3.15,10.22) |  | 20.94(11.61,36.72) | 3.59(1.99,6.29) |  | -56.00(-79.37,-20.38) | -1.16(-1.46,-0.85) |
| Japan | 348.52(147.33,602.13) | 1.51(0.64,2.61) |  | 232.45(98.88,321.20) | 1.51(0.64,2.08) |  | -33.30(-73.91,95.98) | -0.07(-0.51,0.38) |
| Jordan | 95.83(44.25,193.30) | 5.87(2.71,11.83) |  | 141.17(72.17,258.07) | 3.89(1.99,7.10) |  | 47.31(-40.59,267.54) | -2.06(-2.53,-1.59) |
| Kazakhstan | 136.67(67.72,261.79) | 2.63(1.30,5.04) |  | 61.52(28.34,110.09) | 1.13(0.52,2.03) |  | -54.99(-85.08,1.20) | -3.24(-3.79,-2.68) |
| Kenya | 2634.16(1376.78,3956.08) | 23.58(12.33,35.42) |  | 3563.48(2235.72,4825.38) | 19.09(11.98,25.85) |  | 35.28(-7.15,106.89) | 0.38(-0.08,0.84) |
| Kiribati | 0.09(0.03,0.21) | 0.29(0.09,0.70) |  | 0.11(0.03,0.25) | 0.26(0.06,0.60) |  | 24.51(-65.25,229.85) | -0.99(-1.34,-0.63) |
| Kuwait | 27.24(15.72,47.18) | 4.91(2.84,8.51) |  | 13.43(6.88,21.80) | 1.59(0.81,2.58) |  | -50.72(-76.55,-9.58) | -3.18(-3.77,-2.58) |
| Kyrgyzstan | 33.23(18.74,66.44) | 1.98(1.12,3.96) |  | 24.90(11.47,46.90) | 1.09(0.50,2.06) |  | -25.09(-75.94,77.58) | -2.18(-2.84,-1.53) |
| Lao People's Democratic Republic | 37.38(5.21,126.88) | 2.03(0.28,6.88) |  | 44.70(16.93,93.62) | 1.95(0.74,4.08) |  | 19.57(-67.15,618.37) | -2.44(-2.98,-1.91) |
| Latvia | 15.88(8.58,30.25) | 2.79(1.51,5.32) |  | 4.71(1.26,9.13) | 1.58(0.43,3.07) |  | -70.36(-94.41,-13.90) | -1.36(-1.66,-1.05) |
| Lebanon | 52.99(24.11,106.84) | 5.07(2.31,10.22) |  | 37.13(14.38,72.92) | 2.91(1.13,5.71) |  | -29.93(-77.76,111.22) | -1.81(-2.08,-1.53) |
| Lesotho | 36.45(17.61,68.48) | 5.34(2.58,10.03) |  | 64.69(27.37,124.81) | 10.26(4.34,19.80) |  | 77.47(-28.88,281.08) | 2.83(2.55,3.10) |
| Liberia | 848.89(363.33,1424.87) | 75.11(32.15,126.07) |  | 988.93(507.76,1488.30) | 45.24(23.23,68.09) |  | 16.50(-32.77,126.34) | -1.77(-2.28,-1.26) |
| Libya | 117.32(41.13,274.14) | 6.48(2.27,15.14) |  | 124.84(42.23,265.06) | 8.37(2.83,17.77) |  | 6.40(-58.87,203.97) | 1.21(0.87,1.54) |
| Lithuania | 22.10(11.94,36.38) | 2.66(1.44,4.38) |  | 8.33(2.49,18.75) | 2.04(0.61,4.60) |  | -62.32(-91.40,31.45) | -1.18(-1.59,-0.77) |
| Luxembourg | 2.01(0.82,3.32) | 3.04(1.24,5.02) |  | 2.60(1.45,3.94) | 2.57(1.43,3.89) |  | 29.40(-34.45,190.70) | -1.23(-1.59,-0.87) |
| Madagascar | 3699.48(1815.73,5544.03) | 67.81(33.28,101.61) |  | 4894.78(2982.45,7181.84) | 41.72(25.42,61.21) |  | 32.31(-18.87,123.45) | -1.08(-1.26,-0.90) |
| Malawi | 8209.82(3706.09,13704.60) | 180.45(81.46,301.23) |  | 8794.65(4041.99,17426.64) | 108.26(49.75,214.51) |  | 7.12(-39.60,105.45) | -1.32(-1.59,-1.04) |
| Malaysia | 98.03(42.97,210.31) | 1.49(0.65,3.20) |  | 63.74(20.22,144.53) | 0.84(0.27,1.90) |  | -34.98(-82.20,71.77) | -2.43(-3.09,-1.78) |
| Maldives | 4.71(1.32,12.95) | 4.49(1.26,12.33) |  | 1.74(0.85,3.21) | 1.74(0.85,3.21) |  | -63.08(-91.00,45.80) | -3.13(-3.37,-2.90) |
| Mali | 1783.55(809.50,2910.55) | 43.19(19.60,70.48) |  | 3171.20(1775.91,5106.60) | 27.40(15.34,44.11) |  | 77.80(-1.47,238.30) | -1.12(-1.36,-0.88) |
| Malta | 1.97(1.01,3.23) | 2.25(1.16,3.69) |  | 2.04(1.09,3.79) | 3.19(1.70,5.92) |  | 3.54(-44.06,193.10) | 0.30(-0.33,0.92) |
| Marshall Islands | 0.18(0.08,0.38) | 0.84(0.38,1.71) |  | 0.37(0.14,0.81) | 2.12(0.78,4.65) |  | 102.05(-26.80,433.61) | 2.56(2.08,3.04) |
| Mauritania | 350.72(173.38,570.91) | 37.94(18.76,61.76) |  | 568.45(333.77,855.94) | 30.68(18.01,46.19) |  | 62.08(-0.56,190.69) | -0.87(-1.20,-0.53) |
| Mauritius | 2.75(1.89,3.91) | 0.83(0.57,1.18) |  | 1.52(0.84,2.25) | 0.73(0.41,1.09) |  | -44.71(-69.12,-16.37) | -0.54(-0.80,-0.29) |
| Mexico | 1867.04(1236.22,3127.76) | 5.59(3.70,9.36) |  | 1330.22(679.59,1917.51) | 4.15(2.12,5.98) |  | -28.75(-70.53,10.48) | -0.60(-0.96,-0.24) |
| Micronesia (Federated States of) | 0.40(0.15,0.88) | 0.87(0.32,1.92) |  | 0.54(0.19,1.25) | 1.78(0.61,4.08) |  | 35.66(-59.58,357.07) | 2.66(2.43,2.89) |
| Monaco | 0.02(0.01,0.06) | 0.66(0.17,1.76) |  | 0.04(0.01,0.09) | 0.77(0.24,1.82) |  | 66.82(-34.01,400.27) | 0.14(-0.45,0.73) |
| Mongolia | 23.21(6.04,65.55) | 2.58(0.67,7.28) |  | 30.59(12.86,60.18) | 2.82(1.18,5.54) |  | 31.81(-67.44,462.32) | -0.06(-0.35,0.23) |
| Montenegro | 7.06(4.54,11.31) | 4.37(2.81,7.00) |  | 2.03(0.91,3.78) | 1.82(0.81,3.39) |  | -71.25(-88.02,-39.13) | -2.73(-3.15,-2.30) |
| Morocco | 485.69(171.62,1121.18) | 4.96(1.75,11.46) |  | 306.94(133.33,570.17) | 3.13(1.36,5.82) |  | -36.80(-78.64,71.95) | -1.19(-1.38,-1.00) |
| Mozambique | 936.45(355.50,2208.09) | 15.09(5.73,35.59) |  | 980.66(417.11,2465.06) | 6.87(2.92,17.28) |  | 4.72(-46.05,113.24) | -2.14(-2.39,-1.88) |
| Myanmar | 444.65(65.54,1494.08) | 3.01(0.44,10.11) |  | 263.98(109.07,529.16) | 1.69(0.70,3.39) |  | -40.63(-82.91,157.08) | -2.53(-2.80,-2.25) |
| Namibia | 57.67(28.91,101.68) | 9.60(4.81,16.93) |  | 126.41(64.16,217.62) | 15.31(7.77,26.36) |  | 119.21(4.46,366.26) | 2.12(1.86,2.37) |
| Nauru | 0.08(0.04,0.17) | 1.93(0.88,4.05) |  | 0.15(0.04,0.33) | 3.72(1.03,8.37) |  | 81.76(-36.52,363.65) | 2.15(1.74,2.55) |
| Nepal | 282.77(83.89,672.96) | 3.36(1.00,7.99) |  | 259.55(80.25,633.19) | 2.81(0.87,6.86) |  | -8.21(-80.79,287.05) | -1.16(-1.37,-0.95) |
| Netherlands | 109.19(69.22,164.03) | 4.01(2.54,6.02) |  | 75.96(35.27,111.78) | 2.83(1.32,4.17) |  | -30.43(-70.74,10.75) | -1.08(-1.40,-0.76) |
| New Zealand | 26.45(18.53,38.69) | 3.31(2.32,4.84) |  | 19.70(12.85,27.83) | 2.01(1.31,2.83) |  | -25.52(-57.44,9.96) | -2.10(-3.15,-1.04) |
| Nicaragua | 125.06(70.87,221.91) | 6.87(3.89,12.18) |  | 80.35(50.81,125.80) | 4.06(2.57,6.35) |  | -35.75(-68.86,27.49) | -1.68(-1.96,-1.40) |
| Niger | 3011.46(1109.87,5563.62) | 74.12(27.32,136.94) |  | 5078.66(2309.55,8423.72) | 39.79(18.09,66.00) |  | 68.64(-4.15,245.69) | -1.83(-2.05,-1.62) |
| Nigeria | 24699.95(12196.10,36912.73) | 63.13(31.17,94.35) |  | 52225.41(26224.00,74292.58) | 51.41(25.81,73.13) |  | 111.44(54.27,211.15) | -0.36(-0.51,-0.20) |
| Niue | 0.01(0.00,0.03) | 1.54(0.60,3.26) |  | 0.02(0.00,0.04) | 3.99(1.21,9.79) |  | 24.25(-58.26,268.08) | 2.49(2.25,2.73) |
| North Macedonia | 3.18(1.31,7.72) | 0.60(0.25,1.47) |  | 1.71(0.75,3.26) | 0.52(0.23,1.00) |  | -46.24(-89.06,74.11) | -0.17(-0.80,0.45) |
| Northern Mariana Islands | 0.06(0.02,0.14) | 0.47(0.13,1.19) |  | 0.10(0.04,0.19) | 0.86(0.36,1.65) |  | 68.89(-41.82,640.17) | 3.40(2.50,4.31) |
| Norway | 10.95(7.36,19.00) | 1.37(0.92,2.38) |  | 6.58(2.86,10.43) | 0.71(0.31,1.13) |  | -39.96(-79.09,-9.83) | -2.04(-2.59,-1.49) |
| Oman | 32.12(11.72,69.31) | 3.82(1.39,8.25) |  | 36.24(12.01,69.24) | 2.96(0.98,5.66) |  | 12.83(-59.92,208.31) | -0.62(-0.90,-0.33) |
| Pakistan | 5611.12(2216.21,10336.85) | 11.39(4.50,20.99) |  | 13620.84(5488.00,25785.46) | 15.94(6.42,30.18) |  | 142.75(20.01,364.94) | 1.20(1.03,1.37) |
| Palau | 0.07(0.03,0.14) | 1.57(0.74,3.02) |  | 0.05(0.03,0.09) | 1.61(0.86,2.80) |  | -26.69(-67.19,57.76) | 0.33(0.15,0.50) |
| Palestine | 33.06(13.01,72.55) | 3.41(1.34,7.49) |  | 40.08(13.62,83.14) | 2.15(0.73,4.45) |  | 21.25(-68.54,242.34) | -1.24(-1.62,-0.86) |
| Panama | 75.92(49.73,115.98) | 9.10(5.96,13.91) |  | 98.73(63.85,136.89) | 8.56(5.54,11.87) |  | 30.04(-22.22,90.99) | -0.30(-0.45,-0.14) |
| Papua New Guinea | 32.74(8.37,85.93) | 1.93(0.49,5.05) |  | 134.05(32.51,311.84) | 3.42(0.83,7.96) |  | 309.51(69.96,978.91) | 1.98(1.57,2.39) |
| Paraguay | 77.44(42.51,133.08) | 4.64(2.55,7.97) |  | 80.37(36.22,142.11) | 4.00(1.80,7.08) |  | 3.79(-55.31,120.68) | -0.39(-0.80,0.03) |
| Peru | 881.25(473.93,1527.75) | 10.62(5.71,18.40) |  | 547.21(272.89,946.83) | 5.74(2.86,9.93) |  | -37.90(-76.52,42.55) | -2.13(-2.31,-1.95) |
| Philippines | 762.94(303.57,1275.26) | 3.03(1.20,5.06) |  | 601.14(377.02,874.60) | 1.77(1.11,2.57) |  | -21.21(-57.88,58.98) | -1.41(-1.61,-1.21) |
| Poland | 148.09(46.17,298.87) | 1.55(0.48,3.12) |  | 93.80(35.19,134.61) | 1.59(0.60,2.29) |  | -36.67(-83.71,145.64) | 0.03(-0.80,0.87) |
| Portugal | 70.63(40.76,142.97) | 3.34(1.93,6.76) |  | 32.94(12.77,54.52) | 2.42(0.94,4.00) |  | -53.36(-88.63,-13.00) | -1.41(-1.79,-1.03) |
| Puerto Rico | 84.48(45.38,133.03) | 8.48(4.56,13.36) |  | 19.04(8.35,28.47) | 4.28(1.88,6.41) |  | -77.47(-90.68,-57.49) | -1.82(-2.04,-1.60) |
| Qatar | 1.45(0.47,3.49) | 1.16(0.38,2.79) |  | 3.82(1.24,8.09) | 0.77(0.25,1.64) |  | 162.87(-4.58,590.87) | -1.54(-1.97,-1.10) |
| Republic of Korea | 184.91(58.58,391.03) | 1.63(0.52,3.44) |  | 42.50(16.98,83.99) | 0.70(0.28,1.38) |  | -77.02(-93.54,-19.96) | -2.80(-3.12,-2.49) |
| Republic of Moldova | 160.55(78.68,315.11) | 12.99(6.37,25.50) |  | 28.23(14.81,44.64) | 5.41(2.84,8.55) |  | -82.42(-93.01,-59.76) | -1.93(-2.52,-1.34) |
| Romania | 233.99(92.32,531.94) | 4.20(1.66,9.55) |  | 96.50(52.80,150.28) | 3.21(1.75,4.99) |  | -58.76(-87.15,15.13) | -0.73(-1.12,-0.33) |
| Russian Federation | 1553.46(927.58,2334.83) | 4.48(2.67,6.73) |  | 514.95(227.95,738.35) | 1.97(0.87,2.83) |  | -66.85(-86.50,-47.77) | -2.21(-2.57,-1.84) |
| Rwanda | 4029.55(1693.48,6466.73) | 118.76(49.91,190.60) |  | 2555.84(1465.14,4056.85) | 51.42(29.48,81.62) |  | -36.57(-63.89,23.88) | -3.30(-3.60,-3.00) |
| Saint Kitts and Nevis | 0.47(0.28,0.82) | 3.36(2.02,5.78) |  | 0.32(0.18,0.60) | 3.28(1.86,6.10) |  | -31.94(-65.18,65.01) | 0.76(0.42,1.11) |
| Saint Lucia | 3.20(1.97,4.96) | 6.22(3.82,9.63) |  | 1.72(1.01,2.53) | 5.78(3.41,8.52) |  | -46.42(-69.80,-6.47) | -0.23(-0.51,0.04) |
| Saint Vincent and the Grenadines | 2.70(0.77,5.16) | 6.57(1.87,12.55) |  | 1.53(0.94,2.36) | 6.13(3.77,9.44) |  | -43.36(-71.36,141.88) | -0.29(-0.56,-0.02) |
| Samoa | 0.76(0.27,1.91) | 1.07(0.37,2.68) |  | 1.01(0.35,2.15) | 1.26(0.44,2.69) |  | 32.31(-62.01,318.67) | 0.23(0.01,0.45) |
| San Marino | 0.16(0.06,0.35) | 3.89(1.50,8.62) |  | 0.14(0.05,0.30) | 3.20(1.05,6.76) |  | -11.80(-69.26,137.94) | -0.61(-0.96,-0.26) |
| Sao Tome and Principe | 34.15(15.35,56.21) | 60.27(27.09,99.19) |  | 17.88(10.43,28.20) | 22.98(13.40,36.23) |  | -47.63(-71.81,12.79) | -2.53(-2.89,-2.18) |
| Saudi Arabia | 322.98(149.96,620.19) | 4.93(2.29,9.46) |  | 187.14(48.83,362.97) | 2.47(0.65,4.80) |  | -42.06(-85.04,51.56) | -2.35(-2.54,-2.15) |
| Senegal | 2093.75(908.66,3282.75) | 57.34(24.89,89.91) |  | 2171.59(1337.19,3258.75) | 34.14(21.02,51.23) |  | 3.72(-39.00,89.73) | -1.58(-1.86,-1.30) |
| Serbia | 41.49(16.95,88.45) | 1.91(0.78,4.08) |  | 11.07(3.60,24.13) | 0.83(0.27,1.82) |  | -73.32(-93.06,-23.29) | -2.87(-3.14,-2.59) |
| Seychelles | 0.00(0.00,0.00) | 0.00(0.00,0.01) |  | 0.00(0.00,0.00) | 0.00(0.00,0.01) |  | 33.78(-67.08,597.28) | 0.62(0.18,1.06) |
| Sierra Leone | 1290.35(530.63,2206.14) | 71.19(29.27,121.71) |  | 1835.25(966.38,2833.15) | 51.32(27.02,79.22) |  | 42.23(-17.96,171.46) | -1.03(-1.23,-0.83) |
| Singapore | 13.98(7.65,27.25) | 2.15(1.18,4.20) |  | 11.80(3.69,20.39) | 1.45(0.45,2.51) |  | -15.61(-79.84,115.35) | -1.15(-2.00,-0.29) |
| Slovakia | 16.34(7.63,32.17) | 1.23(0.58,2.43) |  | 13.28(5.44,29.09) | 1.55(0.64,3.40) |  | -18.70(-73.89,140.29) | 0.68(-0.03,1.40) |
| Slovenia | 5.94(3.73,10.02) | 1.44(0.90,2.42) |  | 1.64(0.45,2.81) | 0.53(0.14,0.90) |  | -72.32(-93.57,-46.41) | -2.64(-3.38,-1.89) |
| Solomon Islands | 1.51(0.54,3.43) | 0.97(0.34,2.20) |  | 4.78(1.83,10.68) | 1.84(0.70,4.11) |  | 216.29(17.06,821.11) | 1.98(1.51,2.44) |
| Somalia | 2552.42(970.75,4892.41) | 65.52(24.92,125.59) |  | 4735.86(2025.99,8266.70) | 45.85(19.61,80.03) |  | 85.54(6.75,251.58) | -0.99(-1.37,-0.62) |
| South Africa | 405.12(197.72,641.25) | 2.98(1.45,4.71) |  | 560.01(362.58,844.65) | 3.68(2.38,5.55) |  | 38.23(-20.52,180.61) | 0.48(0.10,0.85) |
| South Sudan | 2109.19(945.34,3526.56) | 80.38(36.02,134.39) |  | 4109.28(1994.50,6563.24) | 95.68(46.44,152.81) |  | 94.83(26.94,219.78) | 0.68(-0.02,1.38) |
| Spain | 242.23(144.26,392.97) | 3.09(1.84,5.01) |  | 153.57(54.87,283.35) | 2.37(0.85,4.37) |  | -36.60(-80.89,21.72) | -1.00(-1.35,-0.66) |
| Sri Lanka | 150.42(72.34,317.69) | 2.72(1.31,5.74) |  | 87.07(33.26,174.29) | 1.71(0.65,3.41) |  | -42.11(-81.65,43.25) | -1.27(-1.76,-0.78) |
| Sudan | 566.28(149.15,1954.20) | 6.37(1.68,21.98) |  | 797.94(236.98,1985.04) | 4.81(1.43,11.97) |  | 40.91(-56.42,314.42) | -0.79(-0.86,-0.73) |
| Suriname | 3.76(1.80,7.05) | 2.88(1.38,5.41) |  | 5.06(2.35,10.01) | 3.53(1.64,6.98) |  | 34.71(-42.23,238.61) | 1.37(0.84,1.91) |
| Sweden | 15.39(4.49,28.78) | 1.00(0.29,1.86) |  | 19.76(11.26,30.82) | 1.09(0.62,1.69) |  | 28.43(-46.79,262.09) | 0.65(-0.52,1.84) |
| Switzerland | 23.19(13.75,39.27) | 2.01(1.19,3.40) |  | 14.44(6.16,26.14) | 1.08(0.46,1.96) |  | -37.72(-80.02,36.61) | -2.21(-2.63,-1.78) |
| Syrian Arab Republic | 78.59(39.19,152.97) | 1.33(0.66,2.58) |  | 60.00(20.79,112.59) | 1.64(0.57,3.07) |  | -23.65(-77.33,82.96) | 0.03(-0.90,0.96) |
| Taiwan (Province of China) | 95.86(64.03,159.50) | 1.74(1.16,2.90) |  | 40.27(13.60,69.93) | 1.37(0.46,2.37) |  | -57.99(-87.32,-27.06) | -0.05(-0.54,0.43) |
| Tajikistan | 3.16(0.71,8.69) | 0.14(0.03,0.37) |  | 2.96(1.23,6.34) | 0.08(0.03,0.18) |  | -6.30(-72.76,236.30) | -2.52(-2.92,-2.13) |
| Thailand | 292.24(61.74,658.37) | 1.73(0.37,3.91) |  | 139.76(58.38,273.55) | 1.43(0.60,2.80) |  | -52.18(-80.90,58.63) | -2.04(-2.56,-1.51) |
| Timor-Leste | 6.72(0.88,20.70) | 2.02(0.26,6.22) |  | 6.34(2.57,14.53) | 1.22(0.49,2.79) |  | -5.59(-71.61,301.48) | -2.25(-2.53,-1.96) |
| Togo | 826.72(382.25,1291.02) | 46.91(21.69,73.25) |  | 1255.95(696.13,1956.75) | 37.96(21.04,59.13) |  | 51.92(-10.62,189.90) | -0.43(-0.63,-0.23) |
| Tokelau | 0.01(0.00,0.01) | 1.18(0.50,2.34) |  | 0.01(0.00,0.02) | 2.51(0.79,5.81) |  | 38.69(-60.61,315.63) | 1.20(0.69,1.72) |
| Tonga | 1.31(0.58,2.60) | 3.14(1.40,6.23) |  | 1.83(0.70,4.34) | 4.68(1.80,11.12) |  | 39.20(-47.57,257.27) | 0.78(0.42,1.15) |
| Trinidad and Tobago | 34.10(20.58,53.90) | 8.39(5.06,13.26) |  | 15.19(8.87,22.40) | 5.58(3.26,8.22) |  | -55.45(-78.06,-22.73) | -0.83(-1.17,-0.48) |
| Tunisia | 218.18(76.77,512.53) | 7.03(2.47,16.50) |  | 111.70(42.82,221.00) | 4.04(1.55,7.99) |  | -48.80(-84.14,48.63) | -1.80(-1.92,-1.67) |
| Turkey | 3470.69(1584.61,6327.15) | 16.94(7.73,30.88) |  | 1371.88(828.84,2108.73) | 7.41(4.48,11.39) |  | -60.47(-82.81,-7.72) | -2.90(-3.20,-2.60) |
| Turkmenistan | 15.25(5.85,41.44) | 1.02(0.39,2.76) |  | 11.32(5.93,20.56) | 0.74(0.39,1.35) |  | -25.77(-69.56,79.76) | -1.30(-1.75,-0.85) |
| Tuvalu | 0.09(0.03,0.21) | 2.57(0.76,5.95) |  | 0.08(0.03,0.15) | 2.03(0.79,4.15) |  | -15.29(-73.88,141.26) | -0.41(-0.69,-0.13) |
| Uganda | 10061.73(5585.88,14838.84) | 119.50(66.34,176.24) |  | 22114.49(12600.67,34108.26) | 111.49(63.53,171.95) |  | 119.79(42.94,247.20) | -0.19(-0.48,0.10) |
| Ukraine | 288.86(133.38,614.89) | 2.54(1.17,5.41) |  | 146.03(52.17,304.75) | 2.30(0.82,4.80) |  | -49.44(-80.01,-0.61) | -0.58(-0.87,-0.30) |
| United Arab Emirates | 29.06(12.78,57.29) | 4.93(2.17,9.72) |  | 25.91(11.12,53.11) | 1.94(0.83,3.97) |  | -10.82(-63.57,90.24) | -3.02(-3.31,-2.74) |
| United Kingdom | 169.75(45.75,320.36) | 1.55(0.42,2.93) |  | 163.41(75.96,251.79) | 1.39(0.64,2.14) |  | -3.74(-67.25,187.70) | -0.11(-0.83,0.61) |
| United Republic of Tanzania | 11055.09(4685.30,16650.89) | 91.55(38.80,137.89) |  | 14389.59(8158.79,21865.64) | 58.97(33.43,89.60) |  | 30.16(-20.38,130.89) | -0.98(-1.19,-0.76) |
| United States of America | 1636.34(1147.77,2215.08) | 2.93(2.05,3.96) |  | 1136.37(717.87,1561.28) | 1.91(1.21,2.63) |  | -30.55(-55.85,-7.95) | -1.09(-1.37,-0.81) |
| United States Virgin Islands | 0.90(0.37,1.80) | 2.83(1.15,5.63) |  | 0.20(0.07,0.41) | 1.50(0.54,3.10) |  | -77.72(-91.91,-42.24) | -1.10(-1.57,-0.64) |
| Uruguay | 78.44(52.00,114.17) | 9.58(6.35,13.95) |  | 49.69(29.93,72.52) | 7.53(4.54,11.00) |  | -36.65(-64.41,9.40) | -0.91(-1.32,-0.49) |
| Uzbekistan | 64.46(31.54,136.17) | 0.75(0.37,1.59) |  | 71.85(36.63,158.94) | 0.71(0.36,1.58) |  | 11.47(-46.42,127.17) | -0.12(-0.31,0.07) |
| Vanuatu | 0.51(0.20,1.16) | 0.75(0.30,1.70) |  | 1.58(0.58,3.54) | 1.36(0.50,3.04) |  | 210.57(13.76,759.83) | 2.10(1.55,2.64) |
| Venezuela (Bolivarian Republic of) | 502.92(230.93,838.02) | 7.09(3.26,11.81) |  | 786.68(517.68,1108.26) | 11.88(7.82,16.73) |  | 56.42(-7.21,199.65) | 0.95(0.60,1.31) |
| Viet Nam | 414.26(107.35,990.91) | 1.56(0.40,3.74) |  | 593.71(119.15,1468.49) | 2.40(0.48,5.93) |  | 43.32(-58.97,236.93) | 1.55(1.34,1.76) |
| Yemen | 303.78(78.95,900.49) | 4.28(1.11,12.69) |  | 466.76(130.60,1227.83) | 3.39(0.95,8.90) |  | 53.65(-54.24,320.28) | -0.71(-0.84,-0.58) |
| Zambia | 3408.46(1339.34,5348.65) | 90.78(35.67,142.46) |  | 4312.20(2222.17,7107.89) | 52.13(26.87,85.93) |  | 26.51(-28.49,131.15) | -1.79(-1.96,-1.62) |
| Zimbabwe | 434.36(185.17,787.55) | 9.02(3.84,16.35) |  | 1433.59(509.33,2514.31) | 22.78(8.09,39.95) |  | 230.05(60.32,520.79) | 4.63(3.74,5.52) |
